# Telerehabilitation and Its Impact Following Stroke: An Umbrella Review of Systematic Reviews

**DOI:** 10.3390/jcm14010050

**Published:** 2024-12-26

**Authors:** Bayan Alwadai, Hatem Lazem, Hajar Almoajil, Abigail J. Hall, Maedeh Mansoubi, Helen Dawes

**Affiliations:** 1 Department of Public Health and Sport Sciences, Faculty of Health and Life Sciences, Medical School, University of Exeter, Exeter EX1 2LU, UK; hl756@exeter.ac.uk (H.L.); a.hall4@exeter.ac.uk (A.J.H.); m.mansoubi@exeter.ac.uk (M.M.); h.dawes@exeter.ac.uk (H.D.); 2 Physical Therapy Department, College of Applied Medical Sciences, Najran University, Najran 11001, Saudi Arabia; 3 Basic Science Department, Faculty of Physical Therapy, Cairo University, Cairo 12613, Egypt; 4 Physical Therapy Department, College of Applied Medical Sciences, Imam Abdulrahman bin Faisal University, Dammam 34212, Saudi Arabia; halmoajil@iau.edu.sa; 5 Exeter NIHR BRC Medical School, Faculty of Health and Life Sciences, University of Exeter, Exeter EX1 2LU, UK

**Keywords:** telerehabilitation, stroke, motor function, balance, gait, activities of daily living, quality of life, satisfaction, adherence to treatment, cost-effectiveness

## Abstract

**Objectives**: To summarize the impact of various telerehabilitation interventions on motor function, balance, gait, activities of daily living (ADLs), and quality of life (QoL) among patients with stroke and to determine the existing telerehabilitation interventions for delivering physiotherapy sessions in clinical practice. **Methods**: Six electronic databases were searched to identify relevant quantitative systematic reviews (SRs). Due to substantial heterogeneity, the data were analysed narratively. **Results**: A total of 28 systematic reviews (*n* = 245 primary studies) were included that examined various telerehabilitation interventions after stroke. Motor function was the most studied outcome domain across the reviews (20 SRs), followed by ADL (18 SRs), and balance (14 SRs) domains. For primary outcomes, our findings highlight moderate- to high-quality evidence showing either a significant effect or no significant difference between telerehabilitation and other interventions. There was insufficient evidence to draw a conclusion regarding feasibility outcomes, including participant satisfaction, adherence to treatment, and cost. Most reviews under this umbrella included patients with stroke in the subacute or chronic phase (12 SRs). Simple and complex telerehabilitation interventions such as telephone calls, videoconferencing, smartphone- or tablet-based mobile health applications, messaging, virtual reality, robot-assisted devices, and 3D animation videos, either alone or in combination with other interventions, were included across reviews. **Conclusions**: Various telerehabilitation interventions have shown either a significant effect or no significant difference compared to other interventions in improving upper and lower limb motor function, balance, gait, ADLs, and QoL, regardless of whether simple or complex approaches were used. Further research is needed to support the delivery of rehabilitation services through telerehabilitation intervention following a stroke.

## 1. Introduction

Stroke is a major cause of death and disability, affecting over nine million people annually on a worldwide basis [1,2]. Stroke survivors experience impairments in motor skills, balance, vision, sensation, cognition, swallowing, speech, and language. Motor impairment, or restricted function in mobility or muscular movement, is the most common impairment resulting from a stroke [3,4]. These motor impairments lead to functional dependence and decreased quality of life (QoL) [3]. Numerous studies have shown that effective rehabilitation programs, underpinned by targeted repeated practice, may decrease long-term disability, reduce the symptoms of a stroke, and enhance quality of life [5,6,7,8]. Rehabilitation services are often given in a clinical environment by medical professionals [5]. Patients, especially those living in rural places, however, find this challenging and must use additional resources and time to travel an extensive distance for both assessment and treatment. Thus, there is growing interest in alternative approaches for continuous, cost-effective, and accessible remote stroke rehabilitation programs, such as telerehabilitation [9].

Telerehabilitation is a branch of telemedicine that provides rehabilitation services remotely by utilizing information and communication technology including videoconferencing, internet-based media or programs, smartphones, mobile applications, telephones, data transmission by photos, and video or email from the client or healthcare professional [10,11]. Additionally, telerehabilitation can deliver therapy by using extended reality (XR)technologies, including mixed reality (MR), augmented reality (AR), and virtual reality (VR), which tracks users’ motions with computer software and lets them engage with a scenario or game that is displayed on a television screen [12,13]. Synchronous or asynchronous delivery of telerehabilitation is possible based on the patient’s needs, treatment programs, and medical conditions [14]. In the synchronous telerehabilitation approach, patients take part in their exercise sessions concurrently under supervision and make use of videoconferencing technology whereas, with asynchronous telerehabilitation methods, patients can use technological devices at their convenience to access their exercise programs [15]. These can include assessment, evaluation, monitoring, intervention, prevention, consultation, education, supervision, and coaching [5]. As such, telerehabilitation methods have the potential to increase access to high-intensity therapy [16].

Based on preliminary searches, several systematic reviews have been conducted on the effectiveness of telerehabilitation interventions on motor function, balance, gait, activities of daily living (ADLs), and quality of life (QoL) after stroke. However, to date, the effectiveness of different telerehabilitation interventions and delivery methods post-stroke have not been synthesized. Here we bring together available evidence from systematic reviews within an umbrella review with an aim to determine effective telerehabilitation interventions after stroke. Our review questions are as follows: (a) To what extent are different telerehabilitation interventions effective for patients living with stroke in terms of motor function, balance, gait, activities of daily living (ADLs), and quality of life? (b) What are the existing telerehabilitation interventions for delivering physiotherapy sessions in clinical practice?

## 2. Materials and Methods

### 2.1. Protocol and Registration

The study protocol was registered in the International Prospective Register of Systematic Reviews (PROSPERO: CRD42023468000). This umbrella review was carried out following the Joanna Briggs Institute (JBI) methodology for umbrella reviews [17]. The manuscript was written using the Preferred Reporting Items for Systematic Reviews and Meta-Analyses (PRISMA) guidelines [18].

### 2.2. Criteria for Considering Studies for This Umbrella Review

#### 2.2.1. Type of SRs

This umbrella review includes quantitative systematic reviews (with or without meta-analysis) and mixed-methods systematic reviews (quantitative elements only) that investigate the effectiveness of telerehabilitation interventions on motor function, balance, gait, activities of daily living (ADLs), and quality of life (QoL) in patients with stroke. Based on JBI methodology, if the purpose of an umbrella review is to evaluate the effectiveness of various interventions, it includes only quantitative systematic reviews [17].

#### 2.2.2. Types of Participants

This umbrella review considered adults with a stroke aged (≥18 years) with all types of strokes at any stage (acute, subacute, and chronic). Reviews included people with stroke (with other Neurological conditions e.g., Multiple sclerosis, and Parkinson’s’ disease) were excluded as this Umbrella focuses only on stroke conditions.

#### 2.2.3. Types of Interventions

A wide variety of therapeutic interventions within the scope of physiotherapy provided by telecommunication technology (the internet, the telephone, videoconferencing, mobile health applications (mHealth apps), etc.), and virtual reality-based telerehabilitation were included to get a complete picture of the existing and most effective strategies. More than one session must have been required to classify as an intervention. Programs that use a combination of in-person rehabilitation and telerehabilitation were included. Reviews focused on home-based rehabilitation programs without using telerehabilitation interventions were excluded.

#### 2.2.4. Types of Comparators

All comparisons of interest were included.

#### 2.2.5. Types of Outcome Measures

Primary outcomes include motor function of the upper and lower limbs, balance, gait, activities of daily living (ADLs), and quality of life (QoL). Feasibility outcomes: adherence to treatment, patient satisfaction, and cost-effectiveness.

### 2.3. Search Methods for the Identification of Studies

A scoping search of the CINAHL database was conducted initially to identify relevant systematic reviews related to the umbrella review topic and question. Several systematic reviews have been conducted on this topic, but no umbrella review has been yet performed. A comprehensive search was undertaken on the following electronic databases: Medline (Ovid), PEDro, CINAHL, Web of Science, Embase (Ovid), and Cochrane Library from the date of inception until December 2023. The search terms can be found in (Tables S1 and S2). The reference lists of all included reviews were checked. The search was limited to English-language reviews only.

### 2.4. Data Collection Process and Analysis

#### 2.4.1. Selection of SRs

All searched studies were exported to the master reference management library Rayyan, and duplicates were removed. The titles and abstracts of the studies were screened independently by two reviewers (BA and HL) against inclusion criteria. After that, two independent reviewers (BA and HL) screened the full text of relevant reviews. All disagreements between them were resolved through a discussion with a team (HD, MM, and HA).

#### 2.4.2. Methodological Quality Assessment of SRs

Two reviewers (BA and HL) independently assessed the quality of the review using the JBI Critical Appraisal Checklist for Systematic Reviews and Research Syntheses [19]. In case of no consensus, differences were resolved through discussion with a team (HD, MM, and HA). The JBI Checklist contains 11 questions. Each question answered by a yes can receive one point. The overall quality score ranges from 1 to 11. Reviews receiving one to four points are categorized as low-quality, five to seven points as medium-quality, and eight to 11 points as high-quality reviews. All reviews were included regardless of the methodological quality.

#### 2.4.3. Data Extraction and Management

The data were extracted from the included systematic reviews by two independent reviewers (BA and HL) using the JBI Data Extraction tool for Systematic Reviews and Research Syntheses, implemented using an Excel sheet. The extracted data included: citation details; objectives of the included reviews; type of reviews; search details; the number, type, date range, and country of origin of relevant primary studies; participant characteristics; total number of participants; intervention details; control group information; critical appraisal and rating tools; outcome measures; effect size, confidence interval and heterogeneity; methods of analysis; and findings.

#### 2.4.4. Managing Overlap of Primary Studies

A Matrix of Evidence table was created to demonstrate the degree of overlap of primary studies between the included systematic reviews. A separate Excel sheet listed all primary studies in the rows and all systematic reviews included in the umbrella review in the columns. Each primary study’s occurrence in a review was marked with an “∆” in the spreadsheet (Table S3). Then, we calculated the corrected covered area (CCA) using Pieper’s formula [20]:

CCA = N − r/(r × c) − r.

(N is the number of included publications, involving double counting, r is the number of index publications, and C is the number of reviews). A CCA value of less than 5 indicates slight overlap, a value from 6 to 10 indicates moderate overlap, a value from 11 to 15 corresponds to high overlap, and a value equal to or greater than 15 is considered very high overlap [20]. The overlap of primary studies within the systematic reviews included in umbrella reviews presents a unique challenge. Currently, limited guidance is available on the most effective ways to address this issue [21]. However, the following decisions were made in order to prevent double counting outcome data: If there is a complete overlap across included reviews, the review with the highest quality as decided by the JBI tool will be included in data synthesis and analysis. Also, the most recent review to be published will be used where there is complete overlap and both reviews obtain the same JBI grade. All reviews will be included when there is partial overlap, but the authors will highlight the level of duplication and discuss it as a study limitation [22].

### 2.5. Data Synthesis

The results of systematic reviews are presented as narrative summaries and in tables by the effectiveness of telerehabilitation interventions/approaches across the different outcomes. This is due to the heterogeneity of intervention, control group, outcome measures, and type of data analysis. For reviews that undertook meta-analysis, we extracted effect size, %95 CI, and heterogeneity (I^2^) for each outcome. We state effect size in this umbrella review as reported by included review authors (e.g., standardized mean differences (SMD), mean differences, and Hedges’ g). Cohen’s criteria, which state that a value between 0.2 and 0.5 shows a small effect, a value between 0.5 and 0.8 shows a medium effect, and a value greater than 0.8 shows a large effect size, were used to determine the magnitude of the effect for SMD, MD, and Hedges’ g. We used the Cochrane Handbook’s criteria to interpret the heterogeneity after extracting (I^2^), a measure of heterogeneity. I^2^ values between 0 and 50% represent unimportant and low heterogeneity, between 50% and 75% represent moderate heterogeneity, and over 75% represent considerable heterogeneity [23].

The findings of systematic reviews are presented in the “summary of evidence” table that involves the intervention, the included research synthesis, and a simple “stop light visual indicator” of intervention for each outcome. The green indicates the intervention is effective (beneficial), the orange indicates the intervention has no difference compared to the control group, and the red indicates the intervention has no effect.

## 3. Results

### 3.1. Study Inclusion

**Figure 1:**
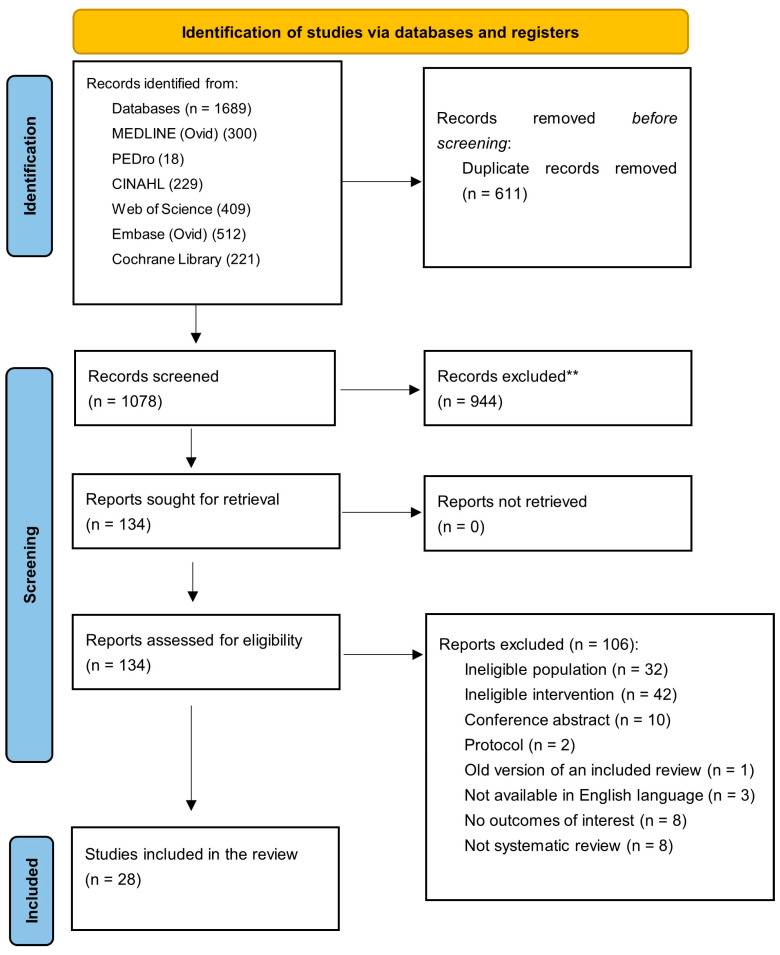
PRISMA flowchart of study selection and inclusion process. ** Records that were excluded after title and abstract screening.

Figure 1 presents the PRISMA flowchart. A total of 1689 systematic reviews were identified from searching in six databases. After removing duplicate reviews, 1078 were screened based on the titles and abstracts. Subsequently, the full texts of 134 systematic reviews were screened and assessed for eligibility. A total of 106 reviews were excluded (see Table S4), leaving 28 reviews included in this umbrella review. Table S5A,B summarizes the characteristics of the included reviews.

### 3.2. Methodological Quality Assessment of SRs

Table S6 presents the methodological quality of the 28 included SRs. Only five out of 28 systematic reviews clearly stated the review question (Q1) [12,24,25,26,27]. All but one of the included reviews had appropriate inclusion criteria (Q2) [28] and used a clear search strategy, except for five that did not report keywords and search terms (Q3) [12,29,30,31,32]. Adequate sources to search were used in all included reviews (Q4). In four systematic reviews, no critical appraisal was conducted, nor were the details of the tool that was used to assess the included studies reported [12,29,30,33], although the remaining systematic reviews appraised the studies appropriately (Q5). The critical appraisal in all but seven of the included systematic reviews was conducted by two or more reviewers (Q6). The methods used to minimize errors in data extraction were unclear in nine reviews, which failed to mention strategies to minimize bias in detail (Q7) [25,27,29,30,34,35,36,37,38]. All the included reviews used appropriate methods to combine studies except for four, in which the authors demonstrated concern about conducting a meta-analysis despite extensive heterogeneity in outcome measures, intervention content and delivery, and time since stroke; or conducted a meta-analysis with a small number of studies and small sample sizes (Q8) [28,39,40,41]. Fifteen systematic reviews failed to assess publication bias (Q9). The recommendations for policy and/or practice were provided by all but 13 of the included reviews (Q10). Three reviews failed to report the specific directives for new research (Q11) [12,25,36]. The quality of the included reviews ranged from low to high. Of 28 SRs, 1, 10, and 17 were classified as low, moderate, and high, respectively.

### 3.3. Overlapping of Primary Studies

According to the corrected covered area (CCA) formula, the result was 0.067, which shows a slight overlap of studies. This umbrella review retains all of the included reviews, since the overlap was considered to be slight [42].

### 3.4. Characteristics of the Included Studies

#### 3.4.1. Number, Types, and Date Range of the Included Studies

The 28 systematic reviews included only controlled trials, with a total of 225 RCTs, 19 pilot studies, and one longitudinal cohort with a control group study. All of the primary studies were published between 2000 and 2022.

#### 3.4.2. Country of Origin of Included Studies

The relevant primary studies were conducted in different countries, namely the USA (*n* = 39); Canada (*n* = 7); Italy (*n* = 14); the Netherlands (*n* = 12); Germany (*n* = 4); Taiwan (*n* = 5); China (*n* = 20); Spain (*n* = 16); Slovenia (*n* = 3); the UK (*n* = 8); Malaysia (*n* = 5); Belgium (*n* = 1); Brazil (*n* = 1); Australia (*n* = 4); Thailand (*n* = 1); Hong Kong (*n* = 1); Korea (*n* = 7); Austria (*n* = 1); New Zealand (*n* = 2); and Israel (*n* = 1). Thirteen SRs did not state where the primary studies were conducted.

#### 3.4.3. Participants (Total Number/Characteristics)

The total number of participants across the 28 SRs was 14,551. The number of participants in each review ranged from 45 to 1937. Age across SRs ranged from 28 to 80 years in 20 SRs, whereas eight reviews failed to state age. Gender was reported in nine SRs, two of which reported similar numbers of men and women while, in the remaining SRs, most participants were men. However, gender was not reported in 19 SRs. The stroke phase was reported in 16 SRs. One review reported all phases (acute, subacute, and chronic). Nine SRs reported both subacute and chronic phases. Three SRs reported both acute and chronic phases. Three SRs reported chronic phase only. 12 SRs failed to state the stroke phase.

#### 3.4.4. Appraisal Instruments and Rating

The appraisal instruments used for assessing the risk of bias were the PEDro checklist (8 SRs); the Cochrane Risk of Bias Tool (8 SRs); a modified McMaster critical appraisal tool (1 SR); the Australian Evidence-Based Health Care Centre tool (1 SR); Furlan Method guidelines for systematic reviews (1 SR); a mixed-method appraisal tool (MMAT) (*n* = 1); and a standard critical appraisal form (1 SR). Four systematic reviews used a mixture of instruments: the PEDro scale and the Modified Downs and Black Checklist (1 SR); the Cochrane Risk of Bias (ROB) Tool and the Modified Downs and Black Checklist (1 SR); the Cochrane ROB Tool and the PEDro scale (1 SR); and the Cochrane ROB Tool for Non-RCTs (ROBANS) and the PEDro scale (1 SR). Three systematic reviews did not report the critical appraisal tools that were used.

Nine systematic reviews revealed that the majority of the primary studies were at low risk of bias, with a high proportion of the included studies being rated as good to excellent in quality. However, two reviews did not assess the risk of bias for the primary studies. Seventeen reviews detailed risk of bias categories, including the following:

- Random sequence generation (selection bias): Reported in 10 reviews. Four reviews included studies at high or unclear risk of selection bias, while six reviews included studies at low risk of selection bias.
- Allocation concealment (selection bias): Evaluated in 13 SRs. Ten reviews included studies at low risk of bias and three reviews included studies at high or unclear risk of selection bias.
- Blinding of participants and personnel (performance bias): Reported in 12 reviews. Only two reviews included primary studies at a low risk of performance bias, while 10 reviews included studies at a high or unclear risk of performance bias. Some reviews reported that it was difficult to blind participants and personnel due to the nature of the intervention.
- Blinding outcome assessment (detection bias): Assessed in 10 reviews. Eight reviews included studies with a low risk of detection bias and two reviews reported a high or unclear risk of detection bias.
- Incomplete outcome data (attrition bias); Evaluated in six reviews. Three reviews included primary studies at low risk of attrition bias, whereas the remaining three reviews included studies at a high or unclear risk of attrition bias.
- Selective reporting (reporting bias) Evaluated in five reviews. Three reviews included primary studies at low risk of reporting bias, while two included studies with high or unclear risk of bias.
- Intention to treat (attrition bias) was assessed in three reviews. Two reviews were at high risk of bias whereas one review was at low risk of bias due to not performing an intention-to-treat analysis.
- Group similarities at the baseline: Three reviews reported whether groups were similar at the baseline. Only one review had a similar group at baseline.

#### 3.4.5. Method of Analysis

Different methods of analysis were conducted across systematic reviews, namely, narrative (descriptive) synthesis (*n* = 24); meta-analysis using fixed or random effect models based on heterogeneity (*n* = 5); and meta-analysis using only a random effect model with 95% CI (*n* = 11).

#### 3.4.6. Intervention Characteristics

Comprehensive details of the interventions are provided in (Table S5C). There was heterogeneity of telerehabilitation interventions across systematic reviews.

- Mixed telerehabilitation interventions include telephone, videoconferencing, a combination of telephone calls, in-home messaging devices, and video recording, a combination of email, an online chat program, and an online resource room (a virtual online library) established for caregivers of stroke survivors, digital video disk (DVD), educational videos, web-based chat, virtual reality system, internet-enabled computers, inertial motion sensors (IMUs) and cloud databases, games, 3D motion equipment and software to generate virtual movements, video consulting systems, text messaging, and 3D animation exercise videos) [2,12,24,26,27,28,29,30,33,34,35,37,39,40,41,43,44,45].
- Virtual reality (non-immersive VR and semi-immersive VR) and augmented reality (AR) as a standalone approach includes gaming devices to provide VR, exercises delivered through computers with monitors, an eye movement controller, a joystick, a Logitech trackpad, software platforms, a Microsoft Kinect V2 RGB-D camera, and gloves equipped with bend sensors that can be controlled remotely through the internet [25,36,46,47].
- Smartphone- or tablet-based mHealth apps [31,48,49].
- Technology-assisted self-rehabilitation [32,50].
- Robot-assisted devices, VR, and games [25].
- Videoconferencing only [38].

It was difficult to synthesize the components of each intervention that were delivered through telecommunication technology due to the heterogeneity across the primary studies. Most reviews delivered intervention at home (19 SRs) from a clinician, physical or occupational therapist, or telehealth nurse. The duration of intervention in the primary studies ranged from two weeks to one year. The frequency of intervention was daily or weekly (ranging from one to five sessions) and session duration ranged from 10 min to three hours. The control groups received either in-person (face-to-face) rehabilitation, usual care, conventional rehabilitation, other rehabilitation technology, no technology intervention, waiting list, home exercise programs (HEP), or no intervention.

#### 3.4.7. Outcomes

The systematic reviews evaluated the effects of telerehabilitation interventions on motor function (20 SRs); balance (14 SRs); gait (7 SRs); ADL (18 SRs); quality of life (8 SRs); satisfaction (8 SRs); cost (6 SRs); and adherence to therapy (4SRs).

### 3.5. Effectiveness of Telerehabilitation Interventions on Primary Outcomes

#### 3.5.1. Motor Function

A total of 20 reviews (including 94 relevant studies) evaluated and reported the impact of telerehabilitation interventions on upper and lower limb motor function (Table S7/motor function). Six high-quality reviews [24,25,33,36,47,48], three medium-quality reviews [12,31,49], and one low-quality review [29] reported significant improvement in UE measures in favour of the experimental group compared to the control group. Virtual reality was used as an intervention in five of these reviews [25,29,33,36,47], and showed positive effects on FMA-UE with medium to large effect size and considerable heterogeneity after intervention [25,36] and at follow-up [33]. Further, three reviews [31,48,49] found beneficial impacts on upper limb measures after using smartphone- or tablet-based mHealth apps, e.g., gaming apps. Fourteen SRs demonstrated insufficient effects (no statistically significant difference) for interventions that include mixed telerehabilitation approaches [28,29,30,34,40,43,45], virtual reality [26,27,33,35], augmented reality [47], technology-assisted self-rehabilitation [32] and mHealth apps [31] on upper and/or lower limb motor function compared to the control group. Heterogeneity in some of these reviews was low. Two high-quality reviews showed there was no significant effect with considerable heterogeneity of games that involve fishing, piano, and sports [25], and semi-immersive VR exercises combined with sensor gloves or music gloves [47] among all outcome measures for the upper extremity (FMA-UE, ARAT, NHPT, BBT, MAL) compared to other rehabilitation technology and home rehabilitation.

#### 3.5.2. Balance

An overview of fourteen systematic reviews, involving 62 relevant studies that assessed the effectiveness of various telerehabilitation interventions on balance for individuals with stroke is provided in (Table S7/balance). Four high-quality reviews [24,38,44,47] and two medium-quality reviews [12,31] reported significant improvements in BBS, POMA-B, and TUG in favour of the experimental group with small to large effect sizes and moderate heterogeneity. For example, Tarihoran et al. (2023) [38] found videoconferencing is effective (SMD = 1.96) in improving stroke survivors’ ability to maintain balance. On the contrary, two reviews [40,47] with medium and high quality demonstrated there were no significant beneficial effects of an intervention that involved a mix of telerehabilitation approaches [40] and augmented reality exercises at home [47] on improving balance. The remaining high- [24,34,36,37,44,45,46,47] and medium-quality [28,31,35] reviews indicated no significant differences between the intervention and control groups. In general, heterogeneity was low. Possible reasons for this include sample size, the specific technology employed, intervention components, and the characteristics of the control groups.

#### 3.5.3. Gait

Seven systematic reviews, involving 18 relevant studies, evaluated the effectiveness of various telerehabilitation interventions on gait outcomes for stroke survivors (summarised in (Table S7/gait)). The findings from the narrative synthesis across the reviews showed inconsistency regarding the impact of telerehabilitation on walking ability. Three high-quality [45,47,48] and one medium-quality [31] reviews demonstrated significant improvements in 10-MWT, 6-MWT, and POMA-G after the intervention; however, one review [36] reported similar findings after 3 months of follow-up. The interventions in these reviews include virtual reality [47], telephone-based home exercise programs, online video monitoring [45], smartphone applications [31,45,48], and tablet-based applications [48].

The remaining medium- and high-quality reviews [31,35,36,46] found no significant difference between the intervention and control groups, although both groups showed positive effects on gait measures. However, two reviews [35,47] indicated that there were no significant improvements in 6-MWT, 10-MWT, and POMA-G compared to the control group post-intervention, which involved an ankle movement program using a videoconferencing system [35] and augmented reality exercises at home [47].

#### 3.5.4. Activities of Daily Living (ADLs)

A total of 18 reviews, involving 67 relevant studies, evaluated the impact of telerehabilitation on patients with stroke ability to perform daily activities (ADLs) (Table S7/ADL). There is heterogeneity across review findings regarding the effect of telerehabilitation on ADL activities. Medium- and high-quality reviews [24,27,28,31,34,40,41,43,45] showed no significant difference between the intervention and control groups, which could be due to factors like sample size, specific technology used, or intervention components and control groups. Heterogeneity was low. Furthermore, the findings reported across studies within narrative synthesis of these reviews [35,36,40] revealed that, although both groups showed improvement post-intervention, there were no significant between-group differences. However, ten reviews with medium and high quality [25,31,35,37,38,39,41,48,49,50] found that the telerehabilitation group experienced a positive effect compared to the control group. Five of these reviews pooled data, the findings showed significant effects (effect size ≥ 0.2), and heterogeneity ranged from low to considerable. For example, Bok et al. (2023) [25] found a substantial impact with a medium effect size (0.850) from using VR compared to other rehabilitation technologies, such as games, and robot-assisted devices. Only one medium-quality review [49] demonstrated no significant benefits of BI post interventions such as rehab videos, a reminders app, or a combination of rehab videos with reminders apps, compared to the control group.

#### 3.5.5. Quality of Life (QoL)

Eight systematic reviews, involving 24 relevant studies evaluated the effectiveness of various telerehabilitation interventions on QoL measures for stroke survivors (Table S7/QoL). Two high-quality [34,43] and four medium-quality [28,31,35,49] reviews reported significant improvement in SF-12, SF-36, EQ-5D, and several domains of SS-QoL for those in the telerehabilitation group compared to the control group. This finding is based on one primary study in each review. Only one medium-quality review [49] showed no significant benefit from using therapy apps on QoL compared to conventional rehabilitation. Further, three high-quality [34,36,43] and three medium-quality reviews [28,31,40] mentioned that there was no significant difference between the intervention and control groups. This could be due to the telerehabilitation intervention content, technology used, and the characteristics of the control group.

### 3.6. Effectiveness of Telerehabilitation Interventions on Feasibility Outcomes

#### 3.6.1. Adherence to Treatment

Four systematic reviews, involving 13 relevant studies, evaluated the effectiveness of various telerehabilitation interventions on adherence to treatment for stroke survivors (Table S7/adherence to treatment). Adherence to therapy was assessed by the rate of participant dropout in one medium-quality review [35], while others did not mention how adherence was assessed. Two medium- [35,49] and one high-quality review [46] showed positive findings, and there was a good adherence to the intervention compared to the control. For example, Deshmukh and Madhavan (2023) [35] reported that the participant dropout rate was low (only 13 out of 248 participants), indicating excellent adherence to the therapy. However, Schroder et al. (2018) [46] reported that 50% of participants adhered to daily walking and engaged in 150 min of moderate physical activity per week, while 14% included core-stability exercises five days a week in their routines.

#### 3.6.2. Participant Satisfaction with the Intervention

Eight systematic reviews, encompassing 23 relevant studies, assessed the impact of telerehabilitation interventions on patient satisfaction (Table S7/participant satisfaction). Three reviews with high [43], medium [39] and low [29] quality indicated that patients were generally satisfied with telerehabilitation, with higher satisfaction rates in the intervention group compared to the control group. One medium- [28] and one high-quality [36] review presented positive outcomes based on one primary study, showing that Tele VR had higher or equivalent scores compared to in-hospital VR. Three high-quality [2,34,36] and two medium-quality reviews [28,40] found no significant differences between the intervention and control groups.

#### 3.6.3. Cost-Effectiveness

The cost of telerehabilitation interventions was examined in six systematic reviews, including two relevant studies (Table S7/cost). None of the meta-analyses investigated the cost-effectiveness of these interventions. The narrative synthesis from two high-quality [36,46] and four medium-quality reviews [28,30,35,40] indicated that virtual reality-based telerehabilitation is about USD 654 cheaper than in-person rehabilitation, based on the same primary study. However, one high-quality review [46] presented conflicting findings regarding the costs of VR equipment, based on another primary study. Specific equipment is needed for telerehabilitation, which might be costly.

Summary of Evidence: the summary of the evidence is presented in Table 1.

## 4. Discussion

This umbrella review found good acceptability and engagement with telerehabilitation interventions in the subacute and chronic phases after stroke. These interventions were more likely to benefit upper limb motor function, balance, and activities of daily living (ADLs). However, interventions targeting lower limb motor function, gait, and quality of life (QoL) appeared generally less effective, likely due to the smaller number of studies addressing these outcomes. This review highlights the potential of telerehabilitation interventions but the need for further innovation to improve functional outcomes, as well as the development and evaluation of cost-effective approaches.

Overall, for primary outcomes related to upper and lower limb motor function, balance, gait, activities of daily living, and quality of life, our findings highlight moderate- to high-quality evidence showing either a significant effect or no significant difference between telerehabilitation and other interventions. These findings are consistent with a recent review conducted by Dias et al. (2021), which suggests that exercise through telerehabilitation may be a more effective option for treating pain, enhancing physical function, and improving the quality of life in individuals with physical disabilities [51]. Furthermore, an umbrella review by Suso-Marti et al. (2021) showed that telerehabilitation positively affects physical functions in patients with neurological conditions compared to those with musculoskeletal and cardiovascular conditions [11]. This may be because patients with neurological disorders often require high doses and continuous recovery treatment to achieve functional improvements [8], which is difficult to provide through in-person interventions due to short hospitalization and a lack of staff members [52]. Furthermore, during the COVID-19 pandemic, most rehabilitation services and consultations were either postponed or delivered remotely through telerehabilitation, particularly for conditions that result in long-term impairments [53,54]. Therefore, integrating telerehabilitation, telemedicine, and tele-assessment into routine practice could enhance the quality of care for individuals with chronic illnesses [53,54].

We found that motor function, specifically, for the upper limb, is the most studied outcome domain across reviews. This emphasis could be due to approximately 50–80% of stroke survivors experiencing upper limb impairment, with about 50% of patients continuing to experience these deficits in the chronic phase, six months post-stroke [55]. Furthermore, recovery of upper limb motor skills can significantly enhance quality of life, making it a central focus in telerehabilitation interventions [40]. However, most telerehabilitation interventions showed positive or similar impacts (with no significant differences) in improving upper limb motor function. This indicates uncertainty about the effects of various telerehabilitation interventions, which could be attributed to small sample sizes in the RCTs or variations in intervention components, outcome measures, and technology used. The limitation of reporting lower motor function could be due to concerns about fall risks, which may lead to physical exercise programs for motor recovery of the lower limb being delivered in person [56].

Furthermore, the findings of this umbrella review suggest that telerehabilitation could have a significant improvement or similar effect on balance and gait for stroke survivors, despite the presence of heterogeneity across studies. These findings are in agreement with a recent review that found telerehabilitation appears to be an alternative to traditional rehabilitation techniques in terms of enhancing gait and balance in multiple sclerosis [57]. However, findings from the scoping review reported that there was not enough evidence to determine if videoconferencing-based telerehabilitation improved the balance and gait of individuals with Parkinson’s disease (PD) [58]. Therefore, future studies are needed, as there is currently insufficient evidence, especially on lower limb motor function and walking ability.

Activities of daily living (ADLs) are considered the second outcome most studied after upper limb motor function across the included reviews. This could be due to certain ADLs, such as walking, cooking, reading, engaging in leisure activities, performing housework, and managing finances, contributing to enhanced physical, cognitive, and executive functioning [59]. The findings were generally positive, as the improvement was observed across various approaches, including videoconferencing, telephone calls, messaging, virtual reality, 3D animation videos, and mHealth apps. Regarding quality of life, our findings from a few reviews are consistent with those of a recent review, indicating that telerehabilitation has a significant and positive impact on the quality of life for patients with neurological conditions, including stroke, Parkinson’s disease, and multiple sclerosis [60]. This improvement is attributed to patients not having to travel to rehabilitation facilities and being able to stay at home with family members and caregivers [60].

Regarding the feasibility outcomes, this umbrella review identifies excellent adherence and satisfaction with telerehabilitation interventions including virtual reality, videoconferencing, 3D motion equipment, telephone calls, and game-based therapy through videoconferencing. This could be due to the fact that patients with neurological conditions may find that interactive technology, which provides them with real-time feedback on how well they are doing a task, is enjoyable, motivating, and more likely to improve adherence to the treatment plan [57]. Further, we found, from a few systematic reviews, that telerehabilitation, e.g., VR, is cheaper than in-person rehabilitation. These findings align with a recent review, which concluded that telerehabilitation offers comparable clinical and cost-effectiveness outcomes to traditional rehabilitation for people with neurological and cardiological conditions, while generally being less expensive [54]. However, further studies are recommended to evaluate its economic impact on the healthcare system [61]. Overall, as the field is still emerging, the evidence remains insufficient to draw definitive conclusions about feasibility and cost-effectiveness outcomes.

Patients with stroke in the subacute or chronic phase were included in the majority of reviews under this umbrella review. Since all functional outcomes recover spontaneously in the early stages post-stroke due to brain plasticity, considerable improvements are often observed during the subacute phase [62]. However, in the chronic phase (after six months), the function of brain plasticity tends to plateau [62]. Therefore, telerehabilitation approaches, such as virtual reality, have been shown to induce significant changes in brain plasticity, alongside functional recovery from the acute to chronic phases of stroke [63].

Simple and complex telerehabilitation interventions such as telephone calls, videoconferencing, smartphone- or tablet-based mobile health applications, messaging, VR, robot-assisted devices, and 3D animation videos, either alone or in combination with other interventions, were used across reviews. Videoconferencing, delivered via desktop computer, laptop, or iPad, is the most common approach used either standalone or combined with other technology or conventional therapy. Stephenson’s review emphasized that one advantage of videoconferencing is its ability to facilitate synchronous telerehabilitation, where patients can engage with clinicians in real time and receive immediate feedback on their rehabilitation progress [6]. Also, it seems that virtual reality might be a superior telerehabilitation approach for enhancing upper limb motor function and ADL in people with stroke. This is in agreement with the findings of the umbrella review conducted by Voinescu [64], which identified the potential benefits of VR for enhancing balance, upper extremity motor function, and mobility in patients with stroke, ambulation function of children with cerebral palsy, and upper extremity function for individuals with acquired brain injury. This could be due to VR exercise programs that can provide patients with stroke with intensive, repetitive, and task-specific training [65]. They also incorporate key concepts that promote brain plasticity, leading to improvements in motor function after a stroke [63,65].

Despite the evidence and specific features of various telerehabilitation interventions, there is still a deficiency in the integration of these interventions into healthcare systems. It is important to consider the patient’s and healthcare provider’s experiences, perspectives, and preferences, to identify barriers and facilitators when planning and providing telerehabilitation interventions [66]. Some may doubt, or believe face-to-face rehabilitation is a better treatment model than telerehabilitation. For this reason, it is essential to conduct a mixed-methods study to explore the feasibility and acceptability of telerehabilitation interventions for stroke survivors and healthcare providers.

Recommendations for future research:

Our findings highlight a lack of existing studies focusing on the effects of telerehabilitation interventions on functional outcomes in Middle Eastern countries. Also, there is insufficient evidence about the effects of various telerehabilitation interventions on lower limb motor function, gait, quality of life, treatment adherence, participant satisfaction, and cost-effectiveness. This umbrella review recommends that future reviews avoid grouping diverse interventions under the broad term “telerehabilitation”. Instead, they should consider the specific components of the interventions, along with the details of the approaches, technologies, and dosages used. Additionally, further high-quality randomized controlled trials (RCTs) are needed to investigate whether telerehabilitation interventions can be used as an alternative or an addition to face-to-face conventional therapy (hybrid program).

Strengths and limitations:

This umbrella review investigated the effectiveness of various telerehabilitation interventions on primary and feasibility outcomes after stroke. We used a robust methodology, including developing an a priori protocol, extensive database and reference list searches, and using the JBI to assess the quality of the included systematic reviews. This umbrella included separate data synthesis for each outcome. This approach enabled us to perform an in-depth data synthesis to determine the most effective outcomes for telerehabilitation interventions.

There were several limitations to this umbrella review. Firstly, the biggest challenge in the synthesis of interventions was variations in intervention components, delivery, and technology used. We synthesised the interventions based on the review levels, as some reviews included mixed telerehabilitation interventions (synchronous and asynchronous interventions), while others specified their intervention to mobile health applications, VR- and technology-assisted self-rehabilitation. Also, some reviews provided vague descriptions of the intervention components and technologies used. Additionally, it was common to pool all interventions described as telerehabilitation without considering the specific technologies involved. This made it challenging to determine the effect size of each technology when various technologies were combined. It is important that the telerehabilitation interventions which utilize telecommunication technology need to be clearly defined, and analyses should be pooled based on the specific technology. This umbrella review included systematic reviews with overlapping primary studies, which can lead to double counting of evidence. Further, restrictions on the language used in our searches might have caused relevant citations to be overlooked. The systematic reviews included in this study had their limitations, including that the findings cannot be widely generalized due to the small sample size [2,12,24,28,29,34,40,41,46,48,49,50], the small number of high-quality studies [2,12,24,26,33,34,36,37,40,41,46,48,49,50], poor reporting, that a lack of clarity from study authors made it unclear which situations were at risk of bias [25,27,34], the heterogeneity in the content of the intervention, telerehabilitation approach, and outcome measures used [2,12,24,26,28,33,39,41,43,46,47], the unknown long-term follow-up effects of the intervention [35,40,44,50], and the inability to blind the participants due to the nature of the intervention [35,38,44,46,49].

## Figures and Tables

**Table 1:** Summary of evidence.

Telerehabilitation Interventions	SRs	Outcomes												
		Motor Function		Balance		Gait		ADL		QoL		Adherence	Participant Satisfaction	Cost Effectiveness
		Post Intervention	Follow Up	Post Intervention	Follow Up	Post Intervention	Follow Up	Post Intervention	Follow Up	Post Intervention	Follow Up	Post Intervention	Post Intervention	Post Intervention
Virtual reality	Bok et al. (2023) [25]	UL												
	Coupar et al. (2012) [33]	UL												
	Chen et al. (2015) [40]													
	Deshmukh and Madhavan (2023) [35]	UL/LL												

	Hao et al. (2023) [36]	UL												
	Johansson and Wild (2011) [29]	UL												
	Lazem et al. (2023) [47]	UL												
	Nascimento et al. (2022) [27]	UL												
	Schroder et al. (2018) [46]													
	Sarfo et al. (2018) [30]													
	Tchero et al. (2018) [28]													
	Toh et al. (2022) [26]	UL	UL/LL											
VR exercises combined with sensor gloves or music gloves	Lazem et al. (2023) [47]	No effect UL												
Robot-assisted devices	Bok et al. (2023) [25]	UL												
Games	Bok et al. (2023) [25]	No effect/UL												
	Ostrowaska et al. (2021) [12]	UL												
	Sharififar et al. (2023) [39]													
Augmented reality	Lazem et al. (2023) [47]	LL		No effect		No effect								

Technology-assisted self-rehabilitation	Everard et al. (2021) [32]	UL												
	Hwang et al. (2021) [50]													
Mixed telerehabilitation interventions	Appleby et al. (2019) [43]	UL												
	Alayat et al. (2022) [44]													
	Chen et al. (2015) [40]	UL		No effect										
	Johansson and Wild (2011) [29]													
	Laver et al. (2020) [34]	UL												
	Lombardo and Islam (2023) [2]													
	Rintala et al. (2019) [45]	UL/LL												
	Sarfo et al. (2018) [30]	UL/LL												
	Saragih et al. (2022) [37]													
	Sharififar et al. (2023) [39]													
	Su et al. (2023) [24]	UL												
	Tchero et al. (2018) [28]	UL												

	Qin et al. (2022) [41]													
Videoconferencing only	Deshmukh and Madhavan (2023) [35]					No effect								
	Tarihoran et al. (2023) [38]													
Smartphone- or tablet-based mHealth apps	Rintala et al. (2023) [31]	UL												
		LL												
	Szeto et al. (2023) [49]	UL												
										No effect				
	Zhou et al. (2018) [48]	UL												

The colour coding in the table pertains to the following: The green indicates the intervention is effective (beneficial), the orange indicates the intervention has no difference compared to the control group, the red indicates the intervention has no effect, and the white indicates not reported.

## Data Availability

Data are contained within this article or the Supplementary Materials.

## Supplementary Materials

The following supporting information can be downloaded at: https://www.mdpi.com/article/10.3390/jcm14010050/s1, Tables S1 and S2: Search detail, Table S3: Overlapping of primary studies, Table S4: Excluded studies, Table S5: Characteristics of the included reviews, Table S6: Methodological quality of SRs, Table S7: findings of the umbrella review.
