# Supplementary material for: Telerehabilitation and Its Impact Following Stroke: An Umbrella Review of Systematic Reviews
Source: J Clin Med. 2024 Dec 26;14(1):50. doi: 10.3390/jcm14010050 (PMC11721391; doi:10.3390/jcm14010050)
Supplement: Supplementary file 1 [file jcm-14-00050-s001.zip › Table S1 and S2 Search detail.pdf]

Table S1: The search terms

| Keywords | Alternatives |
|---|---|
| Population (stroke) | “Stroke” OR “brain vascular accident” OR “cerebrovascular accident” OR “cerebral stroke” OR “cerebrovascular stroke” OR “acute stroke” OR “chronic stroke” OR “hemiplegia” OR “hemiparesis” OR ‘’CVA’’ OR “hemorrhagic stroke” OR “ischemic stroke” OR “post-stroke” OR “after stroke”. |
| Intervention<br>(Telerehabilitation) | "telerehabilitation” OR "Tele-rehabilitation" OR "Home rehabilitation" OR "home therapy" OR "Remote Rehabilitation" OR "Virtual Rehabilitation" OR "tele exercise" OR” Virtual therapy” OR” Videoconferencing” OR “m-health” OR “mobile health” OR “e-health” OR “telehealth” OR “home based telerehabilitation” OR “telecare” OR “telemedicine” OR “electronic health” OR “tele-stroke” OR Home* OR distance* OR remote*. |
| Outcomes<br>(Motor function, balance, gait, activities of daily living, and quality of life) | "mobility" OR "motor function" OR “physical function” OR "motor activity" OR "balance " OR "stability" OR "gait" OR "walking" OR "upper limb function*" OR “upper extremity” OR "arm" OR "hand" OR “lower limb function*” OR “lower extremity” OR "activit* of daily living" OR "daily living activit*" OR "ADL" OR "quality of life" OR "QoL" OR “cost effect*” OR “cost” OR “patient satisfaction” OR “ Patient adherence”. |
| Study design | quantitative systematic reviews :“systematic review*” or “evidence synthesis” or “Clinical effectiveness review” or “meta-analysis” or “mixed methods review”). |

Table S2:

| Date | Database | Keywords | Number |
|---|---|---|---|
| 08/12/2023 | (Ovid)Medline All 1946 to December 07,2023 | 1. Stroke/ 135749<br>2. Stroke .ab,ti. 309682<br>3. cerebrovascular accident. ab,ti. 5086<br>4. brain vascular accident. ab,ti. 6<br>5. cerebral stroke. ab,ti. 1686<br>6. acute stroke. ab,ti. 18543<br>7. chronic stroke. ab,ti. 4454<br>8. ischemic stroke. ab,ti. 64463<br>9. Ischemic Stroke/ 10822<br>10. Hemorrhagic stroke/ 595<br>11. hemorrhagic stroke. ab,ti. 6446<br>12. CVA. ab,ti. 3508<br>13. "hemiplegi*". ab,ti. 13021<br>14. "hemipare*". ab,ti. 13748<br>15. post-stroke. ab,ti. 14626<br>16. after stroke. ab,ti. 24043<br>17. 1 or 2 or 3 or 4 or 5 or 6 or 7 or 8 or 9 or 10 or 11 or 12 or 13 or 14 or 16 350987<br>18. Telerehabilitation/ 1065<br>19. telerehabilitation. ab,ti. 1732<br>20. tele rehabilitation. ab,ti. 287<br>21. "tele*". ab,ti. 202705<br>22. Telemedicine/ 38441<br>23. remote rehabilitation. ab,ti. 100<br>24. virtual rehabilitation. ab,ti. 162<br>25. home rehabilitation. ab,ti. 540<br>26. "home therap*". ab,ti. 886<br>27. home based telerehabilitation. ab,ti. 95<br>28. tele exercise. ab,ti. 43<br>29. m-health. ab,ti. 600<br>30. mobile health. ab,ti. 6941<br>31. e-health. ab,ti. 3065<br>32. electronic health. ab,ti. 30603<br>33. "mobile app*". ab,ti. 9064<br>34. Videoconferencing. ab,ti. 2812<br>35. (home* or distance* or remote*). ab,ti. 1079986 | 300 |

36. 18 or 19 or 20 or 21 or 22 or 23 or 24 or 25 or 26 or 27 or 28 or 29 or 30 or 31 or 32 or 33 or 34 or 35 1302990
37. mobility. ab,ti. 177643
38. “motor function*”. ab,ti. 36494
39. “physical function*”. ab,ti. 33719
40. motor activity. ab,ti. 15943
41. balance. ab,ti. 277712
42. stability. ab,ti. 581124
43. gait. ab,ti. 65541
44. walking. ab,ti. 91354
45. “upper limb function*”. ab,ti. 2076
46. upper extremity. ab,ti. 25430
47. “lower limb function*”. ab,ti. 766
48. lower extremity. ab,ti. 44858
49. “activit* of daily living”. ab,ti. 36869
50. “daily living activit*”. ab,ti. 1793
51. ADL. ab,ti. 13394
52. quality of life. ab,ti. 384539
53. QoL. ab,ti. 53853
54. patient satisfaction. ab,ti. 46597
55. patient adherence. ab,ti. 4751
56. “cost effect*”. ab,ti. 179951
57. “cost”. ab,ti. 571195
58. 37 or 38 or 39 or 40 or 41 or 42 or 43 or 44 or 45 or 46 or 47 or 48 or 49 or 50 or 51 or 52 or 53 or 54 or 55 or 56 or 57 2166729
59. “Systematic review”/ 246662
60. (“systematic review*” or “evidence synthesis” or “Clinical effectiveness review” or “meta-analysis” or “mixed methods review”). ab,ti. 425231
61. Systematic review.pt. 246662
62. 59 or 60 or 61 450722
63. 17 and 36 and 58 and 62 300

| | | | |
|---|---|---|---|
| | | Method: Systematic review | |
| 13/12/2023 | CINAHL | Searching on abstract and title:<br>**S1** TI ( (MH "Stroke") OR (MH "Ischemic Stroke") OR (MH "Hemorrhagic Stroke") ) OR AB ( (MH "Stroke") OR (MH "Ischemic Stroke") OR (MH "Hemorrhagic Stroke") ) OR TI ( "cerebrovascular accident" or cva ) OR AB ( "cerebrovascular accident" or cva ) OR TI ( hemiplegia or hemiparesis or hemiparetic or hemiplegic ) OR AB ( hemiplegia or hemiparesis or hemiparetic or hemiplegic ) **(86,118)**<br>**S2** TI ( telerehabilitation or tele-rehabilitation or telemedicine or "virtual rehabilitation" or "remote rehabilitation" or "tele exercise" or "tele*" ) OR AB ( telerehabilitation or tele-rehabilitation or telemedicine or "virtual rehabilitation" or "remote rehabilitation" or "tele exercise" or "tele*") OR TI ( "home rehabilitation" or "home therap*" or "home based telerehabilitation" or home or "remote*" or "distance*") OR AB ( "home rehabilitation" or "home therap*" or "home based telerehabilitation" or home or "remote*" or "distance*" ) OR TI ( mhealth or mobile health or m-health or e-health or electronic health or "mobile app* or Videoconferencing" ) OR AB ( mhealth or mobile health or m-health or e-health or electronic health or "mobile app* or Videoconferencing ) **(340,790)**<br>**S3** TI ( "motor function*" or "physical function*" or mobility or motor activity or "upper limb function*" or "upper extremity" or "lower limb function*" or "lower extremity" ) OR AB ( "motor function*" or "physical function*" or mobility or motor activity or "upper limb function*" or "upper extremity" or "lower limb function*" or "lower extremity" ) OR TI ( balance or stability ) OR AB ( balance or stability ) OR TI ( gait or walking ) OR AB ( gait or walking ) OR TI ( "activit* of daily living" or ADL ) OR AB ( "activit* of daily living" or ADL ) OR TI ( “quality of life” or QoL or satisfaction or adherence or cost ) OR AB ( quality of life or QoL or satisfaction or adherence or cost ) **(699,301)**<br>**S4** TI ( review* or systematic review or meta-analysis or evidence synthesis ) OR AB ( review* or systematic review or meta-analysis or evidence synthesis ) (826,260)<br>**S5** (TI ( review* or systematic review or meta-analysis or evidence synthesis ) OR AB ( review* or systematic review or meta-analysis or evidence synthesis )) AND (S1 AND S2 AND S3 AND S4)<br>**(229)** | 229 |

| | | | |
|---|---|---|---|
| | | **Expanders** - Apply equivalent subjects<br>**Narrow by Language:** - english<br>**Search modes** - Boolean/Phrase | |
| 13/12/2023 | Web of Science | Searching on Topic:<br>“Stroke” OR "cerebrovascular accident*" OR “cerebral stroke” OR “acute stroke” OR “chronic stroke” OR “hemiplegi*” OR “hemipare*” OR ‘’CVA’’ OR “haemorrhagic stroke” OR “Ischemic stroke” OR “post-stroke” OR “after stroke”<br>AND<br>"telerehabilitation” OR "Tele-rehabilitation" OR "Home rehabilitation" OR "home therapy" OR "Remote Rehabilitation" OR "Virtual Rehabilitation" OR "tele exercise" OR "tele*" OR ”Virtual therapy” OR” Videoconferencing” OR “m-health” OR “mobile health” OR “e-health” OR “telehealth” OR “home based telerehabilitation” OR “telemedicine” OR “electronic health” OR “tele-stroke” OR "mobile app*" OR home* OR distance* OR remote*<br>AND<br>"mobility" OR "motor function*" OR "motor activity" OR "balance " OR "stability" OR "gait" OR "walking" OR "upper limb function*" OR “upper extremity” OR “lower limb function*” OR “lower extremity” OR "activit* of daily living" OR "daily living activit*" OR "ADL" OR "quality of life" OR "QoL" OR “cost effect*” OR “cost” OR "satisfaction” OR “adherence”<br>AND<br>systematic review* OR evidence synthesis OR meta analysis OR meta-analysis OR mixed methods review. (Quotation marks)<br><br>**Limiters:** English Language<br>**Document type:** review article | 409 |
| 10/12/2023 | Embase (Ovid) 1974 to 2023 December 08 | 1. cerebrovascular accident/ 295654<br>2. Stroke .ab,ti. 496025<br>3. cerebrovascular accident. ab,ti. 8716<br>4. brain vascular accident. ab,ti. 12<br>5. cerebral stroke. ab,ti. 2569<br>6. acute stroke. ab,ti. 34363<br>7. chronic stroke. ab,ti. 5973<br>8. Ischemic Stroke/ 20716<br>9. ischemic stroke. ab,ti 109057<br>10. hemorrhagic stroke. ab,ti. 11161<br>11. CVA. ab,ti. 8345<br>12. “hemiplegi*”. ab,ti. 17391<br>13. Hemiplegia/ 21163 | 512 |

14. “hemipare*”. ab,ti. 21438
15. Hemiparesis/ 28464
16. post-stroke. ab,ti. 26114
17. after stroke. ab,ti. 37760
18. 1 or 2 or 3 or 4 or 5 or 6 or 7 or 8 or 9 or 10 or 11 or 12 or 13 or 14 or 16 or 17 627255
19. Telerehabilitation/ 2769
20. telerehabilitation. ab,ti. 2043
21. tele rehabilitation. ab,ti. 434
22. “tele*”. ab,ti. 258953
23. Telemedicine/ 45913
24. remote rehabilitation. ab,ti. 126
25. virtual rehabilitation. ab,ti. 233
26. home rehabilitation. ab,ti. 773
27. “home therap*”. ab,ti. 1435
28. home based telerehabilitation. ab,ti. 111
29. tele exercise. ab,ti. 43
30. m-health. ab,ti. 835
31. mobile health. ab,ti. 7085
32. e-health. ab,ti. 4223
33. electronic health. ab,ti. 45801
34. “mobile app*”. ab,ti. 11651
35. Videoconferencing. ab,ti. 3522
36. (home* or remote* or distance*). ab,ti. 1364394
37. 19 or 20 or 21 or 22 or 23 or 24 or 25 or 26 or 27 or 28 or 29 or 30 or 31 or 32 or 33 or 34 or 35 or 36 1649912
38. mobility. ab,ti. 207445
39. “motor function*”. ab,ti. 52272
40. “physical function*”. ab,ti. 52117
41. motor activity. ab,ti. 19937
42. balance. ab,ti. 349285
43. stability. ab,ti. 647107
44. gait. ab,ti. 96597
45. walking. ab,ti. 126971
46. “upper limb function*”. ab,ti. 3127
47. upper extremity. ab,ti. 34425

| | | | |
|---|---|---|---|
| | | 48. "lower limb function*". ab,ti. 1069<br>49. lower extremity. ab,ti. 64403<br>50. "activit* of daily living". ab,ti. 51921<br>51. "daily living activit*". ab,ti. 2845<br>52. ADL. ab,ti. 21676<br>53. quality of life. ab,ti. 602611<br>54. QoL. ab,ti. 100109<br>55. patient satisfaction. ab,ti. 67988<br>56. patient adherence. ab,ti. 7890<br>57. "cost effect*". ab,ti. 242831<br>58. "cost". ab,ti. 757752<br>59. 38 or 39 or 40 or 41 or 42 or 43 or 44 or 45 or 46 or 47 or 48 or 49 or 50 or 51 or 52 or 53 or 54 or 55 or 56 or 57 or 58 2816578<br>60. "Systematic review"/ 443359<br>61. ("systematic review*" or "evidence synthesis" or "Clinical effectiveness review" or "meta-analysis" or "mixed methods review"). ab,ti. 526843<br>62. 60 or 61 655170<br>63. 18 and 37 and 59 and 62 513 | |
| 08/12/2023 | Cochrane Library | #1 MeSH descriptor: [Stroke] this term only 13665<br>#2 "hemiplegia" OR "hemiparesis" in Cochrane Reviews 133<br>#3 "haemorrhagic stroke" OR "ischemic stroke" in Cochrane Reviews 288<br>#4 "post-stroke" OR "after stroke" in Cochrane Reviews208<br>#5 #1 OR #2 OR #3 OR #4 in Cochrane Reviews 383<br>#6 MeSH descriptor: [Telerehabilitation] this term only 291<br>#7 "mhealth" OR "mobile health" OR "ehealth" OR "electronic health" in Cochrane Reviews 183<br>#8 "telehealth" OR "telecare" OR "telemedicine" OR "tele*" in Cochrane Reviews 3052<br>#9 "home based telerehabilitation" OR "home rehabilitation" in Cochrane Reviews 29<br>#10 home* or distance* or remote* in Cochrane Reviews 3550<br>#11 #6 OR #7 OR #8 OR #9 OR #10 in Cochrane Reviews 5060<br>#12 "mobility" OR "motor function" OR "motor activity" OR "upper limb" OR "upper extremity" OR "lower limb" OR "lower extremity" in Cochrane Reviews 1505<br>#13 "balance" OR "stability" in Cochrane Reviews 2295 | 221 |

#14 "gait" OR "walking" in Cochrane Reviews
956
#15 "activities of daily living" OR "daily living activities" OR "ADL" in Cochrane Reviews 832
#16 "quality of life" OR "QoL" in Cochrane Reviews 5072
#17 "patient adherence" in Cochrane Reviews
105
#18 "patient satisfaction" in Cochrane Reviews 860
#19 "cost effect" OR "cost" in Cochrane Reviews 4748
#20 #12 OR #13 OR #14 OR #15 OR #16 OR #17 OR #18 OR #19 in Cochrane Reviews 7530
#21 systematic review* OR evidence synthesis OR meta analysis OR meta-analysis OR mixed methods review. in Cochrane Reviews
9170
#22 #5 AND #11 AND #20 AND #21 in Cochrane Reviews
221
