# Supplementary material for: Telerehabilitation and Its Impact Following Stroke: An Umbrella Review of Systematic Reviews
Source: J Clin Med. 2024 Dec 26;14(1):50. doi: 10.3390/jcm14010050 (PMC11721391; doi:10.3390/jcm14010050)
Supplement: Supplementary file 1 [file jcm-14-00050-s001.zip › Table S4 Excluded studies.pdf]

Table S4 : Excluded studies and the reason for exclusion (n= 106)

	Study	Reason for exclusion
1	Moreno-Ligero M, Lucena-Anton D, Salazar A, Failde I, Moral-Munoz JA. mHealth Impact on Gait and Dynamic Balance Outcomes in Neurorehabilitation: Systematic Review and Meta-analysis. Journal of Medical Systems. 2023;47(1):1-19.	Ineligible population: a mixture of neurological disorders such as stroke, Parkinson's disease, multiple sclerosis, and neurological conditions.
2	Gelaw AY, Janakiraman B, Gebremeskel BF, Ravichandran H. Effectiveness of Home-based rehabilitation in improving physical function of persons with Stroke and other physical disability: A systematic review of randomized controlled trials. J Stroke Cerebrovasc Dis. 2020;29(6):104800.	Ineligible population: A mixture of medical conditions such as stroke, diabetes mellitus, Chronic Obstructive Pulmonary Disease, Parkinsons disease, partial or total arthroplasty, low back pain, hip fracture, osteoarthritis, and cerebral palsy children who developed a physical disability or physical functional limitation.
3	Da-Silva RH, Moore SA, Price CI. Self-directed therapy programmes for arm rehabilitation after stroke: a systematic review. CLINICAL REHABILITATION. 2018;32(8):1022-36.	Ineligible population: Populations with mixed impairment aetiology.
4	Brown R, Coombes J, Rodriguez K, Hickman I, Keating S. Effectiveness of exercise via telehealth for chronic disease: a systematic review and meta-analysis of exercise interventions delivered via videoconferencing. BRITISH JOURNAL OF SPORTS MEDICINE. 2022;56(18):1042-+.	Ineligible population: participants with chronic diseases such as pulmonary conditions, cardiac diseases, metabolic disorders, neurological disorders, cancer, and musculoskeletal conditions.

5	Cugusi L, Prosperini L, Mura G. Exergaming for Quality of Life in Persons Living with Chronic Diseases: A Systematic Review and Meta-analysis. PM R. 2021;13(7):756-80.	Ineligible population: people neurological with disorders, rheumatologic diseases, cardiorespiratory and chronic metabolic conditions.
6	Moral-Munoz JA, Zhang W, Cobo MJ, Herrera-Viedma E, Kaber DB. Smartphone-based systems for physical rehabilitation applications: A systematic review. Assistive technology : the official journal of RESNA. 2021;33(4):223-36.	Ineligible population: A mixture of medical conditions such as stroke, cardiac disease, Parkinson's disease, respiratory disease, and joint/limb rehabilitation
7	Laver K, Walker M, Ward N. Telerehabilitation for Stroke is Here to Stay. But at What Cost? Neurorehabilitation & Neural Repair. 2022;36(6):331-4.	Not systematic review
8	Truijen S, Abdullahi A, Bijsterbosch D, van Zoest E, Conijn M, Wang Y, et al. Effect of home-based virtual reality training and telerehabilitation on balance in individuals with Parkinson disease, multiple sclerosis, and stroke: a systematic review and meta-analysis. Neurol Sci. 2022;43(5):2995-3006.	Ineligible population: patients with Parkinson's disease, Multiple sclerosis, or stroke.
9	Legg L, Langhorne P. Rehabilitation therapy services for stroke patients living at home: systematic review of randomised trials. Lancet. 2004;363(9406):352-6.	No outcomes of interest.<br><br>Also, The review focuses on rehabilitation therapy services without the use of a telerehabilitation approach.

10	Velayati F, Ayatollahi H, Hemmat M. A Systematic Review of the Effectiveness of Telerehabilitation Interventions for Therapeutic Purposes in the Elderly. Methods of information in medicine. 2020;59(2-03):104-9.	Ineligible population: a mixture of health conditions including total knee replacement, chronic stroke, stroke-related disorders, chronic obstructive pulmonary disease (COPD) and the comorbidity of COPD and chronic heart failure (CHF).
11	Zhou L, Parmanto B. Reaching People With Disabilities in Underserved Areas Through Digital Interventions: Systematic Review. Journal of medical Internet research. 2019;21(10):e12981.z	Ineligible population: patients with disabilities (eg, developmental, cognitive, vision, intellectual, and mobility impairments, as well as impairments caused by problems such as TBI, stroke, autism, spinal cord injury, CP, MS, and spina bifida).
12	Del Pino R, Diez-Cirarda M, Ustarroz-Aguirre I, Gonzalez-Larragan S, Caprino M, Busnatu S, et al. Costs and effects of telerehabilitation in neurological and cardiological diseases: A systematic review. Frontiers in medicine. 2022;9(101648047):832229.	Ineligible population: patients with neurological and cardiological diseases.
13	Maresca G, Maggio MG, De Luca R, Manuli A, Tonin P, Pignolo L, Calabro RS. Tele-Neuro-Rehabilitation in Italy: State of the Art and Future Perspectives. Frontiers in neurology. 2020;11:563375.	Ineligible population: neurological and neurodegenerative patients, including neurodevelopmental disorders.
14	Leon-Salas B, Gonzalez-Hernandez Y, Infante-Ventura D, de Armas-Castellano A, Garcia-Garcia J, Garcia-Hernandez M, et al. Telemedicine for neurological diseases: A systematic review and meta-analysis.	Ineligible population: a mixture of neurological diseases such as stroke, Parkinson's disease, multiple sclerosis, epilepsy, dementia, spina befida, migraine, cerebral palsy, and brain damage.

	European journal of neurology. 2023;30(1):241-54.	
15	Palacios-Navarro G, Hogan N. Head-Mounted Display-Based Therapies for Adults Post-Stroke: A Systematic Review and Meta-Analysis. Sensors (Basel, Switzerland). 2021;21(4).	Ineligible intervention: Non telerehabilitation intervention.
16	Hailey D, Roine R, Ohinmaa A, Dennett L. Evidence of benefit from telerehabilitation in routine care: a systematic review. J Telemed Telecare. 2011;17(6):281-7.	Ineligible population: patients with any disabilities
17	Saunders D, Sanderson M, Hayes S, Johnson L, Kramer S, Carter D, et al. Physical fitness training for stroke patients. Cochrane Database of Systematic Reviews. 2020(3).	Ineligible intervention: Non telerehabilitation intervention
18	Thijs L, Voets E, Denissen S, Mehrholz J, Elsner B, Lemmens R, Verheyden G. Trunk training following stroke. Cochrane Database of Systematic Reviews. 2023(3).	Ineligible intervention: Non telerehabilitation intervention
19	Alarcón-Aldana AC, Callejas-Cuervo M, Bo APL. Upper Limb Physical Rehabilitation Using Serious Videogames and Motion Capture Systems: A Systematic Review. Sensors (Basel). 2020;20(21).	Ineligible population: a mixture of clinical conditions such as cerebral palsy, stroke, upper limb lesions, Friedreich`s ataxia and lesions due to injury to the brain.
20	Agostini M, Moja L, Banzi R, Pistotti V, Tonin P, Venneri A, Turolla A. Telerehabilitation and recovery of motor function: a systematic	Ineligible populations: (i.e. neurological, total knee arthroplasty (TKA), cardiac) patients

	review and meta-analysis. JOURNAL OF TELEMEDICINE AND TELECARE. 2015;21(4):202-13.	
21	Perrochon A, Borel B, Istrate D, Compagnat M, Daviet J-C. Exercise-based games interventions at home in individuals with a neurological disease: A systematic review and meta-analysis. Annals of physical and rehabilitation medicine. 2019;62(5):366-78.	Ineligible population: a mixture of neurological disorders such as stroke, Parkinson's disease, and multiple sclerosis.
22	Hammer A, Nilsagård Y, Wallquist M. Balance training in stroke patients–a systematic review of randomized, controlled trials. Advances in Physiotherapy. 2008;10(4):163-72.	Ineligible intervention: Non telerehabilitation intervention
23	Trialists OS, Group CS. Therapy-based rehabilitation services for stroke patients at home. Cochrane Database of Systematic Reviews. 1996;2010(1).	Ineligible intervention: The review focuses on therapy-based rehabilitation services at home without using a telerehabilitation approach.
24	Aziz NA, Leonardi-Bee J, Phillips MF, Gladman J, Legg LA, Walker M. Therapy-based rehabilitation services for patients living at home more than one year after stroke. Cochrane Database of Systematic Reviews. 2008:N.PAG-N.PAG.	Ineligible intervention: The review focuses on therapy-based rehabilitation services at home without using a telerehabilitation approach.
25	Aminov A, Rogers JM, Middleton S, Caeyenberghs K, Wilson PH. What do randomized controlled trials say about virtual rehabilitation in stroke? A systematic literature	Ineligible intervention: Virtual reality is not based on telerehabilitation.

	review and meta-analysis of upper-limb and cognitive outcomes. J Neuroeng Rehabil. 2018;15.	
26	Dominguez-Tellez P, Moral-Munoz JA, Casado-Fernandez E, Salazar A, Lucena-Anton D. [Effects of virtual reality on balance and gait in stroke: a systematic review and meta-analysis]. Efectos de la realidad virtual sobre el equilibrio y la marcha en el ictus: revision sistematica y metaanalisis. 2019;69(6):223-34.	Ineligible intervention: Virtual reality is not based on telerehabilitation.
27	Doumas I, Everard G, Dehem S, Lejeune T. Serious games for upper limb rehabilitation after stroke: a meta-analysis. J Neuroeng Rehabil. 2021;18.	Ineligible intervention: Intervention is not based on telerehabilitation.
28	Ahn S, Hwang SJ. Virtual rehabilitation of upper extremity function and independence for stoke: a meta-analysis. JOURNAL OF EXERCISE REHABILITATION. 2019;15(3):358-69.	Ineligible intervention: Virtual reality is not based on telerehabilitation.
29	Iruthayarajah J, McIntyre A, Cotoi A, Macaluso S, Teasell R. The use of virtual reality for balance among individuals with chronic stroke: a systematic review and meta-analysis. TOPICS IN STROKE REHABILITATION. 2017;24(1):68-79.	Ineligible intervention: Virtual reality is not based on telerehabilitation.
30	McGlinchey MP, James J, McKevitt C, Douiri A, Sackley C. The effect of rehabilitation	Ineligible intervention: Non telerehabilitation intervention

	interventions on physical function and immobility-related complications in severe stroke: a systematic review. BMJ OPEN. 2020;10(2).	
31	Sevcenko K, Lindgren I. The effects of virtual reality training in stroke and Parkinson's disease rehabilitation: a systematic review and a perspective on usability. EUROPEAN REVIEW OF AGING AND PHYSICAL ACTIVITY. 2022;19(1).	Ineligible population: patients with stroke or Parkinson's disease.
32	Gandhi DBC, Sterba A, Kate MP, Khatter H, Pandian JD. COMPUTER GAME-BASED REHABILITATION FOR POST-STROKE UPPER LIMB DEFICITS- SYSTEMATIC REVIEW AND META-ANALYSIS. INTERNATIONAL JOURNAL OF PHYSIOTHERAPY. 2020;7(1):47-53.	Ineligible intervention: Non telerehabilitation intervention
33	Nikolaev V, Safonicheva O, Nikolaev A. Telerehabilitation of Post-Stroke Patients with Motor Function Disorders: A Review. ADVANCES IN GERONTOLOGY. 2022;12(3):339-46.	Not systematic review
34	Maida E, Bresciamorra V, Triassi M, Lanzillo R, Bonavita S, Lavorgna L. Cost-Analysis of Telemedicine Interventions Compared with Traditional Care in the Management of Chronic Neurological Diseases: A Systematic Review. TELEMEDICINE AND E-HEALTH. 2023.	Ineligible population: patients with chronic neurological diseases.

35	Laver K, Lange B, George S, Deutsch J, Saposnik G, Crotty M. Virtual reality for stroke rehabilitation. Cochrane Database of Systematic Reviews. 2017(11).	Ineligible intervention: Virtual reality is not based on telerehabilitation.
36	Carswell C, Rea PM. What the tech? The management of neurological dysfunction through the use of digital technology. Biomedical Visualisation: Volume 9: Springer; 2021. p. 131-45.	Ineligible population: patients with neurological dysfunctions such as stroke, Parkinson's disease, and multiple sclerosis.
37	Maier M, Rubio Ballester B, Duff A, Duarte Oller E, Verschure PFMJ. Effect of Specific Over Nonspecific VR-Based Rehabilitation on Poststroke Motor Recovery: A Systematic Meta-analysis. Neurorehabilitation and neural repair. 2019;33(2):112-29.	Ineligible intervention: Virtual reality is not based on telerehabilitation.
38	Rubin M, Wellik K, Channer D, Demaerschalk B. Systematic Review of Telestroke for Post-Stroke Care and Rehabilitation. CURRENT ATHEROSCLEROSIS REPORTS. 2013;15(8).	No outcomes of interest
39	Fernández-Vázquez D, Cano-de-la-Cuerda R, Navarro-López V. Haptic Glove Systems in Combination with Semi-Immersive Virtual Reality for Upper Extremity Motor Rehabilitation after Stroke: A Systematic Review and Meta-Analysis. Int J Environ Res Public Health. 2022;19(16).	Ineligible intervention: Intervention (rehabilitation gloves combined with semi-immersive virtual reality) is not based on telerehabilitation.
40	Chen Y, Abel KT, Janecek JT, Chen Y, Zheng K, Cramer SC. Home-based Technologies for	No outcomes of interest

	Stroke Rehabilitation: A Systematic Review. Int J Med Inform. 2019; 123:11-22.	
41	Freund M, Carey M, Dilworth S, Waller A, Mansfield E, Rose A, et al. Effectiveness of information and communications technology interventions for stroke survivors and their support people: a systematic review. DISABILITY AND REHABILITATION. 2021.	Ineligible intervention: Non telerehabilitation intervention
42	Carbajal-Galarza MM, Chinchihualpa-Paredes NO, Abanto-Perez SA, Lazo-Porras M. Effectiveness of technological interventions to improve upper limb motor function in people with stroke in low-and middle-income countries: Protocol for a systematic review and meta-analysis. medRxiv. 2020:2020.11. 10.20209197.	A systematic review and meta-analysis protocol
43	Neibling B, Jackson S, Hayward K, Barker R. Perseverance with technology-facilitated home-based upper limb practice after stroke: a systematic mixed studies review. JOURNAL OF NEUROENGINEERING AND REHABILITATION. 2021;18(1).	No outcomes of interest
44	Chien WT, Chong YY, Tse MK, Chien CW, Cheng HY. Robot-assisted therapy for upper-limb rehabilitation in subacute stroke patients: A systematic review and meta-analysis. Brain Behav. 2020;10(8):e01742.	Ineligible intervention: Non telerehabilitation intervention

45	Mehrholz J, Pohl M, Platz T, Kugler J, Elsner B. Electromechanical and robot-assisted arm training for improving activities of daily living, arm function, and arm muscle strength after stroke. Cochrane Database of Systematic Reviews. 2018(9).	Ineligible intervention: Non telerehabilitation intervention
46	Chi NF, Huang YC, Chiu HY, Chang HJ, Huang HC. Systematic Review and Meta-Analysis of Home-Based Rehabilitation on Improving Physical Function Among Home-Dwelling Patients With a Stroke. ARCHIVES OF PHYSICAL MEDICINE AND REHABILITATION. 2020;101(2):359-73.	Ineligible intervention: Non telerehabilitation intervention
47	Huang H-C, Huang Y-C, Lin M-F, Hou W-H, Shyu M-L, Chiu H-Y, Chang H-J. Effects of Home-Based Supportive Care on Improvements in Physical Function and Depressive Symptoms in Patients With Stroke: A Meta-Analysis. Archives of physical medicine and rehabilitation. 2017;98(8):1666-77.e1.	Ineligible intervention: Non telerehabilitation intervention
48	Obembe A, Odole A, Akinnawo A, editors. THE ROLE OF TELEHEALTH PHYSICAL THERAPY IN STROKE: A SYSTEMATIC REVIEW. INTERNATIONAL JOURNAL OF STROKE; 2021: SAGE PUBLICATIONS LTD 1 OLIVERS YARD, 55 CITY ROAD, LONDON EC1Y 1SP, ENGLAND.	(Conference abstract)
49	J. M, K. K, A. J, N. H. Review of telerehabilitation for strokerelated deficits.	(Conference abstract)

	Neurorehabilitation and Neural Repair. 2019;33(12):1084.	
50	Li Y, Huang J, Li X, Qiao J, Huang X, Yang L, Yu H. Effect of Time-Dose-Matched Virtual Reality Therapy on Upper Limb Dysfunction in Patients Poststroke: A Meta-Analysis of Randomized Controlled Trials. Arch Phys Med Rehabil. 2022;103(6):1131-43 e7.	Ineligible intervention: non-telerehabilitation intervention
51	H.X. H. Virtual reality in chronic stroke rehabilitation: A systematic review of its effectiveness for hemiparetic upper limb retraining. Annals of the Academy of Medicine Singapore. 2010;39(11):S62.	(Conference abstract)
52	Laver KE, Schoene D, Crotty M, George S, Lannin NA, Sherrington C. Telerehabilitation services for stroke. The Cochrane database of systematic reviews. 2013(12):CD010255.	Old version of an included review
53	Vloothuis JD, Mulder M, Veerbeek JM, Konijnenbelt M, Visser-Meily JM, Ket JC, et al. Caregiver-mediated exercises for improving outcomes after stroke. Cochrane Database Syst Rev. 2016;12(12):CD011058	Ineligible intervention: Non telerehabilitation intervention
54	Lee KE, Choi M, Jeoung B. Effectiveness of Rehabilitation Exercise in Improving Physical Function of Stroke Patients: A Systematic Review. Int J Environ Res Public Health. 2022;19(19).	Ineligible intervention: Non telerehabilitation intervention

55	Prosperini L, Tomassini V, Castelli L, Tacchino A, Brichetto G, Cattaneo D, Solaro C. Exergames for balance dysfunction in neurological disability: a meta-analysis with meta-regression. JOURNAL OF NEUROLOGY. 2021;268(9):3223-37.	Ineligible population: patients affected by acquired neurological disabilities.
56	Rosly M, Rosly H, Davis G, Husain R, Hasnan N. Exergaming for individuals with neurological disability: a systematic review. DISABILITY AND REHABILITATION. 2017;39(8):727-35.	Ineligible population: patients with neurological disabilities such as spina bifida, CP , SCI, and stroke.
57	Triantafyllidis A, Segkouli S, Zygouris S, Michailidou C, Avgerinakis K, Fappa E, et al. Mobile App Interventions for Parkinson's Disease, Multiple Sclerosis and Stroke: A Systematic Literature Review. Sensors (Basel). 2023;23(7).	Ineligible population: patients with Parkinson's disease, stroke, and multiple sclerosis.
58	Knepley KD, Mao JZ, Wieczorek P, Okoye FO, Jain AP, Harel NY. Impact of Telerehabilitation for Stroke-Related Deficits. Telemed J E Health. 2021;27(3):239-46.	(Conference abstract)
59	Xiaoyan Z, Pu W, Lijiao Y. Home-based telerehabilitation for stroke survivors: a systematic review. Chinese J Evidence-Based Med. 2019;19(10):1226-32.	Not available in English language
60	Schiza E, Matsangidou M, Neokleous K, Pattichis CS. Virtual Reality Applications for	Ineligible population: people living with dementia (PwD), stroke, spinal cord injury, Parkinson's, multiple sclerosis, and phantom upper limb pain.

	Neurological Disease: A Review. Frontiers in robotics and AI. 2019;6(101749350):100.	
61	Goldberg C, Winterbottom L, Geller D, Nilsen DM, Mahoney D, Gillen G. Technology-Related Interventions to Improve Performance in Activities of Daily Living for Adults With Stroke (2012-2019). Am J Occup Ther. 2023;77(Suppl 1).	Not systematic Review
62	Kairy D, Lehoux P, Vincent C, Visintin M. A systematic review of clinical outcomes, clinical process, healthcare utilization and costs associated with telerehabilitation. Disabil Rehabil. 2009;31(6):427-47.	Ineligible population: individuals with physical impairments
63	Lang S, McLelland C, MacDonald D, Hamilton D. Do digital interventions increase adherence to home exercise rehabilitation? A systematic review of randomised controlled trials. ARCHIVES OF PHYSIOTHERAPY. 2022;12(1).	Ineligible population: patients with different conditions such as knee osteoarthritis, frozen shoulder, ankle sprain, flexor digitorium profundus repair, generic musculoskeletal (MSK) conditions, and stroke.
64	Kendall B, Gothe N. Effect of Aerobic Exercise Interventions on Mobility among Stroke Patients A Systematic Review. AMERICAN JOURNAL OF PHYSICAL MEDICINE & REHABILITATION. 2016;95(3):214-24.	Ineligible intervention: Non telerehabilitation intervention
65	van de Port I, Wood-Dauphinee S, Lindeman E, Kwakkel G. Effects of exercise training programs on walking competency after stroke	Ineligible intervention: Non telerehabilitation intervention

	- A systematic review. AMERICAN JOURNAL OF PHYSICAL MEDICINE & REHABILITATION. 2007;86(11):935-51.	
66	Peurala SH, Karttunen AH, Sjogren T, Paltamaa J, Heinonen A. Evidence for the effectiveness of walking training on walking and self-care after stroke: a systematic review and meta-analysis of randomized controlled trials. Journal of rehabilitation medicine. 2014;46(5):387-99.	Ineligible intervention: Non telerehabilitation intervention
67	Deepa S, Priya K. Neurorehabilitation And Technology– A Systematic Review And Meta-Analysis. International Journal of Research in Pharmaceutical Sciences. 2020;11((SPL 4)):1758-65.	Ineligible population: people with neurological conditions.
68	Jung M, Steeves E. Effects of virtual reality based physical therapy interventions on balance and gait performances of chronic hemiparetic stroke patients: a literature review. Internat Med. 2019;1(2):1.	Ineligible intervention: Virtual reality is not based on telerehabilitation.
69	Aguiar L, Nadeau S, Martins J, Teixeira-Salmela L, Britto R, Faria C. Efficacy of interventions aimed at improving physical activity in individuals with stroke: a systematic review. DISABILITY AND REHABILITATION. 2020;42(7):902-17.	Ineligible intervention: Non telerehabilitation intervention
70	Vibhuti, Kumar N, Kataria C. Efficacy assessment of virtual reality therapy for	Ineligible population: people with neuromotor disorders i.e., Stroke, SCI, CP, PD.

	neuromotor rehabilitation in home environment: a systematic review. Disability and rehabilitation Assistive technology. 2023;18(7):1200-20.	
71	Amlani S, Turner G, Jeanne A, editors. In-home stroke rehabilitation in the Edmonton Zone: The Alberta provincial stroke strategy (APSS). Stroke; 2011: LIPPINCOTT WILLIAMS & WILKINS 530 WALNUT ST, PHILADELPHIA, PA 19106-3621 USA.	(Conference abstract)
72	Pollock A, Farmer S, Brady M, Langhorne P, Mead G, Mehrholz J, van Wijck F. Interventions for improving upper limb function after stroke. Cochrane Database of Systematic Reviews. 2014(11).	Not systematic review: Overview of reviews
73	Furlan A, Irvin E, Munhall C, Giraldo-Prieto M, Fullerton L, Mcmaster R, et al. REHABILITATION SERVICE MODELS FOR PEOPLE WITH PHYSICAL AND/OR MENTAL DISABILITY LIVING IN LOW- AND MIDDLE-INCOME COUNTRIES: A SYSTEMATIC REVIEW. JOURNAL OF REHABILITATION MEDICINE. 2018;50(6):487-98.#	Ineligible population: people with physical and/or mental disability.
74	Block V, Pitsch E, Tahir P, Cree B, Allen D, Gelfand J. Remote Physical Activity Monitoring in Neurological Disease: A Systematic Review. PLOS ONE. 2016;11(4).	Ineligible population: a mixture of neurological disorders such as stroke, Parkinson's disease, multiple sclerosis, dementia, traumatic brain injury and ataxia.

75	Aramaki AL, Sampaio RF, Reis ACS, Cavalcanti A, Dutra F. Virtual reality in the rehabilitation of patients with stroke: an integrative review. Arq Neuropsiquiatr. 2019;77(4):268-78.	Ineligible intervention: Virtual reality is not based on telerehabilitation.
76	Sousa C, Lee K, Alon D, Sternad D, Lu A. A Systematic Review and Meta-analysis of the Effect of Active Video Games on Postural Balance. ARCHIVES OF PHYSICAL MEDICINE AND REHABILITATION. 2023;104(4):631-44.	Ineligible population: patients with or without neurological impairment.
77	Rüth M, Schmelzer M, Burtniak K, Kaspar K. Commercial exergames for rehabilitation of physical health and quality of life: a systematic review of randomized controlled trials with adults in unsupervised home environments. FRONTIERS IN PSYCHOLOGY. 2023;14.	Ineligible population: patients with multiple health conditions such as stroke, MS, prostate cancer, lower limb amputation, rheumatoid arthritis, spinal cord injury… etc.
78	Park S, Tang A, Pollock C, Sakakibara BM. Telerehabilitation for lower extremity recovery poststroke: a systematic review and meta-analysis protocol. BMJ open. 2022;12(3):e055527.	A systematic review and meta-analysis protocol
79	Chan K, Jiang Y, Choo W, Ramachandran H, Lin Y, Wang W. Effects of exergaming on functional outcomes in people with chronic stroke: A systematic review and meta-analysis. JOURNAL OF ADVANCED NURSING. 2022;78(4):929-46.	Ineligible intervention: Intervention is not home-based

80	Mubin O, Alnajjar F, Jishtu N, Alsinglawi B, Al Mahmud A. Exoskeletons With Virtual Reality, Augmented Reality, and Gamification for Stroke Patients' Rehabilitation: Systematic Review. JMIR rehabilitation and assistive technologies. 2019;6(2):e12010.	Ineligible intervention: Intervention is not home-based
81	Lu R, Lloyd-Randolfi D, AlHeresh R, Connor L. Physical Activity Interventions Post-Stroke at Home and Methods of Assessing Adherence: A Systematic Review. Archives of Physical Medicine and Rehabilitation. 2017;98(12):e180.	(Conference abstract)
82	Todhunter-Brown A, Baer G, Campbell P, Choo P, Forster A, Morris J, et al. Physical rehabilitation approaches for the recovery of function and mobility following stroke. Cochrane Database of Systematic Reviews. 2014(4).	Ineligible intervention: Non telerehabilitation intervention
83	Thomas LH, Coupe J, Mcmahon N, Connell L, French B, Harrison J, et al. Repetitive task training after stroke: A Cochrane review [Abstract no. 116]. International Journal of Stroke. 2016;11(4_supp):59.	Not systematic review (abstract only)
84	French B, Thomas LH, Coupe J, McMahon NE, Connell L, Harrison J, et al. Repetitive task training for improving functional ability after stroke. The Cochrane database of systematic reviews. 2016;11(100909747):CD006073.	Ineligible intervention: Non telerehabilitation intervention

85	O'Connor SR, Kee F, Thompson DR, Cupples ME, Donnelly M, Heron N. A review of the quality and content of mobile apps to support lifestyle modifications following a transient ischaemic attack or 'minor' stroke. Digit Health. 2021;7:20552076211065271.	No outcomes of interest.
86	Britton M. [Home rehabilitation after stroke. Review of the literature]. Hemrehabilitering efter slaganfall En litteraturoversikt. 1997;112(9):323-6.	Not available in English language
87	Westlake K, Akinlosotu R, Udo J, Shipper A, Waller S, Whitall J. Some home-based self-managed rehabilitation interventions can improve arm activity after stroke: A systematic review and narrative synthesis. FRONTIERS IN NEUROLOGY. 2023;14.	Ineligible intervention: Non telerehabilitation intervention
88	Fryer C, Luker J, McDonnell M, Hillier S. Self management programmes for quality of life in people with stroke. Cochrane Database of Systematic Reviews. 2016(8).	Ineligible intervention: Non telerehabilitation intervention
89	Muqarrobin A, Chayati N. The effect of self-management interventions on improving the capability of daily living activities (ADL) in stroke patients: <i>a systematic review</i>. BALI MEDICAL JOURNAL. 2021;10(3):1319-28.	Ineligible intervention: Non telerehabilitation intervention
90	Mehta S, Pereira S, Viana R, Mays R, Mcintyre A, Janzen S, Teasell RW. Resistance Training for Gait Speed and Total Distance Walked	Ineligible intervention: Non telerehabilitation intervention

	During the Chronic Stage of Stroke: A Meta-Analysis. Topics in Stroke Rehabilitation. 2012;19(6):471-8.	
91	Sidiakina I, Dobrushina O, Liadov K, Shapovalenko T, Romashin O. The role of evidence-based medicine in the neurorehabilitation: the innovative technologies (a review). Voprosy Kurortologii, Fizioterapii, i Lechebnoi Fizicheskoi Kultury. 2015;92(3):53-6.	Not available in English language
92	Winkel A, Ekdahl C, Gard G. Early discharge to therapy-based rehabilitation at home in patients with stroke: a systematic review. Physical Therapy Reviews. 2008;13(3):167-87.	Ineligible intervention: Non telerehabilitation intervention
93	Brady BK, McGahan L, Skidmore B. Systematic review of economic evidence on stroke rehabilitation services. Int J Technol Assess Health Care. 2005;21(1):15-21.	Ineligible intervention: rehabilitation intervention after stroke including stroke unit (SU), care, early supported discharge (ESD) services, and community-based rehabilitation, not including telerehabilitation.
94	Nussbaum R, Kelly C, Quinby E, Mac A, Parmanto B, Dicianno BE. Systematic Review of Mobile Health Applications in Rehabilitation. Archives of Physical Medicine & Rehabilitation. 2019;100(1):115-27.	No outcomes of interest.<br>The purpose of this systematic review is to better define how mHealth apps have been used in environments relevant to physical medicine and rehabilitation
95	Standen PJ, Brown DJ, Battersby S, Walker M, Connell L, Richardson A, et al. A study to evaluate a low cost virtual reality system for	Not systematic review

	home based rehabilitation of the upper limb following stroke. 2011.	
96	H. M, S. S, S. D, M. A, H. W. Successful features and types of mobile applications for stroke rehabilitation: A systematic review. Neurorehabilitation and Neural Repair. 2023;37(5):4S-5S.	(Conference abstract)
97	Hamel R. Review of ViaTherapy Mobile Application for Upper Extremity Stroke Rehabilitation. Philadelphia, Pennsylvania: Taylor & Francis Ltd; 2018 2018-6-15. 298-9 p.	Not systematic review
98	Stretton CM, Mudge S, McPherson KM, Kayes NM. Can we improve real world walking after stroke? A systematic review and meta-analysis. Archives of Physical Medicine and Rehabilitation. 2014;95(10):e15.	(Conference abstract)
99	George S, Gustafsson L. Strong evidence for activity of daily living interventions improving functional performance in home and community settings in stroke rehabilitation. Australian Occupational Therapy Journal. 2017;64(1):87-8.	(Conference abstract)
100	Putrino D. Telerehabilitation and emerging virtual reality approaches to stroke rehabilitation. Current Opinion in Neurology. 2014;27(6):631-6.	Not systematic review

101	Khalid S, Alnajjar F, Gochoo M, Renawi A, Shimoda S. Robotic assistive and rehabilitation devices leading to motor recovery in upper limb: a systematic review. Disability & Rehabilitation: Assistive Technology. 2023;18(5):658-72.	Ineligible population: a mixture of health conditions including Spinal Cord Injury, Stroke, Cerebral Palsy, Multiple Sclerosis, and old patients.
102	Gougeh R, Falk T. Head-Mounted Display-Based Virtual Reality and Physiological Computing for Stroke Rehabilitation: A Systematic Review. FRONTIERS IN VIRTUAL REALITY. 2022;3.	Ineligible intervention: Non telerehabilitation intervention
103	R. L-L, J. G-M, F.A. V-R, P. R-P. Use of new technologies in the treatment of post-stroke hemiplegia. European Geriatric Medicine. 2017;8:S174.	(Conference abstract)
104	Lu R, Lloyd-Randolfi D, Jones H, Connor L, AlHeresh R. Assessing adherence to physical activity programs post-stroke at home: A systematic review of randomized controlled trials. TOPICS IN STROKE REHABILITATION. 2021;28(3):207-18.	No outcomes of interest.
105	Nascimento LR, Rocha RJ, Boening A, Ferreira GP, Perovano MC. Home-based exercises are as effective as equivalent doses of centre-based exercises for improving walking speed and balance after stroke: a systematic review. Journal of physiotherapy. 2022;68(3):174-81.	Ineligible intervention: Non telerehabilitation intervention

106	Stephenson A, Howes S, Murphy PJ, Deutsch JE, Stokes M, Pedlow K, McDonough SM. Factors influencing the delivery of telerehabilitation for stroke: A systematic review. PLoS One. 2022;17(5):e0265828.	No outcomes of interest.
