# Supplementary material for: Telerehabilitation and Its Impact Following Stroke: An Umbrella Review of Systematic Reviews
Source: J Clin Med. 2024 Dec 26;14(1):50. doi: 10.3390/jcm14010050 (PMC11721391; doi:10.3390/jcm14010050)
Supplement: Supplementary file 1 [file jcm-14-00050-s001.zip › Table S5 Characteristics of the included reviews.pdf]

Table S5/A: Characteristics of the included systematic reviews:

Review/objectives	Search details			Included primary studies characteristics			Participants characteristics				
	**Databases**	**Language restrictions**	**Date restrictions**	**The number and design of relevant primary studies**	**Date Range of included primary studies**	**Countries of origin of primary studies**	**Total number of participants**	**Age**	**Gender**	**Type of stroke**	**Stroke phase**
**Appleby et al. (2019)[1]**<br><br>To update the current evidence base on the effectiveness of telerehabilitation for stroke.	<u>Databases (n= 7):</u> Medline Ovid, Embase, Emcare, Scopus, The Cochrane Library, PEDro, and OT Seeker. Also, reference lists and grey literature were searched.	English language	from inception to April 2018.	(13) RCTs	published between 2007 and 2017	Belgium(N=1), Italy (N=2), Spain(N=1), Taiwan (N=1), USA(N=5), China(N=1), Slovenia (N=2).	324 participants	Range from 28-85 years.	Gender bias towards men (more male participants than female).	Ischemic stroke or hemorrhagic stroke (N= 2 studies); Ischemic stroke in MCA (N=2 studies); cortical or subcortical stroke (2 studies), N/R (N=7)	Not reported n/r
**Alayat et al. (2022)[2]**<br><br>To investigate the effectiveness of telerehabilitation on improving balance and functional mobility in stroke survivors.	<u>Databases and registers (n= 9):</u> PubMed, MEDLINE, Cochrane Central Register of Controlled Trials, PEDro, Wiley Online Library, EBSCO, ScienceDirect, REHABDATA, RCT registration website. Grey literature, and reference lists.	English language	from inception to May 2022.	(7) RCTs and (7) pilot trials.	2012-2022	n/r	594 participants	n/r	Included both genders	N/R	n/r

**Bok et al. (2023)[3]**<br><br>To identify the effect size of different technologies by systematically reviewing and analysing studies that applied technology in home-based stroke rehabilitation programs.	<u>Databases (n=5):</u><br>PubMed, Embase, CINAHL, Cochrane Library and PsycINFO. Also, Google Scholar.	English language	From inception to 28 February 2021.	(10) RCTs	2014-2020	n/r	761 participants	The mean ages were 60.4 and 59.3 years	n/r	n/r	Subacute and chronic stroke. (ranged between 3 and 12 months post-stroke.)
**Chen et al. (2015)[4]**<br><br>To determine whether telerehabilitation leads to an improvement in abilities of activities of daily living for stroke patients.	<u>Databases (n=7):</u><br>Cochrane Library, AMED, Medline, Embase, CINAHL, PyschINFO and Web of Sciences.<br>Hand searching for confernce literature between 2000 and 2015.	No language restrictions.	From inception to March 2015.	(11) RCTs	2004-2015	Netherlands (N=2), Italy (N=2), Spain (N=1), USA (N=3), Canada (N=1), China (N=1), Malysia(N=1).	1025 participants	Ranging from 53 to 74 years	N/R	n/r	n/r
**Coupar et al. (2012)[5]**<br><br>To determine the effects of home-based therapy programmes for upper limb recovery in patients with upper limb impairment following stroke.	<u>Databases (n=10):</u><br>AMED; Cochrane Library; Embase; MEDLINE; CINAHL; CENTRAL; PEDro; Otseeker; Chartered Society of Physiotherapy Research Database and REHABDATA.	No language restrictions.	n/r	(2) RCTs	2008-2009	n/r	46 participants	Range from 53 years to 66 years	n/r	Ischemic stroke (N=2).	n/r
**Deshmukh and Madhavan (2023)[6]**<br><br>To analyze primary studies investigating	<u>Databases (n= 3):</u><br>PubMed, Embase and CINAHL.	English language	2000- 2022	(4) RCTs and (2) pilot studies.	2012- 2021	n/r	215 participants	50 -80 years of age.	The higher number of males.	n/r	Acute phase (n=2); chronic phase (n=4).

the effects of telerehabilitation on walking outcomes for the treatment of adult stroke survivors.											
**Everard et al. (2021)[7]**<br><br>To determine the effect of self-rehabilitation on motor outcomes, in comparison to conventional rehabilitation, among patients with stroke.<br><br>To assess the influence of trial location, technology, time since stroke, dose, and intervention design on the effect of self-rehabilitation.	<u>Databases (n=4):</u><br>Embase; Cochrane Library; PubMed; and SCOPUS.<br>Hand searching.	English, French, or Spanish language.	From 2010 to July 2021.	(22) RCTs	2008-2021	UK(N=2); Singapore(N=1); Spain (N=2); Chile (N=1); China(N=1); South Korea(N=1); USA (N=5); Germany(N=2); Australia (N=1); Netherlands (N=2); Taiwan (N=1); Italy (N=1); Israel(N=1); New Zealand (N=1)	1175 participants	Range from 55.5 years to 75.1 years	n/r	n/r	Subacute and Chronic stroke
**Hao et al. (2023)[8]**<br><br>To gather and summarize evidence of virtual reality telerehabilitation for patients after stroke and compare it with conventional in-person rehabilitation.	<u>Databases (n=6):</u><br>PubMed, Embase, PsycINFO, IEEE Explore, Cumulative Index of Nursing and Allied Health.	English language	Published after 2000	(9) RCTs	2006-2021	n/r	260 participants	n/r	n/r	Ischemic stroke (N=3)<br>N/R(N=6)	Chronic stage, which was 6 months after the onset (N=8).<br><br>Patients from 1 to 10 months post stroke(N=1)

**Hwang et al. (2021)[9]**<br><br>To identify the focus of TH-SM interventions and SM support components in stroke survivors.<br><br>To identify the type of TH delivery.<br><br>To identify the effects of TH-SM support in stroke survivors.	Databases (n=5):<br>Embase; Medline, CINAHL, PsycINFO, and Web of Science.	n/r	between 2005 and 2020.	(2) RCTs and (1) pilot study.	2009-2015	n/r	122 participants	n/r	n/r	n/r	Acute and chronic(N=2); N/R(N=1)
**Johansson and Wild (2011)[10]**<br><br>1-To explore the feasibility, effectiveness, cost-effectiveness, and quality of telerehabilitation interventions in post-stroke care.<br>2-To explore the effect of post-stroke telerehabilitation initiatives on health outcomes, health-care processes, the use of health resources, and user/patient satisfaction and acceptance.	Databases (n= 4):<br>Medline Ovid, Embase, DARE-NHSEED (INAHTA), The Cochrane Library.	n/r	from 1995-2009.	(4) RCTs	All published after 2000	USA (N=1); Netherland (N=2); Italy (N=1).	662 participants	n/r	n/r	Ischemic stroke (N=2); N/R (N=2).	n/r

**Lazem et al. (2023)[11]**<br><br>To present the extent of evidence concerning the effectiveness of extended reality telerehabilitation and patients' experiences of using different types of virtual reality exercises at home.	Databases (n=6):<br>PubMed, Embase, Medline, Web of Science, CINAHL, and PEDro.	English	from inception until 15 January 2023	(9) RCTs	2009- 2022	The UK (N=1); the USA (N=2); Spain (N=2); Hong Kong (N=1); Korea (N=1);Italy (N=1) and Canada (N=1).	The total number of participants across RCTs: 680	The mean age of participants ranged from 55.4 to 68 years.	n/r	Most strokes are hemorrhagic.	Subacute or Chronic (N= 2), Chronic only (n=7)
**Laver et al. 2020[12]**<br><br>1- to determine whether the use of telerehabilitation leads to improved ability to perform activities of daily living amongst stroke survivors when compared with (1) in-person rehabilitation or (2) no rehabilitation or usual care.<br>2- to determine whether the use of telerehabilitation leads to greater independence in self-care and domestic life and improved mobility, balance, health-related quality of life, depression, upper limb function, cognitive	Databases (n= 11):<br>Cochrane Central Register of Controlled Trials (the Cochrane Library, Issue 6, 2019),<br>MEDLINE Ovid,<br>Embase<br>AMED Ovid<br>CINAHL EBSCO,<br>PsycINFO Ovid<br>PsycBITE,<br>OTseeker,<br>Physiotherapy Evidence Database,<br>REHABDATA,<br>Health Technology Assessment Database (HTA)<br>Trials registers (n=3):<br>Cochrane Stroke Group Trial Register,<br>US National Institutes of Health Ongoing Trials Register ClinicalTrials.gov, and World Health	No language restrictions	from inception to June 2019	22(RCTs)	All studies were published between 2004 and 2019).	Included studies were conducted in the USA (n = 8), Canada (n = 3), the Netherlands (n = 2), Italy (n = 2), Germany (n = 2), China (n = 2), Taiwan (n = 1), Spain (n = 1), and Slovenia (n = 1).	1937 participants	Ranged from 50 to 70.	Similar numbers of men and women(n=20) except (n=2 studies) only recruited men.	n/r	Acute stage (n=8)<br>Subacute and chronic (n= 14)

function or functional communication when compared with in-person rehabilitation and no rehabilitation.<br>3- to report on the presence of adverse events, cost-effectiveness, feasibility and levels of user satisfaction associated with telerehabilitation interventions.	Organization International Clinical Trials Registry Platform.<br>Gray literature.											
**Lombardo & Islam (2023)[13]**<br><br>To explore stroke survivors' acceptance and satisfaction of telerehabilitation delivery of physiotherapy services.	Databases (n=8):<br>Embase (Ovid); CINAHL; Informit; ProQuest; PubMed; ScienceDirect; SCOPUS and SpringerLink.<br>Manual searching of reference lists.	English language	From 2010 to July 2021.	(1) RCT	2015	n/r		52 participants	n/r	n/r	Ischemic or haemorrhagic.	n/r
**Nascimento et al. (2022)[14]**<br><br>To examine the effects of home-based exercises in comparison with centre-based exercises for improving the paretic UL after stroke.	Databases (n=6):<br>AMED; Cochrane Library; Embase; MEDLINE; PsycINFO and PEDro.	No language restriction	No date restriction	(3) RCTs	2008-2019	n/r		170 participants	range from 61 to 65 years	n/r	n/r	Acute and Chronic (N=2), Chronic (N1)

**Ostrowaska et al. (2021)[15]**<br><br>To analyse the benefits and limitations of teletherapy on the functional condition of post-stroke patients.	Databases (n= 3): PUBMED, Directory of Open Access Journals, and Science Direct Database.	English language	2019- 2021.	(3) RCTs	2019-2021.	In the USA (n = 1), n/r (n=2)	98 participants	The average age is between 57 and 62 years old.	The number of women and men varied across studies (Range: 4- 34 women versus 6- 90 men)	Ischemic or haemorrhagic stroke (n=3 studies)	Subacute or Chronic Range: 4- 36 weeks (N= 2), N/R (n=1)
**Qin et al. (2022)[16]**<br><br>To investigate the effectiveness of home-based interventions in improving the ability to do basic activities of daily living in patients who had a stroke.	Databases (n=3): MEDLINE; EMBASE and CINAHL. The reference list was checked.	English language	From inception to 31 December 2021.	(5) RCTs	2012-2021	n/r	368 participants	The the mean age for each study, mean (range): 63.8 (40.8-89.6), mean (SD): 66.52 (12.08), mean (SD): 64.19 (9.42), mean (SD): 63.7 (12) .	n/r	n/r	n/r
**Rintala et al. (2019)[17]**<br><br>To study the effectiveness of technology-based distance physical rehabilitation interventions on physical functioning in stroke**.**	Databases (n=6): Embase, CINAHL, Ovid Medline, PEDro, CCRCT, and Web of Science.	English, Finnish, Swedish, or German.	From January 2000 to May 2018.	(13) RCTs	2003-2017	Australia (N=2); Spain (N=1); China (N=2); United States(N=1); Taiwan (N=1); United Kingdom (N=2); Netherlands (N=2); Italy (N=1); Malysia (N=1).	605 participants	The mean (SD) age of the study participants was 65.2 (4.2) years.	%65 of the participants were men.	%87 had experienced ischemic stroke (N=9). N/R (N=4)	Range ≤ 1 month to 36 months (N=10); N/R (N=3)
**Rintala et al. (2023)[18]**	Databases(n=3): PubMed, Web of Science, and Scopus.	English language	From inception to 12 July 2022.	(5) RCTs and (2) pilot studies.	2016-2022	UK(N=1); Spain (N=2); The Netherland (N=1); South	215	The median age of the participants	41%of the participants were women.	n/r	Chronic stage (N=4); Subacute

To evaluate the effectiveness of mobile health applications (mHealth apps) containing a physical training component on physical function and physical activity in stroke rehabilitation.						Korea(N=2); South America (Chile)(N=1)		was 59.3 (55.3 to 61.0) years			stage (N=2);a combination of acute, subacute, and early chronic and chronic stage (N=1).
**Sarfo et al. (2018)[19]**<br><br>To provide an updated systematic review on the efficacy of telerehabilitation interventions for recovery from motor, higher cortical dysfunction, and poststroke depression among stroke survivors.	<u>Databases (n= 2):</u> PubMed and Cochrane Library.	No language restriction	From January 1, 1980, to July 15, 2017.	(13) RCTs, (4) pilot trials, and (1) controlled trial-longitudinal cohort with the control group.	2007-2017	China (N=2), Brazil (N=1), Korea (N=1), UK (N=1), Australia(N=1), USA (N=5), Spain (N=1), Italy (N=3), Malaysia (N=1), Thailand (N=1), Netherland (N=1).	395	n/r	n/r	n/r	n/r
**Sharififar et al. (2023)[20]**<br><br>(1) to appraise the available evidence of telerehabilitation program effects on functional outcomes, adherence, and patient satisfaction compared to face-to-	<u>Databases (n= 7):</u> CINHAL, Cochrane Library, Embase, SCOPUS, Physiotherapy Evidence Database (PEDro), PubMed and Web of Science. Also, Gray literature.	English language	from 1964 to the end of April 2022.	(7) RCTs	Between 2012 and 2021	The included studies were conducted in the USA (n = 4), Italy (n= 1), China (n= 1), Malaysia (n=1)	711 participants	Range from 61 to 70 years	The ratio of male to female was inconsistent across studies (Range: 0-70 males versus 0-65 females).	Ischemic or haemorrhagic (N=4); n/r(N=3)	Chronic stroke(N=3); n/r(N=4)

face programs after stroke.<br>(2) to provide direction for future outcome measure selection and development for clinical research purposes.											
**Schroder et al. (2018)[21]**<br><br>To investigate the feasibility and effectiveness of home-based VR telerehabilitation on balance and functional mobility.	<u>Databases (n= 5):</u><br>PubMed, Web of Science, PEDro, Rehab Data, and Cochrane Library.	English, Dutch, German, or French.	n/r	(4) RCTs	2012-2015	n/r	106 participants	range from 55-74 years.	n/r	n/r	Late sub-acute (3-6 months post-stroke) (N= 1);<br>Chronic (more than 6 months post-stroke) (N=2);<br>n/r (N=1)
**Saragih et al. (2022)[22]**<br><br>To identify the effects of telehealth interventions on the ability to perform ADLs and maintain balance in stroke survivors.	<u>Databases (n=7):</u><br>PubMed, Embase, Medline, Web of Science, Ovid (UpToDate), Academic Search Complete and CINAHL.<br>Grey literature through Google Scholar.	English Language	Up to April 25, 2021	(6) RCTs	2009- 2020	China (N=1); USA (N=1); Spain (N=1); Canada(N=1); Austria (N=1); Taiwan(N=1).	436 participants	Range from 57 to 75 years.	Most participants were male (N=4).<br>n/r(N=2).	n/r	n/r
**Su et al. (2023)[23]**<br><br>1- To investigate the effects of telerehabilitation on the balance ability of stroke patients.	<u>Databases (n= 5):</u><br>Cochrane Library, PubMed, Embase, Web of Sciences, and the Joanna Briggs Institue databases.	English Language	From 1 January 2020 to 31 December 2022.	(9) RCTs	2020-2022	China (N=5), Spain (N=3), n/r (N=1)	328 participants	n/r	n/r	n/r	all patients had Subacute or Chronic stroke.

2- To compare the efficacy of conventional rehabilitation with telerehabilitation,<br><br>3- To explore the characteristics of telerehabilitation and conventional rehabilitation.<br><br>4- To provide recommendations for rehabilitation programs in the context of the global pandemic.											
**Szeto et al. (2023)[24]**<br><br>To review the effect of mobile apps for stroke rehabilitation on stroke impairments and functional outcomes.	<u>Databases (n=7):</u> MEDLINE; EMBASE; Cochrane Library; CINAHL; SCOPUS; COMPENDEX and IEEE Xplore.	English language	n/r	(8) RCTs and (2) pilot study	2013-2020	n/r	703 participants from RCTs only.<br>N/R in pilot study.	Range from 39 to 73 years	n/r	n/r	n/r
**Tchero et al. (2018)[25]**<br><br>To investigate the efficacy of telerehabilitation in post-stroke patients.	<u>Databases (n= 3):</u> MEDLINE, Cochrane Library, Web of Science.	No language restrictions	No date restrictions	(15) RCTs	2008-2019	n/r	1339	n/r	n/r	n/r	n/r
**Toh et al. (2022)[26]**	<u>Databases (n=4):</u> MEDLINE; CINAHL; Cochrane Library and Web of Science.	English language.	From January 2000 to September 2020.	(8) RCTs	2009-2020	n/r	576	The mean age of participants ranged from	n/r	n/r	Chronic stroke

To determine the effects of home-based upper limb rehabilitation for hemiparetic upper limb recovery in stroke survivors.	Reference lists were checked.							52.3 to 69.4 years.			
**Tarihoran et al. (2023)[27]**<br><br>To evaluate the efficacy of a videoconferencing intervention for stroke survivors.	<u>Databases (n=7):</u> PubMed, Embase, CINAHL, Medline, Ovid, Cochrane Library, and Web of Science.	English language	From inception to May 27, 2021.	(9) RCTs	2009 -2021	China (N=4); USA (N=1); New Zealand (N=1); Canada(N=1); Korea (N=1); Spain(N=1).	603 participants	Range from 55 to 73.5 years.	More than half the participants were men (53%)(N=8); n/r(N=1).	Ischemic or hemorrhagic stroke (N=1); N/R (N=8)	post stroke > 6 months (N=1); or 24 months (N=1); n/r (N=7).
**Zhou et al. (2018)[28]**<br><br>To determine the effectiveness of mobile applications for smartphones, tablets, and computers in the rehabilitation of stroke survivors and indicate the opportunities and challenges of using mobile applications in stroke management.	<u>Databases (n=4):</u> PubMed, Embase, Science Citation Index Expanded-SCIE and CINAHL.	No language restrictions	From inception to May 28, 2017.	(1) RCTs and (1) pilot study	2016	UK(N=1); Korea(N=1)	45 participants	Range from 47 to 73 years	n/r	n/r	n/r

Table S5/B: Characteristics of included systematic reviews continued

Review	Outcomes of interest	Outcome measures	Follow-up	Method of analysis	Effect size (95% cI) Heterogeneity (I^2)	Appraisal instrument used	Appraisal rating
**Appleby et al. (2019) [1]**	Primary outcomes: Motor function. Activities of daily living (ADL).<br><br>Secondary outcomes: Independence & self-efficacy.<br><br>Patient satisfaction/quality of life.<br><br>Miscellaneous: ROM, power, spasticity.	Motor Function: (FM), (FMUE), ABILHAND scale (ABILHAND), (B&B), (JTT), finger movement tracking test (Finger tracking), (BBS), Gait assessment (GA), (10MWT), Motor subscale of the Functional Independence Measure (mFONEFIM), (POMA-B), (POMA-G), (BBA), (TUG), (ARAT), (NHPT)<br><br>ADLs: (MAL), (BI), (MBI), (CAHAI)<br><br>Satisfaction/Quality of Life: (MDSQ), (TRSQ), (SSPSC-Hospital), (SSPSC-Home), (SUS), (CSI), (VAS), Short Form 12 (SF-12)<br><br>Independence/Self-efficacy: (FES), (IMI), (LLFDI), (MRS).	n/r	a descriptive synthesis	n/r	A modified McMaster Critical Appraisal Tool	moderate to good with the scores ranging from 7-12 out of a possible 14.
**Alayat et al. (2022)[2]**	Primary outcomes: Balance<br>Secondary outcomes: Functional mobility	Balance: Berg Balance Scale (BBS), Performance-Oriented Mobility Assessment -	Range from 2 weeks to 12 months (N=6), N/R (N=8)	Both descriptive synthesis and Meta-analysis.	**Effect of telerehabilitation alone or combined with conventional rehabilitation vs. various control interventions:**	PEDro scale	10 studies were rated a good quality, 3 studies were rated fair quality and 1 study were rated poor quality.

		Balance (POMA-B), Brunel Balance Assessment (BBA), Mini Balance Evaluation Systems Test (Mini-BEST), Lean-and-Release Assessment, and Step Test.<br><br>Mobility and gait:10 Meter Walk Test (10MWT), Rivermead Mobility Index (RMI), 6-Minute Walking Test (6MWT), Functional Ambulation Category (FAC), Mobility Domains of Barthel Index (BI), Stroke Impact Scale (SIS), and Nottingham Extended ADL Scale (NEADL).<br><br>For both balance and mobility: Timed Up & Go Test (TUG) and Postural Assessment Scale for Stroke Patients (PASS).		Based on the Random-effect model	Balance: Small effect size SMD: 0.33 (0.03 to 0.63) I^2= 63%<br><br>Functional mobility: small effect size SMD: 0,27 (0.02 to 0.52) I^2= 4%		
**Bok et al. (2023)[3]**	Primary outcomes: Physical function outcomes.<br>ADL<br>Balance	Physical function outcomes: Fugl–Meyer Assessment (FMA), Nine-Hole Peg Test (9HPT), Box and Block Test, Action Research Arm Test (ARAT), Tinetti Performance-Oriented Mobility Assessment (POMA),10-meter Walk Test (10MWT), and Time-Up and Go test (TUG).	n/r	Meta-analysis<br><br>Based on the Random-effect model	By type of intervention:<br>VR intervention:<br>Hedges`s g, 0.850 (0.314 - 1.385) $I^2$= 77.5%<br><br>Robot-assisted interventions:<br>Hedges`s g, 0.129 (0.025 - 0.232) I^2= 0%<br><br>game intervention:<br>Hedges`s g, -0.162 (-0.534 - 0.210) I^2= 77.4%	Cochrane risk of bias tool (ROB)	Random sequence generation: all 10 RCTs were rated as having a low risk of bias.<br><br>Concerning allocation concealment: 8 RCTs were rated as having a low risk of bias, but 2 were unclear.<br><br>Blinding participants and personnel: 6 RCTs were rated as having a low risk of bias

		ADL outcomes: the Modified Barthel Index (MBI) and Nottingham Extended ADL Index.<br><br>Balance outcomes: the Berg Balance Scale and Brunel Balance Assess (BBA)			By applied limb:<br>interventions targeting the upper limb:<br>Hedges`s g, 0.291 (0.133 - 0.449)<br>$I^2$= 62.1%<br>interventions targeting the lower limb:<br>Hedges`s g, 0.113 (0.547 to 0.32)<br>I^2= 73.3%		Blinding of outcome assessment: 8 RCTs were rated as having a low risk of bias.<br><br>Incomplete outcome: 8 RCTs were rated as having a low risk of bias.<br><br>Selective reporting: 8 RCTs were rated as having a low risk of bias.
**Chen et al. (2015)[4]**	Primary outcomes:<br>1- disability or activities of daily living.<br><br>Secondary outcomes:<br>1- motor function.<br>2- participant satisfaction.<br>3- health-related quality of life.<br>4-cost-effectiveness	Primary outcomes:<br>Disability or activities of daily living.<br>Barthel Index (BI)<br>Berg Balance Scale (BBS)<br>Functional Independence Measure scale.<br><br>Secondary outcomes:<br>assessment of motor function: Fugl- Meyer Extremity Scale Wolf Motor Function Test<br>Timed Up and Go Test<br>Action Research Arm Test<br>Nine-Hole Peg Test.<br>Walking speed.<br>Ashworth Scale.<br><br>Participant satisfaction:<br>Satisfaction with stroke care Questionnaire.<br>Satisfaction Questionnaire.<br><br>Health-related quality of life:	n/r	Both descriptive and meta-analysis<br><br>Based on the Random-effect model	Primary outcomes: Disability or activities of daily living.<br>**Duration <= 6 weeks vs Duration > 6 weeks:**<br>BI: SMD: -0.05 (-0.24 to 0.13) I^2= 27%<br><br>**Without VR-based training vs. with VR-based training**<br>BI: SMD: -0.05 (-0.24 to 0.13) I^2= 73%<br>BBS: SMD: -0.07(-0.7 to 0.37) I^2= 0%<br>Functional Independence Measure scale: data were not pooled and analysed.<br><br>Secondary outcomes:<br>assessment of motor function:<br>FMA-UE: SMD: 0.05 (-0.09 to 1.09) I^2= 0%<br>Timed Up and Go Test: not pooled and analysed.<br>Action Research Arm Test: not pooled and analysed.<br>Nine-Hole Peg Test: not pooled and analysed.	Cochrane Risk of Bias tool.	low risk of bias(N=6), high risk of bias (N=2), unclear risk (N=3).

		Short Form Health Survey					
**Coupar et al. (2012)[5]**	Performance in ADL.<br>Functional movement of the UL.	Motor impairment of the UL: Fugl-Meyer Upper Extremity Scale.	1 month(N=1).	Meta-analysis<br><br>Both Fixed and random effect model	Upper limb motor impairment:<br>**Comparison of home therapy program vs. usual care:**<br>Following intervention: (only one study Piron 2009)<br>MD: 4.10 (-0.09 to 8.29) I^2= n/r<br>at follow-up:<br>MD: 4.30 (0.19 to 8.41) I^2= 32%<br>**Comparison of home therapy program vs. the same therapy program at the hospital:**<br>MD:0.60 (-8.94 to 10.14) I^2= n/r	A standard critical appraisal form.	Generated allocation sequence; concealed allocation: reported adequately in one study.<br><br>One study did not report how randomisation sequence was generated or detail of any allocation concealment.<br><br>Blinding of outcome assessor was reported in two studies.<br><br>Two studies did not report any drop-out.
**Deshmukh and Madhavan (2023)[6]**	Primary outcomes:<br>Walking function: (speed, walking endurance, and balance).<br><br>Secondary outcomes:<br>Activity, participation and quality of life.<br>participants adherence.	Walking outcomes:<br>The 10-meter walk test (10MWT)<br>Six-minute Walk test(6MWT)<br>Berg balance scale (BBS)<br>Brunel balance assessment (BBA)<br>Timed up & go test (TUG)<br>The Tinetti Performance Oriented Mobility Assessment gait scale (POMA-G).<br><br>Activity, participation, and quality of life:<br>Stroke Specific Quality of Life Questionnaire (SS-QOL);<br>the Modified Barthel Index;<br>the Modified Rankin Scale (MRS);	n/r	Descriptive synthesis.	n/r	PEDro Scale	50% of the included studies were rated good, and the other 50% were rated fair, based on PEDro scale.<br><br>All studies reported between-group difference in at least one key outcome measure.<br><br>Due to the nature of the intervention, blinding the participant or the therapist was not possible in all studies.

		Fugl Meyer Assessment scale (FMA);<br>Motricity index (MI);<br><br>Participant adherence: measured by the rate of participant dropout.					
**Everard et al. (2021)[7]**	Body function.<br>Activity:<br>hand and arm use(reaching and grasping).<br>Walking (gait speed and balance).<br>Mobility.	Motor function:<br>FM; Muscle contraction; Grip strength; Stroke Impact Scale (SIS) hand function<br><br>Activity:<br>ARAT; 2MWT; WMFT; BBS; FIM; NHPT; TUG.	n/r	Descriptive synthesis and Meta-analysis<br><br>Random effect model	**Effect of self-rehabilitation on motor function compared to conventional therapy:**<br><br>UL: We pooled data for 10 studies (Graph S1): SMD:0.31(- 1.34 to 1.95) I^2= 40.44%<br><br>LL: SMD: -0.15 (-0.66 to 0.36) I^2= 0%<br><br>**Effect of self-rehabilitation on activity compared to conventional therapy:**<br>SMD: 0.01 (-0.14 to 0.16) I^2= 0%	Cochrane Collaborations Risk of Bias and PEDro checklist	Trials had a median PEDro Score of 6.5 and 85% of these were superior or equal to 6.<br><br>The Cochrane Collaboration Risk of Bias assessment revealed 67% reported the blinding of assessors.<br><br>Half of the trials using concealed allocation during the randomization process.
**Hao et al. (2023)[8]**	Primary outcomes:<br>Upper-extremity function:<br>Gross manual dexterity<br>Balance ability<br>Gait<br>Activities of Daily Living<br><br>Secondary outcomes:<br>Satisfaction<br>Cost-benefit analysis	Functional outcomes:<br>Upper-extremity function:<br>Fugl-Meyer Assessment<br>Gross manual dexterity:<br>Box and Block Test<br>Balance ability:<br>Berg Balance Scale<br>Timed Up and Go Test<br>Gait:<br>Two-Minute Walk Test<br>10-Meter Walk Test<br>Activities of Daily Living:<br>Barthel Index<br>European Quality of Life-5 Dimention<br>Satisfaction:<br>Satisfaction Questionnaire	Ranging from 1 to 3 months after completing intervention (N= 4).<br><br>n/r (N=5)	A descriptive synthesis and meta-analysis.<br><br>A fixed-effect model if there was little heterogeneity, or a random effect model was used instead.	Functional outcomes:<br>Upper-extremity function:<br>Fugl-Meyer Assessment: SMD:1.05 (0.04 to 2.06) I^2= 84%<br>Gross manual dexterity:<br>Box and Block Test: SMD:0.37 (0.06 to 0.79) I^2= 0%<br>Balance ability:<br>Berg Balance Scale: SMD: 0.05 (0.30 to 0.40) I^2= 0%	PEDro Scale	Seven studies were scored six to eight considered as good quality. Two studies were scored four to five considered as fair quality. Only 6 of 9 studies blinded assessors.<br><br>No study was able to blind subjects or therapists due to the nature of rehabilitation intervention.<br><br>only 4 of 9 studies reported concealed allocation.

**Hwang et al. (2021)[9]**	Physical function.<br>Satisfaction.<br>Mobility.	Physical function: Telephone Version of the Functional Independence Measure (FONEFIM)<br>Satisfaction: Stroke-Specific Patient Satisfaction with Care (SSPSC).<br>Mobility: BBS	n/r	A descriptive synthesis	n/r		PEDro scale for RCTs.<br>Risk of Bias Assessment Tool for Non-randomised Studies (RoBANS)	2 RCTs showed high quality.<br>Low scores on the scale were used to blind the subjects and therapists.<br>1 non-RCT showed a high risk of bias as there was no group to control.<br>None of the Non-RCTs studies mentioned evaluator blinding.
**Johansson and Wild (2011)[10]**	Primary outcomes: Motor function.<br><br>Secondary outcomes: Satisfaction and acceptance.<br>Cost.	Motor function:<br>Action Research Arm Test (ARAT)<br>Nine-Hole Peg Test (NHPT)<br>Fugl-Meyer Upper Extremity Scale (FMUE)<br>ABILHAND scale (ABILHAND)<br>Ashworth scale.	n/r	Descriptive analysis	n/r		n/r	Randomization procedure: not clearly described.<br><br>Lacked adequate blinding procedure<br><br>No high dropout rates were recorded.
**Lazem et al. (2023)[11]**	Primary outcomes: Upper limb function; lower limb function, balance, ADL, mobility, trunk control, gait, and falls.	Motor function:<br>Upper limb function (FMA-UE)<br>Action Research Arm Test (ARAT)<br>Lower limb function (FMA-LE)<br>Gait and balance:<br>Balance Berg Scale (BBS).<br>Timed Up and go test (TUG)<br>the performance-oriented mobility assessment balance subscale (POMa-B).<br>the performance-oriented mobility assessment gait subscale (POMa-G).	n/r	A descriptive synthesis.	n/r		Cochrane Collaboration tool.	Only two out of nine RCTs showed a low risk of bias in their overall scores; While seven showed a high risk of bias.

		Functional ambulation category (FAC)					
**Laver et al. (2020)[12]**	Primary outcomes: independence in activities of daily living.<br><br>Secondary outcomes:<br>Self-care & domestic life.<br>Mobility.<br>Balance.<br>Participant satisfaction with the intervention.<br>Self-reported health-related quality of life.<br>Upper limb function.<br>Cost-effectiveness.	Upper limb:<br>Fugl- Meyer Upper Extremity Scale, Action Research Arm Test or the Nine-Hole Peg Test<br><br>ADL:<br>Barthel Index<br>Frenchay Activities Index<br><br>Mobility:<br>Timed Up and Go test<br>Gait speed<br><br>QoL:<br>Short Form	follow up at one month (n=2), three months (n=3), six months (n=4), or 12 months(n=3), after completion intervention.	Both descriptive and meta-analysis<br><br>Meta-analysis based on random effect model with 95% CI.	**Telerehab vs in-person: Post-intervention**<br><br>1. ADL:MD: 0.59 (-55, 6.68) I^2= 0%<br><br>2. Balance: MD: 0.48 (-1.36, 2.32) I^2= 0%<br><br>3. ULF: MD: 1.23 (-2.17, 4.64) I^2= 42.41%<br><br>**Telerehab vs usual care: Post-intervention**<br><br>1. ADL: SMD: -0.00 (-0.15,0.15) I^2= 0%<br><br>2. Mobility: MD: 0.01 (-0.12,0.14) I^2= NA<br><br>3. QoL: SMD: 0.03 (-0.14, 0.2) I^2= 5.42%<br><br>4. ULF: SMD: 0.33 (-0.21,0.87) I^2= 0%	Cochrane Risk of bias tool.	Allocation concealment was adequate.<br><br>Blinding: the assessor was blinded to allocation.<br><br>Incomplete data outcome.<br><br>Selective reporting: most were free from bias.<br><br>Other potential sources of bias are small sample sizes, differences between groups at baseline, or both.
**Lombardo & Islam (2023)[13]**	Participants satisfaction	Satisfaction questionnaires and scales:<br>Strok-Specific Patient Satisfaction with Care (SSPSC) scale + interviews with participants.	n/r	Descriptive synthesis	n/r	The Mixed Methods Appraisal Tool (MMAT)	RCT study did not fulfil the criteria regarding blinding.
**Nascimento et al. (2022)[14]**	Motor recovery of hemiparetic UL.<br>Paretic UL performance.	Motor recovery of paretic UL: Fugl-Meyer Performance; Box and Block test; ABILHAND<br>Paretic UL performance: Box and Block test; ABILHAND.	n/r	Descriptive synthesis and Meta-analysis.<br><br>Random effect model	**Effects of home-based compared to center-based:**<br>Motor recovery:<br>We pooled data for 3 studies (Graph S2)<br>MD:1.29 (-2.37 to 4.96) I^2= 48.27%<br><br>Activity performance:<br>only one study (Cramer)<br>SMD: -0.12 (-0.47 to 0.22) I^2= n/r	PEDro Scale	All trials had randomly allocated participants; reported between groups differences; had blinded assessors; and had similar groups at the baseline.<br><br>2 trials had less than 15% drop-outs.

							2 trials did not report concealed allocation.<br><br>1 trial did not blinded participants or therapists.
**Ostrowaska et al. (2021)[15]**	Primary outcomes:<br>1- motor function of UL<br>2- Balance<br>3- Cardiorespiratory fitness.<br>4-Cognitive function.<br><br>Secondary outcomes:<br>1- level of acceptance<br>2-Feasibility of teletherapy for its users at home.	Functional status:<br>Fugi-Meyer Upper Assessment Upper Extremity (FMA-UE)<br>Fugi-Meyer Upper Assessment Lower Extremity (FMA-LE)<br>Berg Balance Scale (BBS)<br>Timed Up & Go Test (TUG)<br>6-min Walk Test (6 MWT)<br>Balance:<br>Berg Balance Scale (BBS)<br>System Usability Scale (SUS)	n/r	Descriptive synthesis	Effect of telerehabilitation on Functional condition of occupied UL:<br>FMA-UE: -2.14 to 2.26<br><br>Impact of telerehabilitation based on Collaborative Mode on the functional status:<br>FMA-UE: n/r<br>FMA-LE: n/r<br>BBS: n/r<br>TUG: n/r<br>6 MWT: n/r<br><br>Effect of telerehabilitation on balance improvement:<br>BBS: n/r<br>SUS: n/r	n/r	n/r
**Qin et al. (2022)[16]**	Basic ADL	Modified Barthel Index (MBI).<br>Modified Rankin Scale (mRS).<br>The motor subscale of the Telephone Version of the Functional Independence Measure (FONEFIM)	At 3 months follow-up and at 6 months follow-up (N=1);<br>At 3-month follow-up (N=1);<br>At 6 months follow-up (N=1);<br>n/r (N=2)	At 3 months follow-up and at 6 months follow-up (N=1);<br>At 3-month follow-up (N=1);<br>At 6 months follow-up (N=1);<br>n/r (N=2)	**Effectiveness of home-based intervention with institution-based intervention at treatment endpoint:**<br>for only one study/Chen 2017: SMD: 0.12 (-0.14 to 0.66) I^2= n/r<br>**Effectiveness of home-based intervention addition to usual care with usual care at the treatment endpoint:**<br>for only one study/ Chumbler 2012: SMD:0.29 (-0.31 to 0.88) I^2= n/r	PEDro Scale	Good methodological quality (PEDro score=6-8) (N=4); poor quality (PEDro=1-3)(N=1).
**Rintala et al. (2019)[17]**	Primary outcomes:<br>ADL<br>UE and LE functioning<br>Balance.<br>Walking.<br>Physical activity.<br>Participation.	ADL:<br>Barthel Index (BI)<br>Modified BI; Modified Rankin Scale (MRS)<br>telephone version of the Functional	n/r	Descriptive synthesis and meta-analysis.<br><br>Metanalysis was performed using	ADL: SMD:0.06 (-0.22 to 0.35) I^2= 38%	Furlan method guideline for SRs.	Overall methodological quality of studies was low.<br><br>The methodological quality of studies was high in four studies, Moderate in two studies and low in seven studies.

		Independence Measure (FONEFIM)<br>ADL domain of Stroke Impact Scale (SIS).<br>The Nottingham Extended ADL scale (NEADL).<br>UE functioning:<br>Late-Life Function and Disability Instrument (LLFDI)<br>Fugl-Meyer Assessment (FMA).<br>Wolf Motor Function Test.<br><br>LE functioning:<br>LE domains of (LLFDI) and (FMA).<br><br>Balance:<br>Berg Balance Scale (BBS)<br><br>Walking:<br>10-meter walk test<br>Physical activity:<br>physical activity subscale in SIS<br>Health Promoting Lifestyle Profile11.		a Random-effect model				All studies used adequate randomization methods.<br>Only 38% of studies reported allocation procedure.<br>Only 3 studies used intention-to-treat analysis.
**Rintala et al. (2023)[18]**	Primary outcomes:<br>Physical function:<br>UE function.<br>LE function.<br>Balance.<br>Walking speed.<br>ADL.<br><br>Secondary outcomes:<br>QoL	physical function:<br>UE function: manual muscle testing (MMT) of the upper extremity or wrist and fingers, Fugl-Meyer Assessment of the upper extremity (FMA-UE), Brunnstrom stage(B-stage) for the arm and hand, Manual	n/r	Descriptive synthesis.	n/r		PEDro scale and the modified Downs and Black checklist.	Overall methodological quality was fair.<br><br>For RCTs, a general defect was blinding procedure, random allocation; concealed allocation, baseline comparability; adequate follow-up, and reporting of point of measures and measures of variability.

		Function Test (MFT), and Purdue Pegboard Test (PPT)<br>LE function: Fugl-Meyer Assessment of the lower extremity(FMA-LE) and the Motricity Index of the lower extremity leg (MI-LE).<br>Balance: Mini-BESTest (MBT) and/or Berg Balance Scale (BBS).<br>Walking speed: 10-Meter Walking Test (10MWT);<br>Walking endurance: 6-Minute Walking Test (6MWT).<br>ADL: Barthel Index (BI), modified Barthel Index (MBI), or Stroke Impact Scale-mobility (SIS-mobility).<br>QoL: EuroQol-5 Dimensions (EQ-5D), Stroke Specific Quality of Life Scale (SS-QOL), or Stroke Impact Scale-emotion (SIS-emotion).					For non-RCTs: low external validity was observed where the source population was not adequately reported.<br>None of the studies reported sufficient power to detect treatment effects at a significance level.
**Sarfo et al. (2018)[19]**	Primary outcomes: Motor function (mobility, upper limb function, ankle disability)	Barthel Index scale, the Berg Balance Scale, the Functional Independence Measure scale, the Fugl-Meyer Extremity Test, the Wolf Motor Function Test, the Timed Up and Go Test, the Nine-Hole Peg Test, Action Research Arm Test, the walking speed and Ashworth Scale,	n/r	Descriptive synthesis	n/r	n/r	n/r

		health-related quality of life (e.g., EuroQol-5 Dimension, Short-Form Health Survey), satisfaction (e.g., Satisfaction with Stroke Care Questionnaire, satisfaction questionnaire).					
**Sharififar et al. (2023)[20]**	Primary outcome:<br>1-Body function: hand function, spasticity, ROM, mobility, upper limb function, quality of life, balance.<br>2- Activities of daily living.<br><br>Secondary outcomes:<br>1-Adherence<br>2-patient satisfaction	Body function:<br>FMA<br>WMFT<br><br>Activity:<br>BI<br><br>Adherence and patient satisfaction:<br>Self-reported records of adherence.<br>Satisfaction survey scores.	n/r	descriptive and meta-analysis.<br><br>Meta-analysis based on random effect model with 95% CI.	**Telerehabilitation vs. Control group:** post-treatment<br><br>Body function:<br>FMA: MD: 3.32 (0.90 - 5.74) I^2= 29%<br>WMFT: MD: 1.69 (0.21 - 3.17) I^2= 93%<br><br>Activity:<br>BI: MD: 4.18 (1.79 - 6.57) I^2= 16%<br><br>Adherence and patient satisfaction:<br>Self-reported records of adherence. n/r<br><br>Satisfaction survey scores. n/r	PEDro checklist	Most of the studies were characterized as " good" to "excellent" (PEDro score 6,6± 2.3 points) indicating relatively lower levels of internal validity and interpretability.
**Schroder et al. (2018)[21]**	Primary outcomes:<br>Balance<br>Walking function and mobility<br><br>Secondary outcomes:<br>Cost-benefit<br>Adherence to home-based therapy	Balance:<br>Berg Balance Scale (BBS)<br><br>Performance- Oriented mobility assessment balance subscale (POMA-B)<br><br>Walking function and mobility: POMA-G; SUS; TUG; 10mWT; SUE; SAE.	n/r	A descriptive synthesis	n/r	PEDro scale	one is of good quality; one of fair quality and two present a high risk of bias.
**Saragih et al. (2022)[22]**	Primary outcomes:<br>Independence in ADLs.<br>Balance.	Independence in ADLs:<br>Modified Barthel Index the Barthel Index<br>Balance:<br>Berg Balance Scale	Range from 4 weeks, 3 months, and 6 months.	Meta-analysis<br><br>Based on the Random-effect model	Independence in ADLs:<br>Modified Barthel Index; the Barthel Index: SMD:0.45 (0.12 to 0.78) I^2= 21.51%<br><br>Balance:	Cochrane risk of bias tool for randomized controlled trials (ROB-2)	All the included studies had a low risk of bias.

					Berg Balance Scale: SMD:0.03 (-0.38 to 0.45) I^2= 0%		
**Su et al. (2023)[23]**	Primary outcome: Balance	1- Berg Balance Scale (BBS)<br>2-Timed Up & Go Test (TUG)<br>3- the Spanish version of the trunk impairment scale 2.0 (S-TIS 2.0)<br>4- the Spanish version of the function in sitting test(S-FIST)<br>5- the Spanish version of the postural assessment scale for stroke patients (S-PASS)<br>6- Fugl-Meyer assessment (FMA)<br>7- Activities-specific balance confidence scale (ABC)<br>8- Tinetti performance-oriented mobility assessment -Balance (POMA-Balance)<br>9-Trunk impairment scale (TIS).	n/r	Meta-analysis. Based on the Random-effect model	**Telerehabilitation vs. conventional rehabilitation: Post-intervention:**<br><br>BBS: MD=2.80 (0.61 to 4.98) I^2= 51.90%<br><br>TUG: MD= -4.59 (-5.93 to -3.25) I^2= 0%<br><br>S-TIS 2.0: MD= 1.37 (-1.85 to 4.60) I^2= 65.61%<br><br>S-PASS: MD= -0.12 (-3.56 to 3.32) I^2= 1.88%<br><br>S-FIST: MD= 2.24 (-0.96 to 5.44) I^2= 0%<br><br>TIS: MD= -2.14 (-6.91 to 2.63) I^2= n/r<br><br>POMA: MD= 2.50 (0.39 to 4.61) I^2= n/r<br><br>ABC: MD= 3.97 (-9.31 to 17.25) I^2= n/r<br><br>FMA: MD= 8.12 (6.35 to 9.88) I^2= 0%	Cochrane Risk of Bias tool	Random sequence generation and allocation concealment were clear in all studies except 1.<br><br>Blinding: studies reported blinding except for 2 studies and 1 study indicated it was difficult to blind participants due to the nature of the intervention.<br><br>incomplete data outcome: most were unclear except for 2 studies.<br><br>4 studies had reporting bias.
**Szeto et al. (2023)[24]**	Motor paresis.<br>Adherence to exercise.<br>ADLs.<br>QoL.	Motor paresis: 10MWT comfort; 10MWT fast; 6MWT; TUG; MMT; Purdue pegboard Test (PPT); Modified Functional ambulatory category (MFAC); Wolf Motor function test (WMFT).<br>Adherence to exercise: ambulation (min/day;	n/r	Descriptive synthesis	n/r	Cochrane Risk of Bias tool and the modified Downs and Black checklist.	For RCTs: Random sequence generation was identified in 7 RCTs.<br>Allocation concealment was explained in 5 RCTs.<br>Blinding of outcome assessments was clear in 7 RCTs.<br>A low risk of attrition bias was demonstrated in 6 RCTs.<br>Only 3 studies have a low risk of reporting bias.

		sitting time(hours/day);<br>adherence VAS; % of HEP done/day.<br>ADLs: BI; mBI ;FIM; iADL scale.<br>QoL: n/r					In every RCT, participants were not blinded to the intervention group which led to a high risk of performance bias.
**Tchero et al. (2018)[25]**	Primary outcomes:<br>1- Activities of daily living and balance function.<br>2-Motor function<br>3- patients' quality of life.<br>Secondary outcomes:<br>1-Satisfaction with care<br>2-Cost effectiveness	1- Activities of daily living and balance function:<br>Barthel Index<br>Berg Balance Scale<br><br>2-Motor function:<br>Fugi-Meyer Upper Extremity<br>Action Research Arm Test<br>Stroke Impact Scale-Mobility Subscale<br><br>3- patients' quality of life:<br>Short-Form (SF-36) emotional role limitation.<br>2 different versions of the Functional Independence Measure: Self-administered and telephone versions.<br><br>4- Satisfaction with care:<br>Modified satisfaction questionnaire.	4-24 weeks	Both descriptive and meta-analysis.<br><br>Analysis was done first under the fixed-effect model for assuming homogeneity.<br>In the case of heterogeneity, we shifted to a random-effects model.	Telerehabilitation vs. Control group:<br>1- Activities of daily living and balance function:<br>BI: SMD: -0.05 (-0.18 to 0.08) I^2= 0%<br>BBS: SMD: -0.04 (-0.34 to 0.26) I^2= 0%<br><br>2-Motor function:<br>FMA-UE: SMD: 0,50 (-0.09 to 1.09) I^2= 0%<br>ARAT: SMD: -0,06 (-0.46 to 0.33) I^2= 0%<br>SIS (Mobility): SMD: 0.018 (-0.13 to 0.48) I^2= 0%<br><br>3- patients' quality of life:<br>Short-Form (SF-36) emotional role limitation: MD: 7.9 (0.1 to 15.7) I^2= n/r	Cochrane Risk of Bias tool	Allocation concealment was adequate.<br>Random sequence generation, blinding of outcome assessors, and reducing the risk of attrition bias were reported adequately.
**Toh et al. (2022)[26]**	Motor recovery of hemiparetic UL.	ARAT; FM; Chedoke Arm and Hand Inventory (CAHAI); WMFT, adherence rate;<br>ABILHAND Scale;<br>Ashworth scale; BI;<br>Modified Rankin Scale(MRS); 9-HPT;<br>Motor Activity Log(MAL);	n/r	Descriptive synthesis and Meta-analysis<br><br>Fixed or random effect model based on heterogeneity	**Home-based UL intervention vs. clinic-based therapy:** (for only one study)<br>SMD: 0.04 (-0.32 to 0.39) I^2= n/r<br>**Home-based UL intervention vs. "no technology" intervention:**<br>After treatment: SMD: 0.08 (-0.22 to 0.37) I^2= 25%	PEDro Scale.	All selected studies were rated as fair to high quality with 6.6 ± 1.2

		Nottingham extended activities of daily living;<br>BBT.			At follow-up: SMD: -0.10 (-0.36 to 0.15) I^2= 0%<br>**Home-based UL intervention vs. no intervention:** (for only one study)<br>After the treatment: SMD: 0.33(-0.60 to 1.26) I^2= n/r		
**Tarihoran et al. (2023)[27]**	Primary outcomes:<br>Ability to perform ADL.<br><br>Ability to maintain balance.	Ability to perform ADL: Modified Barthel Index<br><br>Ability to maintain balance:<br>The Berg Balance Scale	Range from 6 months to 24 months	Meta-analysis<br><br>Based on the Random-effect model	Ability to perform ADL:<br>Modified Barthel Index: SMD:0.57 (0.13 to 1.01) I^2= 71.99%<br><br>Ability to maintain balance:<br>The Berg Balance Scale:<br>SMD:1.96 (1.27 to 2.66) I^2= 66.49%	Version 2 of the Cochrane risk of bias tool for RCTs (ROB 2)	A potentially high risk of bias in the randomization process because of 1) lack of concealment of treatment and lack of blinding to treatment for a therapist and an assessor.
**Zhou et al. (2018)[28]**	Physical function:<br>Active and passive action of arm and hand.<br>ADL.<br>Quality of life.<br>Walking<br>UL function; hand function and muscle strength.	Physical function:<br>ADL: Instrumental Activities of Daily Living Scale.<br>Quality of life: Stroke Specific Quality of Life Scale.<br>Walking: 10-M walking test.<br>UL function; hand function and muscle strength: Fugl-Meyer Motor Assessment; Nine Hole Peg Test; Box & Block Test.	n/r	Descriptive synthesis	n/r	Australian Evidence-Based Health Care Centre.	No detailed description of randomization and allocation concealment.<br>participants not blind to treatment assignment.<br>It is not clear whether those delivering treatment were blind to treatment assignment.<br>Intention to treat analysis (ITT) was not performed.

Table S5/C: Details of interventions among included reviews

Review	Intervention group	Telerehabilitation approach	Frequency	Session duration	Intervention Period	Intervention provider	setting	Control group
**Appleby et al. (2019)[1]**	Customized software for tracking finger and wrist movement, neuromuscular stimulation, functional mobility exercises, ankle exercises, educational sessions, mobility and posture exercises, 3D videos exercise in a virtual home environment, balance training exercises, VR exercises.	Videoconferencing for instructions and communication (N=8).<br><br>3D motion equipment and software to generate virtual representation of participants movements (N=3).<br><br>Combined Videoconferencing with biofeedback and physiological data from participants (N=1).	a daily session to Three times per week	n/r	From 10 days to 12 weeks	n/r	n/r	n/r
**Alayat et al. (2022)[2]**	Telerehabilitation in conjunction with conventional rehabilitation (N= 6).<br><br>Only telerehabiltation (N=8)	Video and audio equipment for videoconferencing capabilities were the most common.<br><br>Also, telephone, platform monitoring, and text messages.	The range across studies from two to 9 sessions per week.	The range across studies from 15 min to 60 min	From 3 weeks to 6 months.	Therapist (N= 8); n/r (N=6)	n/r	Conventional rehabilitation(N=9)<br><br>Virtual reality- based training+ conventional rehabilitation(N=1)<br><br>Balance training(N=4)
**Bok et al. (2023)[3]**	Virtual reality (interactive games or virtual exercises) (N=3); games (piano, fishing, sports games) (N=3); robot-assisted devices (facilitates movement of arm, wrist, and hands to improve the range of motion) (N= 4).	Virtual reality (N=3); games (N=3); robot-assisted devices (N= 4).	The range from one time to 5 times per week	The range from 20 min to 3 hours.	The range between 3 and 10 weeks	n/r	Home-based	n/r

**Chen et al. (2015)[4]**	1- Virtual reality based (VR)-based rehabilitation training (N=6).<br><br>2- Strengthened care or adaptive strategies, functional exercises, educational videos, online chat sessions, balance training, instructed exercises via telephone (message, chat, call), or the Internet (N=5).	Telephone calls (N= 4).<br>Videoconferencing (N= 1)<br>Educational Videos (N= 1).<br>Digital Video disk (N= 1).<br>Desktop Videophone (N=1)<br>Virtual reality systems (N= 1)<br>n/r (2)	1 hour a day, 5 days/ week(N=1), 45-50 min, 3 times/week(N=2), n/r (N=8).	1 hour a day (N=1), 45-50 min, (N=2), n/r (N=8).	Range: 4-20 weeks (N=11).	n/r	n/r	Conventional rehabilitation (standard care, routine rehabilitation care, in-person rehab, usual care, hospital care, conventional balance training).
**Coupar et al. (2012)[5]**	Virtual reality and telerehabilitation training consisted of different virtual tasks, comprising several arm movements.	Virtual reality with Videoconferencing system (N=2).	5 times per week.	1 hour	1 month.	Therapist (N=2)	home-based	Usual care (N=1); same therapy in hospital (N=1)
**Deshmukh and Madhavan (2023)[6]**	1- intervention in acute phase: electromyography-triggered neuromuscular stimulation (ETNS) paired with exercises (n=1) and conventional physical therapy (PT) exercises (n=1).<br><br>2- intervention in chronic phase: a virtual reality program for balance and posture (n=3); an ankle movement program (n=1).<br><br>3- two-way video conferencing in both control as well as intervention group(n=1).	standard videoconferencing platforms (e.g. Skype), custom-designed platforms, or virtual reality.	Range from 2-3 times/ week.	40 to 60 min in (n=4); N/R(n=2)	4-12 weeks	n/r	n/r	Conventional physical therapy(n=2); in-person delivery of the same protocol used via telerehabilitation(n=2); or rehabilitation guidance via telephone(n=1).<br>Both groups receive different complexities of the same movement protocol(n=1)
**Everard et al. (2021)[7]**	Technologically assisted self-rehabilitation.	Tablets; Computers; Non-immersive VR; Video instructions.	n/r	n/r[9]	Ranging from 2 weeks to 1 year.	n/r	Home-based	Conventional rehabilitation

**Hao et al. (2023)[8]**	Microsoft Kinetic was the most commonly used VR system (N=4)<br><br>Integrated virtual reality-based telerehabilitation system (N=3)<br><br>VRRS.net system with Wireless Sensor Network system (N=1)	Synchronous (N=6)<br><br>Asynchronous (N=3)	Ranging from 2 to 5 days/ per week.	ranging from 15 min to 1 hour across studies.	from 3 to 12 weeks	Physical therapist	At home (N=5). Long-term-care facility(N=1). Community support group (N=1). Hospital room, or a simulated home room (N=2).	Conventional in-person rehabilitation with the same amount of intervention time (active control) (N=7). Usual care without additional rehabilitation (passive control) (N=2).
**Hwang et al. (2021)[9]**	Instructions for the exercises and adaptive strategies(N=2). Discussion session involving stroke-related issues, problem-solving and goal-setting skills, and Exercise session involving land-based exercise(N=1).	Telephone & Messaging (Sync+Async)(N=2); Web-based(Video conferencing) (Sync)(N=1).	2-3 times/ week.	2h(N=2)	9 weeks to 3 months	n/r	Home-based(N=2); n/r(N=1)	Usual care (N=2); waiting list (N=1)
**Johansson and Wild (2011)[10]**	Distant care program (N=1).<br><br>Problem-solving skills by telephone (N=1).<br><br>Motor arm telerehabilitation intervention (N=2).	Telephone consulting (N= 2); Virtual environment-based motor telerehabilitation and video consulting system (N=1); a portable device allows two-way video consulting (N=1).	one-hour session, 5 days per week (N=1); n/r(N=3)	one hour	Range: one month to 24 weeks (N=3); n/r(N=1).	telehealth nurses(N=1)	Home-based	n/r
**Lazem et al. (2023)[11]**	Telerehabilitation exercises found in eligible studies included non-immersive VR; semi-immersive VR and augmented reality (AR).	VR exercises were delivered by a variety of elements, such as computers with monitors, an eye movement controller, a joystick, a Logitech trackpad, a Microsoft Kinect V2 RGB-	The range is from two sessions per week to daily sessions.	ranged from 20 minutes to 120 minutes per session	Ranging from Three weeks to eight weeks	Therapist (N= 3).	At home (N=8); Domiciliary (N=1).	In-clinic rehabilitation or home rehabilitation.

		D camera, software platforms, and gloves equipped with bend sensors that could be controlled remotely through the internet.						
**Laver et al. (2020)[12]**	(n=8) interventions that included goal setting, education about secondary prevention, family therapy, and case management to enhance care and well-being after discharge. (n=6) Customised computer-based training programs to ↑ UL function. (n=4) customized telerehabilitation systems and communication between the participants and the therapist to ↑ balance &mobility. (n=1) exercises delivered remotely+ electrical stimulation with aim ↑ of limb function, mobility, and balance. (n=1) a combination of occupational therapy & physiotherapy to provide rehabilitation that often focuses on remediation of impaired limbs. (n=2) speech & language therapy for people with aphasia.	Telephone (n= 8), Videoconferencing hardware & software (n= 10), Desktop videophones (n=1), Some studies used a combination of technologies: (n=1) used a combination of telephone calls, an in-home messaging device, and video recordings. (n=1) used a combination of email, an online chat program, and an online resource room (a virtual online library) established for caregivers of stroke survivors.	2- 3 times/week.	10- 70 min	10 days to 3 months	Clinicians (medical practitioner, nurse and family therapist), stroke nurses, therapist.	1-participants own home (n=20). 2-Local health centre or at the study site (n=2) (participants not preferred home-based telerehabilitation.	1-In-person rehabilitation(n=9). 2-No rehabilitation- or usual care (n=10) 3- Different models of telerehabilitation (n=2). 4- three-arm study which compared telerehabilitation with both in-person intervention and no intervention (usual care).
**Lombardo & Islam (2023)[13]**	STeleR home-based training in exercises and adaptive strategies(N=1).	Voice calls and In-Home Messaging Device (IHMD).	3 × home televisits, 5 telephone calls, daily in-home	n/r	3 months	Physiotherapist or occupational therapist.	Home-based	Usual care.

			messaging device.					
**Nascimento et al. (2022)[14]**	Virtual reality task-oriented training (N=1); Virtual reality training (N=2).	Virtual reality (N=3)	5 times/week.	40-20 min	Range from 4 to 13 weeks.	Physical therapist or occupational therapist(N=3)	Home-based	Clinic-based exercises.
**Ostrowaska et al. (2021)[15]**	1- Home-based telerehabilitation (combined with educational module): (supervised and unsupervised sessions): Upper extremity task-specific training manual and accelerated Skill Acquisition Program (N= 1).<br><br>2- Home remote rehabilitation based on collaborative care model (N= 1).<br><br>3- Tele system based on games installed on smartphones (N=1).	1- Internet-enabled computer with table, chair, and 12 gaming input devices (N= 1).<br><br>2- Internet-based TCMeeting, Video conferencing system (N= 1).<br><br>3- Inertial motion sensors (IMUs) and cloud databases (N= 1).	n/r	Range: 30-70 min (N=2), N/R (N= 1)	Range: 4-6 weeks (N=2), N/R (N= 1)	1- The collaborative care team consisting of neurologists, nurses, rehabilitation therapists, counsellors, and caregivers (N= 1).<br><br>2-n/r (N= 2)	Home-based	1- therapy in an in-patient setting N= 1.)<br>2-Routine rehabilitation and nursing measures (N= 1).<br>3- standard rehabilitation treatment at the hospital (N= 1).
**Qin et al. (2022)[16]**	Home-based tele-supervising rehabilitation including physical exercises with ADL training and ETNS therapy (N=2).<br>Family-led, trained caregiver delivered home-based rehabilitation intervention(N=1).<br>(STeleR) intervention included home televisits and telephone intervention calls combined with usual care(N=1).<br>Progressive rehabilitation exercises including exercise training and	N/R(N=3); Telephone calls (N=1); Videoconferencing (N=1).	n/r	n/r	n/r	Professionals (N=3); tele therapist(N=2)	Home based(N=5)	Institutional-based intervention (N=4); Usual care (N=1).

	training of functional activities were prescribed by a tele-therapist and performed by patients themselves (N=1)							
**Rintala et al. (2019)[17]**	Individualized physical exercises for improving mobility, strength, balance, walking, and stretching (N=4).<br><br>Only UE exercises performed in a virtual environment at home(N=3); Balance and body position exercises (N=1) or use of orthosis (N=1).<br><br>LE exercises such as gait-related exercises with balance and coordination exercises (N=2).<br><br>Exercises focused on promoting physical activity (N=2).	Online Video monitoring (N=5) 3 studies of these 5 used their technologies alongside online video monitoring such as telephone calls and messaging, gamification or accelerometer<br>Telephone calls (N=3).<br>Exercise Video through electronic tablet (N=1).<br>Virtual training program(N=2).<br>Digital versatile disc (DVD)(N=1).<br>Internet along with gamification(N=1).	Range from 1 time to 6 times per week(N=10); n/r (N=3).	Range from 20 min to 60 min.	12 weeks (N=1); n/r(N=12).	Physiotherapist or occupational therapist or both (N=12); nurse (N=1).	Home-based (N=8). Clinic based (N=1); n/r (N=4)	Usual care (N=8); Home-based exercise program for LL (N1); similar intervention group but without technology(N=2); home stretching program with instructions(N=1). Conventional Exercise(N=1).
**Rintala et al. (2023)[18]**	The content of mHealth apps is classified as gaming (Six exergames: (1)anteroposterior stability limits, (2)mediolateral stability limits, (3) sit-to-stand transfer, (4) standing, (5) reactive balance, and(6)postural control (IMU at lumbar level and anterior thigh paretic side)+usual care(N=1); to improve strength, endurance, ROM, control, speed, and	Smartphone-based mHealth(N=7).	Range from 2 to 9 times per week (N=6);<br>n/r (N=1).	Range from 30 min to 1 hour (N=5); n/r(N=2).	Range from 2 to 12 weeks(N=7)	n/r	hospital setting(N=2); home(N=4); combination of inpatient and home setting(N=1)	Usual care(N=4); No rehabilitation (N=2). Trunk, strengthening exercises, gait training, and occupational therapy (1)

	accuracy of UE (smartphone attached to patients' arm)+usual care(N=1); supervised aerobic, task-oriented training, balance and stretching exercises(N=1); fingers games(N=1); exercise prescription e.g. mobility, UL and LL strengthening, sitting, standing balance, walking endurance and core exercise(N=3).<br><br>The experimental group received usual care consisting of (supervised aerobic exercises, task-oriented training, balance training, muscle training, gait, and posture training or stretching exercises) with the apps(N=5).							
**Sarfo et al. (2018)[19]**	Physical exercises and electromyography-triggered neuromuscular stimulation (ETNS) (N=1).<br>VR rehabilitation program (N=4).<br>Behavioral change intervention (STARFISH) (N=1).<br>Caregiver-mediated training program with e-health support (N=1).<br>Robotic-Assisted Therapy+ HEP (N=1).<br>Functionality-based exercises and adaptive strategies (STeleR) (N=1).	Phone-based (N= 5)<br>Computer based (N= 6)<br>Tablet based (N= 1)<br>Video based (N= 4)<br>Not Clear (N= 1)<br>tele-monitoring at kiosks(N=1)	n/r	n/r	Ranged between 2 weeks and 24 weeks.	n/r	n/r	Conventional rehabilitation or no rehabilitation

	Home exercise program (motor learning program+Kiosk) (N=1).<br>Physical exercise telerehabilitation program (the 3D animation exercise Video +3D interactive games) (N=2).<br>Video-based physical therapy program (N=1).<br>Home-based physical exercise program, interventional care (audiovisual materials) (N=1).<br>Complex/ Simple movements training (N=1).<br>Telerehabilitation program (motor tasks and Video consulting system) (N=1).<br>Home Care Activity Desk training system exercise (N=1).<br>Computerized tracking training (N=1).							
**Sharififar et al. (2023)[20]**	Practice with objects of different shapes(N=1);<br>ETNS+Bobath PNF sit to stand and walking(N=1);<br>Televisit at home to modify home environment and communicate adaptive techniques(N=1);<br>Telegaming and self-managed video-game motor practice(N=1);<br>Game-based therapy(N=1);<br>Video-based telerehabilitation with face-to-face support detailing six	Home-care-activity Desk (HCAD)(N=1)<br>Home-based phone calls(N=2)<br>Monitoring kiosks distributed through the community(N=1)<br>Home-based DVD records(N=1)<br>Home-based video conferencing(N=2)	Two to five times/week(N=4);<br>N/R(N=3).	Ranged from 30 min to 70 min.	4-24 weeks	Physical or occupational therapist.	home based.	Conventional face-to-face therapy.

	domains of positioning, handling, bed mobility, PROM, strengthening, and stretching of UE and LE(N=1). CIMT practiced telerehabilitation at home Face to face with the therapist(N=1).							
**Schroder et al. (2018)[21]**	VR system: a commercially available gaming device to provide VR such as Microsoft Kinect uses a camera for full-body capture to interact with the VR(N=1) A system similar to the Wii balance board (N=1) and as a force plate (balance trainer) (N=1). A touch screen to allow interaction with the VR (N=1).	Video conferences (N=3); Virtual reality (4)	Ranging from 3 to 5 times per week.	ranging from 15min to 70 min.	Ranging from 2 to 4 weeks	n/r	Home base	Same intervention in clinical setting (N=1)<br><br>Standing balance training in clinic (N=1)<br><br>Conventional therapy in clinic (N=1)<br><br>Balance training with standing frame without VR in clinic(N=1)
**Saragih et al. (2022)[22]**	Telerehabilitation interventions covered a range of educational topics, including physical exercise and promoting health behaviors (N=6).	Telephone calls (N=1); mHealth apps(N=1); Video Conference (N=1); 3D animation Ex's Videos(N=1); N/R (N=2)	Range from 2 to 3 days per week.	Range from 20 min to 1 hour.	Range from 1 to 6 months.	Therapists (N=3); Trained nurse(N=1); n/r (N=2)	Hospital (N=1); home (N=1); Community (N=2); Neurological rehabilitation centre (N=1); Long Term Care facility(N=1);	Usual care (N=2); n/r (N=2); Conventional rehabilitation programmes as usual (N=2).
**Su et al. (2023)[23]**	Telerehabilitation interventions were varied (commercial video games, Nintendo, Microsoft Kinect, or customised devices (platforms not devices). (health education, physical strength training, balance	(Telephone, Videoconferencing, APP monitoring)	Range from 2 days to 5 days/week (N=9)	Range:20 min to 1 hour (N=9).	Range: 2 weeks to 3 months (N=9).	Neuro physiotherapist (N=1), n/r(N=8)	n/r	Conventional rehabilitation

	training, breathing training, walking training, stair climbing, sit-to-stand exercise and walking, core stability exercises, dance exercises, upper limb training, lower limb training).							
**Szeto et al. (2023)[24]**	Therapy apps; rehab videos; reminders and rehab videos with reminders.	mobile apps.	n/r	n/r	n/r	n/r	n/r	Conventional rehabilitation(N=4); Unclear (N=3); Exercise booklet (N=3).
**Tchero et al. (2018)[25]**	n/r	Telephone calls. Videoconferencing. Educational Videos. Web-based chat. Virtual reality systems.	n/r	n/r	n/r	n/r	n/r	Usual rehabilitation
**Toh et al. (2022)[26]**	Home-based VR (N=4) Home-based telerehabilitation(N=2) Home-based iPad(N=1) Home-based Video exercise(N=1) the content of each intervention is not reported in this review.	VR(N=4); iPad(N=1); Video (N=1); n/r (N=2)	Range from 3 times to 5 times per week	n/r	Range from 3 weeks to 12 weeks across studies.	n/r	Home-based (N=8).	Clinic-based therapy (N=3); No intervention(N=1);"no technology intervention" (N=4).
**Tarihoran et al. (2023)[27]**	Intervention covered a range of educational topics, including physical exercise and home care of stroke survivors	Videoconferencing (N=9)	Range from 2 to 10 times per week.	Range from 30 min to 1hour.	Range from 6 weeks to 3 months	Occupational and physical therapists, trained research assistants, neurologists, and nurses.	Home (N= 3); home and outpatient rehabilitation department (N= 3); Community (N=1); Medical centre(N=1); n/r (N=1)	Conventional rehabilitation (N=3) or usual care(N=3). Physical exercises and ETNS in outpatient rehab department (N=1); Telephone follow-up(N=1); waiting list control group (N=1).
**Zhou et al. (2018)[28]**	(STARFISH)app: used colored fish to represent a participant and growth of	Smartphone-based application (N=1); Tablet PC used for the application(N=1).	6 day/week. Session(N=1), n/r (N=1)	31 min(N=1); N/R (N=1)	The range between 4 to 6 weeks.	n/r	n/r	Usual care (N=1); no training (N=1)

	the fish to represent individualized step(N=1); the training session consisted of five programs (stretching, flexion, extension, opposition, and thumb abduction) (N=1).	

Virtual reality used for the experimental group in the primary studies among the included reviews				
Review	Primary studies	VR interventions	Mode of delivery	Stroke phase
**Bok et al. (2023)[3]**	Johnson et al. (2020) [29]	Virtual therapy VT games that target UE mobility:<br>1- Capture as many stars as you can<br>2- Reach and clap your hand over the balloons to pop them.<br>3- Reach with your hands to touch the green and blue balls and avoid the red balls.<br>4- Get as many apples into the pipes.<br>Equipment: A Microsoft Xbox Kinect V.2 (Microsoft, Redmond, Washington, USA) and a laptop.<br>Duration: VT session will last approximately 45–60min, twice weekly, for 8 weeks	Non-immersive VR	At least 3 months post-stroke
	Standen et al. (2017)[30]	The virtual glove+ 3 games:<br>1- Spacerace required pronation and supination of the hand to guide a spacecraft through obstacles.<br>2- Sponge ball requires the user to open their fist and extend their fingers to release a ball to hit a target.<br>3- Balloonpop required a balloon to be grasped and popped by moving it to a pin protruding from the virtual floor.<br>Duration: a maximum of twenty minutes, three times a day, for eight weeks.	Semi-immersive VR	n/r
	Lloréns et al. (2015)[31]	Home-based telerehabilitation system: balance exercises in empty scenario environment.<br>Equipment: television, computer or laptop, and Microsoft Kinect.<br>Duration: Twenty sessions (45-minute, conducted 3 times a week)	Non-immersive VR	Chronic

**Coupar et al. (2012)[5]**	Piron et al. (2008)[32]	Virtual tasks are supervised via videoconferencing.<br><br>Equipment: a 3D motion tracking system (Polhemus 3Space Fastrak, Vermont, US) that recorded the patient's arm movements, and a computer screen.<br><br>Duration: one hour of rehabilitation daily for one month.	Non-immersive VR	Chronic
	Piron et al. (2009)[33]	Virtual tasks are supervised via videoconferencing.<br><br>Equipment: a 3D motion tracking system (Polhemus 3Space Fastrak, Vermont, US) that recorded the patient's arm movements, and a computer screen.<br><br>Duration: 1 hour a day, 5 days a week for one month.	Non-immersive VR	Chronic
**Chen et al. (2015)[4]**	Lloréns et al. (2015)[31]<br><br>Piron et al. (2008)[32]<br><br>Piron et al. (2009)[33]	Mentioned above		
	Lin et al. (2014)[34]	The tele-balance training: 10 minutes of standing exercise according to 3D animation exercise videos and 10 minutes of 3D interactive games.<br><br>Equipment: personal computer and webcam.<br><br>Duration: Three sessions per week and for four weeks.	Non-immersive VR	Chronic
**Deshmukh and Madhavan (2023)[6]**	Chen et al. (2021)[35]	The game-based telerehabilitation programs: video games focusing on participants' balance, weight-bearing, strength, weight shifting, and walking.<br><br>Equipment: a screen and a Kinect sensor.<br><br>Duration: Three sessions per week and for four weeks.	Non-immersive VR	Chronic
	Lloréns et al. (2015)[31]			

	Lin et al. (2014)[34]	Mentioned above		
**Hao et al. (2023)[8]**	Chen et al. (2021)[35]<br><br>Lloréns et al. (2015)[31]<br><br>Lin et al. (2014)[34]<br><br>Piron et al. (2008)[32]<br><br>Piron et al. (2009)[33]<br><br>Johnson et al. (2020)[29]	Mentioned above		
	Jonsdottir et al. (2021)[36]	Phase 1: ClinicHEAD training consists of Motor, cognitive, and occupational exercises.<br><br>Phase 2: HomeHEAD training, followed the same exercises in ClinicHEAD training without supervision.<br><br>Equipment: Microsoft Kinect, television screen, and leap motion.<br><br>Duration: 45 min/5 days per week for 12 week	Non-immersive VR	Chronic
**Johansson and Wild (2011)[29]**	Piron et al. (2009)[33]	Mentioned above		
**Lazem et al. (2023)[11]**	Cramer et al. (2019)[37]	88 arm exercises (e.g. stretching, strengthening, and active range of motion)+ functional games involving: driving game, Carnival shooting game, Space Invaders game, Piano game, Slot machine game<br><br>Equipment: table, chair, and 12 gaming input devices, but no keyboard, as no computer operation was required by patients.<br><br>Duration: 70 min+10min break; 18 sessions supervised and 18 sessions unsupervised for 6 to 8 weeks.	Non-immersive VR	Subacute/ Chronic
	Adie et al. (2014)[38]	Nintendo Wii Sports games include tennis, golf, boxing, baseball, and bowling activities.	Non-immersive VR	Chronic

		Equipment: computer screen and camera.<br>Duration: 45 minutes for 6 weeks		
	Ballester et al. (2017)[39]	goal-directed grasp action execution and motor imagery.<br>Equipment: pair of data gloves equipped with bend sensors, screen, camera.<br>Duration: 20 minutes per session, 5 days per week for 3 weeks.	Semi-immersive VR	Chronic
	Zondervan et al. (2016)[40]	MusicGlove therapy.<br>Equipment: MusicGlove device and a laptop.<br>Duration: 60 min per session, 3 sessions per week for 3 weeks.	Semi-immersive VR	Chronic
	Hernandez et al. (2022)[41]	Jintronix system for upper limb exercise involving Fish Frenzy, Catch-Carry-Drop, Kitchen, Pop Clap game activities.<br>Equipment: computer, large screen, and Microsoft Kinect.<br>Duration: 5 times a week for ≥20 minutes per session.	Non immersive VR	Chronic
	Lloréns et al. (2015)[31]<br>Piron et al. (2009)[33]	Mentioned above		
**Nascimento et al.(2022)[14]**	Piron et al. (2008)[32]<br>Piron et al. (2009)[33]	Mentioned above		

**Schroder et al. (2018)[21]**	Krpic et al. (2013)[42]	Screen-based (Panda 3D engine) balance training.<br><br>Equipment: computer, tablet or smartphone, balance trainer, and standing frame.<br><br>Duration: 15 min, 5 days per week for 3 weeks.	Non-immersive VR	Subacute/chronic
	Cikajlo et al. (2012)[43]	Screen-based (VRML 2.0) balance training.<br><br>Equipment: computer, tablet, or smartphone, balance trainer, and standing frame with tilt sensor.<br><br>Duration: 20 min, 5 days per week for 3 weeks.	Non-immersive VR	Subacute/chronic
	Lloréns et al. (2015)[31]<br><br>Lin et al. (2014)[34]	Mentioned above		
**Sarfo et al. (2018)[19]**	Choi et al. (2016)[44]	a mobile game-based upper extremity VR program:<br>1- "Honey Pot Guard" Game.<br>2- “Protect the Bunny" Game.<br>3- "Put out Fire" game.<br>4- "Flower Splash" game.<br><br>Equipment: accelerometer, smartphone, and a tablet PC.<br>Duration: 60 min,10 sessions of therapy, 5 days per week, for 2 weeks.	Non-immersive VR	n/r
	Lloréns et al. (2015)[31]<br><br>Lin et al. (2014)[34]<br><br>Piron et al. (2008)[32]<br><br>Piron et al. (2009)[33]	Mentioned above		

**Tchero et al. (2018)[25]**			Not reported
**Toh et al. (2022)[26]**	Adie et al. (2014)[38]<br>Ballester et al. (2017)[39]<br>Cramer et al. (2019)[37]<br>Piron et al. (2009)[33]<br>Standen et al. (2017)[30]<br>Zondervan et al. (2016)	Mentioned above	

References:

1. Appleby, E.; Gill, S.T.; Hayes, L.K.; Walker, T.L.; Walsh, M.; Kumar, S. Effectiveness of telerehabilitation in the management of adults with stroke: A systematic review. *PloS one* **2019**, *14*, e0225150, doi:doi:https://dx.doi.org/10.1371/journal.pone.0225150.
2. Alayat, M.; Almatrafi, N.; Almutairi, A.; El Fiky, A.; Elsodany, A. The Effectiveness of Telerehabilitation on Balance and Functional Mobility in Patients with Stroke: A Systematic Review and Meta-Analysis. *INTERNATIONAL JOURNAL OF TELEREHABILITATION* **2022**, *14*, doi:doi:10.5195/ijt.2022.6532.
3. Bok, S.; Song, Y.; Lim, A.; Jin, S.; Kim, N.; Ko, G. High-Tech Home-Based Rehabilitation after Stroke: A Systematic Review and Meta-Analysis. *JOURNAL OF CLINICAL MEDICINE* **2023**, *12*, doi:doi:10.3390/jcm12072668.
4. Chen, J.; Jin, W.; Zhang, X.-X.; Xu, W.; Liu, X.-N.; Ren, C.-C. Telerehabilitation Approaches for Stroke Patients: Systematic Review and Meta-analysis of Randomized Controlled Trials. *Journal of Stroke & Cerebrovascular Diseases* **2015**, *24*, 2660-2668, doi:doi:10.1016/j.jstrokecerebrovasdis.2015.09.014.
5. Coupar, F.; Pollock, A.; Legg, L.A.; Sackley, C.; Van Vliet, P. Home-based therapy programmes for upper limb functional recovery following stroke. *Cochrane Database of Systematic Reviews* **2012**.
6. Deshmukh, S.; Madhavan, S. Can post stroke walking improve via telerehabilitation? A systematic review in adults with stroke. *Frontiers in rehabilitation sciences* **2023**, *4*, 1154686, doi:doi:https://dx.doi.org/10.3389/fresc.2023.1154686.

7. Everard, G.; Luc, A.; Doumas, I.; Ajana, K.; Stoquart, G.; Edwards, M.; Lejeune, T. Self-Rehabilitation for Post-Stroke Motor Function and Activity-A Systematic Review and Meta-Analysis. *NEUROREHABILITATION AND NEURAL REPAIR* **2021**, *35*, 1043-1058, doi:doi:10.1177/15459683211048773.
8. Hao, J.; Pu, Y.; Chen, Z.; Siu, K.-C. Effects of virtual reality-based telerehabilitation for stroke patients: A systematic review and meta-analysis of randomized controlled trials. *Journal of Stroke and Cerebrovascular Diseases* **2023**, *32*, 106960.
9. Hwang, N.; Park, J.; Chang, M. Telehealth Interventions to Support Self-Management in Stroke Survivors: A Systematic Review. *HEALTHCARE* **2021**, *9*, doi:doi:10.3390/healthcare9040472.
10. Johansson, T.; Wild, C. Telerehabilitation in stroke care--a systematic review. *Journal of Telemedicine & Telecare* **2011**, *17*, 1-6, doi:doi:10.1258/jtt.2010.100105.
11. Lazem, H.; Hall, A.; Gomaa, Y.; Mansoubi, M.; Lamb, S.; Dawes, H. The Extent of Evidence Supporting the Effectiveness of Extended Reality Telerehabilitation on Different Qualitative and Quantitative Outcomes in Stroke Survivors: A Systematic Review. *International journal of environmental research and public health* **2023**, *20*, doi:doi:https://dx.doi.org/10.3390/ijerph20176630.
12. Laver, K.E.; Adey-Wakeling, Z.; Crotty, M.; Lannin, N.A.; George, S.; Sherrington, C. Telerehabilitation services for stroke. *Cochrane Database of Systematic Reviews* **2020**.
13. Lombardo, C.; Islam, M.S. Stroke survivors' acceptance and satisfaction of telerehabilitation delivery of physiotherapy services: a systematic review. *Physical Therapy Reviews* **2023**, *28*, 261-277.
14. Nascimento, L.R.; Rocha, R.J.; Boening, A.; Ferreira, G.P.; Perovano, M.C. Home-based exercises are as effective as equivalent doses of centre-based exercises for improving walking speed and balance after stroke: a systematic review. *Journal of physiotherapy* **2022**, *68*, 174-181.
15. Ostrowska, P.M.; Sliwinski, M.; Studnicki, R.; Hansdorfer-Korzon, R. Telerehabilitation of Post-Stroke Patients as a Therapeutic Solution in the Era of the Covid-19 Pandemic. *Healthcare (Basel, Switzerland)* **2021**, *9*, doi:doi:https://dx.doi.org/10.3390/healthcare9060654.
16. Qin, P.; Cai, C.; Chen, X.; Wei, X. Effect of home-based interventions on basic activities of daily living for patients who had a stroke: a systematic review with meta-analysis. *BMJ open* **2022**, *12*, e056045.
17. Rintala, A.; Paivarinne, V.; Hakala, S.; Paltamaa, J.; Heinonen, A.; Karvanen, J.; Sjogren, T. Effectiveness of Technology-Based Distance Physical Rehabilitation Interventions for Improving Physical Functioning in Stroke: A Systematic Review and Meta-analysis of Randomized Controlled Trials. *Archives of physical medicine and rehabilitation* **2019**, *100*, 1339-1358, doi:doi:https://dx.doi.org/10.1016/j.apmr.2018.11.007.
18. Rintala, A.; Kossi, O.; Bonnechere, B.; Evers, L.; Printemps, E.; Feys, P. Mobile health applications for improving physical function, physical activity, and quality of life in stroke survivors: a systematic review. *Disabil Rehabil* **2023**, *45*, 4001-4015, doi:10.1080/09638288.2022.2140844.
19. Sarfo, F.S.; Ulasavets, U.; Opare-Sem, O.K.; Ovbiagele, B. Tele-Rehabilitation after Stroke: An Updated Systematic Review of the Literature. *Journal of Stroke & Cerebrovascular Diseases* **2018**, *27*, 2306-2318, doi:doi:10.1016/j.jstrokecerebrovasdis.2018.05.013.

20. Sharififar, S.; Ghasemi, H.; Geis, C.; Azari, H.; Adkins, L.; Speight, B.; Vincent, H.K. Telerehabilitation service impact on physical function and adherence compared to face-to-face rehabilitation in patients with stroke: A systematic review and meta-analysis. *PM & R : the journal of injury, function, and rehabilitation* **2023**, doi:doi:https://dx.doi.org/10.1002/pmrj.12988.
21. Schroder, J.; van Criekinge, T.; Embrechts, E.; Celis, X.; Van Schuppen, J.; Truijen, S.; Saeys, W. Combining the benefits of tele-rehabilitation and virtual reality-based balance training: a systematic review on feasibility and effectiveness. *Disabil Rehabil Assist Technol* **2019**, *14*, 2-11, doi:10.1080/17483107.2018.1503738.
22. Saragih, I.D.; Tarihoran, D.E.T.A.U.; Batubara, S.O.; Tzeng, H.M.; Lin, C.J. Effects of telehealth interventions on performing activities of daily living and maintaining balance in stroke survivors: A systematic review and meta-analysis of randomised controlled studies. *Journal of Clinical Nursing (John Wiley & Sons, Inc.)* **2022**, *31*, 2678-2690, doi:doi:10.1111/jocn.16142.
23. Su, Z.; Guo, Z.; Wang, W.; Liu, Y.; Liu, Y.; Chen, W.; Zheng, M.; Michael, N.; Lu, S.; Wang, W.; et al. The effect of telerehabilitation on balance in stroke patients: is it more effective than the traditional rehabilitation model? A meta-analysis of randomized controlled trials published during the COVID-19 pandemic. *Frontiers in neurology* **2023**, *14*, 1156473, doi:doi:https://dx.doi.org/10.3389/fneur.2023.1156473.
24. Szeto, S.G.; Wan, H.; Alavinia, M.; Dukelow, S.; MacNeill, H. Effect of mobile application types on stroke rehabilitation: a systematic review. *J Neuroeng Rehabil* **2023**, *20*, 12, doi:10.1186/s12984-023-01124-9.
25. Tchero, H.; Teguo, M.T.; Lannuzel, A.; Rusch, E.; Tabue Teguo, M. Telerehabilitation for Stroke Survivors: Systematic Review and Meta-Analysis. *Journal of Medical Internet Research* **2018**, *20*, 80-80, doi:doi:10.2196/10867.
26. Toh, S.F.M.; Chia, P.F.; Fong, K.N.K. Effectiveness of home-based upper limb rehabilitation in stroke survivors: A systematic review and meta-analysis. *Front Neurol* **2022**, *13*, 964196, doi:10.3389/fneur.2022.964196.
27. Tarihoran, D.; Daryanti Saragih, I.; Saragih, I.S.; Tzeng, H.M. Effects of videoconferencing intervention on stroke survivors: A systematic review and meta-analysis of randomised controlled studies. *J Clin Nurs* **2023**, *32*, 5938-5947, doi:10.1111/jocn.16716.
28. Zhou, X.; Du, M.; Zhou, L. Use of mobile applications in post-stroke rehabilitation: a systematic review. *Top Stroke Rehabil* **2018**, 1-11, doi:10.1080/10749357.2018.1482446.
29. Johnson, L.; Bird, M.-L.; Muthalib, M.; Teo, W.-P. An Innovative STRoke Interactive Virtual thErapy (STRIVE) online platform for community-dwelling stroke survivors: A randomized controlled trial. *Archives of Physical Medicine and Rehabilitation* **2020**, *101*, 1131-1137.
30. Standen, P.; Threapleton, K.; Richardson, A.; Connell, L.; Brown, D.; Battersby, S.; Platts, F.; Burton, A. A low cost virtual reality system for home based rehabilitation of the arm following stroke: a randomised controlled feasibility trial. *Clinical rehabilitation* **2017**, *31*, 340-350.
31. Lloréns, R.; Noé, E.; Colomer, C.; Alcañiz, M. Effectiveness, usability, and cost-benefit of a virtual reality–based telerehabilitation program for balance recovery after stroke: A randomized controlled trial. *Archives of physical medicine and rehabilitation* **2015**, *96*, 418-425. e412.
32. Piron, L.; Turolla, A.; Tonin, P.; Piccione, F.; Lain, L.; Dam, M. Satisfaction with care in post-stroke patients undergoing a telerehabilitation programme at home. *Journal of telemedicine and telecare* **2008**, *14*, 257-260.
33. Piron, L.; Turolla, A.; Agostini, M.; Zucconi, C.; Cortese, F.; Zampolini, M.; Zannini, M.; Dam, M.; Ventura, L.; Battauz, M. Exercises for paretic upper limb after stroke: a combined virtual-reality and telemedicine approach. *Journal of rehabilitation medicine* **2009**, *41*, 1016-1020.

34. Lin, R.C.; Chiang, S.L.; Heitkemper, M.M.; Weng, S.M.; Lin, C.F.; Yang, F.C.; Lin, C.H. Effectiveness of early rehabilitation combined with virtual reality training on muscle strength, mood state, and functional status in patients with acute stroke: a randomized controlled trial. *Worldviews on Evidence-Based Nursing* **2020**, *17*, 158-167.
35. Chen, S.-C.; Lin, C.-H.; Su, S.-W.; Chang, Y.-T.; Lai, C.-H. Feasibility and effect of interactive telerehabilitation on balance in individuals with chronic stroke: a pilot study. *Journal of neuroengineering and rehabilitation* **2021**, *18*, 1-11.
36. Jonsdottir, J.; Baglio, F.; Gindri, P.; Isernia, S.; Castiglioni, C.; Gramigna, C.; Palumbo, G.; Pagliari, C.; Di Tella, S.; Perini, G. Virtual reality for motor and cognitive rehabilitation from clinic to home: a pilot feasibility and efficacy study for persons with chronic stroke. *Frontiers in Neurology* **2021**, *12*, 601131.
37. Cramer, S.C.; Dodakian, L.; Le, V.; See, J.; Augsburger, R.; McKenzie, A.; Zhou, R.J.; Chiu, N.L.; Heckhausen, J.; Cassidy, J.M. Efficacy of home-based telerehabilitation vs in-clinic therapy for adults after stroke: a randomized clinical trial. *JAMA neurology* **2019**, *76*, 1079-1087.
38. Adie, K.; Schofield, C.; Berrow, M.; Wingham, J.; Humfryes, J.; Pritchard, C.; James, M.; Allison, R. Does the use of Nintendo Wii SportsTM improve arm function? Trial of WiiTM in Stroke: a randomized controlled trial and economics analysis. *Clinical rehabilitation* **2017**, *31*, 173-185.
39. Ballester, B.R.; Nirme, J.; Camacho, I.; Duarte, E.; Rodríguez, S.; Cuxart, A.; Duff, A.; Verschure, P.F. Domiciliary VR-based therapy for functional recovery and cortical reorganization: randomized controlled trial in participants at the chronic stage post stroke. *JMIR serious games* **2017**, *5*, e6773.
40. Zondervan, D.K.; Friedman, N.; Chang, E.; Zhao, X.; Augsburger, R.; Reinkensmeyer, D.J.; Cramer, S.C. Home-based hand rehabilitation after chronic stroke: Randomized, controlled single-blind trial comparing the MusicGlove with a conventional exercise program. *Journal of rehabilitation research and development* **2016**, *53*, 457-472.
41. Hernandez, A.; Bubyr, L.; Archambault, P.S.; Higgins, J.; Levin, M.F.; Kairy, D. Virtual reality–based rehabilitation as a feasible and engaging tool for the management of chronic poststroke upper-extremity function recovery: Randomized controlled trial. *JMIR Serious Games* **2022**, *10*, e37506.
42. Krpic, A.; Savanovic, A.; Cikajlo, I. Telerehabilitation: remote multimedia-supported assistance and mobile monitoring of balance training outcomes can facilitate the clinical staff’s effort. *International journal of rehabilitation research* **2013**, *36*, 162-171.
43. Cikajlo, I.; Rudolf, M.; Goljar, N.; Burger, H.; Matjačić, Z. Telerehabilitation using virtual reality task can improve balance in patients with stroke. *Disability and rehabilitation* **2012**, *34*, 13-18.
44. Choi, Y.-H.; Ku, J.; Lim, H.; Kim, Y.H.; Paik, N.-J. Mobile game-based virtual reality rehabilitation program for upper limb dysfunction after ischemic stroke. *Restorative neurology and neuroscience* **2016**, *34*, 455-463.
