# Supplementary material for: Telerehabilitation and Its Impact Following Stroke: An Umbrella Review of Systematic Reviews
Source: J Clin Med. 2024 Dec 26;14(1):50. doi: 10.3390/jcm14010050 (PMC11721391; doi:10.3390/jcm14010050)
Supplement: Supplementary file 1 [file jcm-14-00050-s001.zip › Table S6 Methodological quality of SRs.pdf]

Table S6 JBI quality assessment of the included reviews

**Reviews**	Q1	Q2	Q3	Q4	Q5	Q6	Q7	Q8	Q9	Q10	Q11	Total (Yes)	Overall quality
Laver et al. 2020[1]	N	Y	Y	Y	Y	Y	U	Y	Y	Y	Y	9	High
Sharififar et al. 2023[2]	N	Y	Y	Y	Y	N	N	N	Y	Y	Y	7	Medium
Techero et al. 2018[3]	N	N	Y	Y	Y	Y	Y	N	N	N	Y	6	Medium
Ostrowska et al. 2021[4]	Y	Y	U	Y	N	N	N	Y	N	Y	N	5	Medium
Chen et al. 2015[5]	N	Y	Y	Y	Y	Y	N	N	Y	N	Y	7	Medium
Su et al. 2023[6]	Y	Y	Y	Y	Y	Y	Y	Y	N	N	Y	9	High
Appleby et al. 2019[7]	N	Y	Y	Y	Y	Y	Y	Y	N	Y	Y	9	High
Johansson and Wild 2011[8]	N	Y	U	Y	N	N	U	Y	N	N	Y	4	Low
Alayat et al. 2022[9]	N	Y	Y	Y	Y	Y	Y	Y	Y	N	Y	9	High
Deshmukh and Madhavan 2023[10]	N	Y	Y	Y	Y	Y	U	Y	N	N	Y	7	Medium
Sarfo et al. 2018[11]	N	Y	U	Y	N	Y	U	Y	N	N	Y	5	Medium
Schroder et al. 2018[12]	N	Y	Y	Y	Y	Y	Y	Y	N	Y	Y	9	High
Hao et al. 2023[13]	N	Y	Y	Y	Y	Y	U	Y	Y	Y	N	8	High
Lazem et al. 2023[14]	N	Y	Y	Y	Y	Y	Y	Y	Y	N	Y	9	High
Saragih et al. 2022[15]	N	Y	Y	Y	Y	Y	U	Y	Y	Y	Y	9	High
Bok et al. 2023[16]	Y	Y	Y	Y	Y	Y	U	Y	Y	N	N	8	High
Tarihoran et al. 2023[17]	N	Y	Y	Y	Y	Y	U	Y	Y	Y	Y	9	High

Rintala et al. 2019[18]	N	Y	Y	Y	Y	Y	N	Y	Y	N	Y	8	High
Zhou et al. 2018[19]	N	Y	Y	Y	Y	Y	Y	Y	N	Y	Y	9	High
Rintala et al. 2023[20]	N	Y	U	Y	Y	Y	N	Y	N	N	Y	6	Medium
Szeto et al. 2023[21]	N	Y	Y	Y	Y	N	N	Y	N	N	Y	6	Medium
Qin et al. 2022[22]	N	Y	Y	Y	Y	Y	Y	N	N	Y	Y	8	High
Toh et al. 2022[23]	Y	Y	Y	Y	Y	Y	N	Y	Y	Y	Y	10	High
Nascimento et al. 2022[24]	Y	Y	Y	Y	Y	N	U	Y	N	Y	Y	8	High
Coupar et al. 2012[25]	N	Y	Y	Y	U	Y	Y	Y	N	Y	Y	8	High
Lombardo & Islam 2023[26]	N	Y	Y	Y	Y	Y	N	Y	Y	Y	Y	9	High
Everard et al. 2021[27]	N	Y	U	Y	Y	N	N	Y	Y	Y	Y	7	Medium
Hwang et al. 2021[28]	N	Y	Y	Y	Y	N	Y	Y	N	N	Y	7	Medium
N (Yes)	5	27	23	28	24	21	10	24	13	15	25		
%	18%	96%	82.1%	100%	86%	75%	36%	86%	46.4%	53.3%	89.2%		

Answers: Y- Yes, N- No, U- Unclear

Questions:

1. Is the review question clearly and explicitly stated?
2. Were the inclusion criteria appropriate for the review question?
3. Was the search strategy appropriate?
4. Were the sources and resources used to search for studies adequate?
5. Were the criteria for appraising studies appropriate?
6. Was critical appraisal conducted by two or more reviewers independently?
7. Were there methods to minimize errors in data extraction?
8. Were the methods used to combine studies appropriate?
9. Was the likelihood of publication bias assessed?
10. Were recommendations for policy and/or practice supported by the reported data?
11. Were the specific directives for new research appropriate?

References:

1. Laver, K.E.; Adey-Wakeling, Z.; Crotty, M.; Lannin, N.A.; George, S.; Sherrington, C. Telerehabilitation services for stroke. *Cochrane Database of Systematic Reviews* **2020**.
2. Sharififar, S.; Ghasemi, H.; Geis, C.; Azari, H.; Adkins, L.; Speight, B.; Vincent, H.K. Telerehabilitation service impact on physical function and adherence compared to face-to-face rehabilitation in patients with stroke: A systematic review and meta-analysis. *PM & R : the journal of injury, function, and rehabilitation* **2023**, doi:doi:https://dx.doi.org/10.1002/pmrj.12988.
3. Tchero, H.; Teguo, M.T.; Lannuzel, A.; Rusch, E.; Tabue Teguo, M. Telerehabilitation for Stroke Survivors: Systematic Review and Meta-Analysis. *Journal of Medical Internet Research* **2018**, *20*, 80-80, doi:doi:10.2196/10867.
4. Ostrowska, P.M.; Sliwinski, M.; Studnicki, R.; Hansdorfer-Korzon, R. Telerehabilitation of Post-Stroke Patients as a Therapeutic Solution in the Era of the Covid-19 Pandemic. *Healthcare (Basel, Switzerland)* **2021**, *9*, doi:doi:https://dx.doi.org/10.3390/healthcare9060654.
5. Chen, J.; Jin, W.; Zhang, X.X.; Xu, W.; Liu, X.N.; Ren, C.C. Telerehabilitation Approaches for Stroke Patients: Systematic Review and Meta-analysis of Randomized Controlled Trials. *J Stroke Cerebrovasc Dis* **2015**, *24*, 2660-2668, doi:10.1016/j.jstrokecerebrovasdis.2015.09.014.
6. Su, Z.; Guo, Z.; Wang, W.; Liu, Y.; Liu, Y.; Chen, W.; Zheng, M.; Michael, N.; Lu, S.; Wang, W.; et al. The effect of telerehabilitation on balance in stroke patients: is it more effective than the traditional rehabilitation model? A meta-analysis of randomized controlled trials published during the COVID-19 pandemic. *Frontiers in neurology* **2023**, *14*, 1156473, doi:doi:https://dx.doi.org/10.3389/fneur.2023.1156473.
7. Appleby, E.; Gill, S.; Hayes, L.; Walker, T.; Walsh, M.; Kumar, S. Effectiveness of telerehabilitation in the management of adults with stroke: A systematic review. *PLOS ONE* **2019**, *14*, doi:doi:10.1371/journal.pone.0225150.
8. Johansson, T.; Wild, C. Telerehabilitation in stroke care--a systematic review. *Journal of Telemedicine & Telecare* **2011**, *17*, 1-6, doi:doi:10.1258/jtt.2010.100105.
9. Alayat, M.; Almatrafi, N.; Almutairi, A.; El Fiky, A.; Elsodany, A. The Effectiveness of Telerehabilitation on Balance and Functional Mobility in Patients with Stroke: A Systematic Review and Meta-Analysis. *INTERNATIONAL JOURNAL OF TELEREHABILITATION* **2022**, *14*, doi:doi:10.5195/ijt.2022.6532.
10. Deshmukh, S.; Madhavan, S. Can post stroke walking improve via telerehabilitation? A systematic review in adults with stroke. *Frontiers in rehabilitation sciences* **2023**, *4*, 1154686, doi:doi:https://dx.doi.org/10.3389/fresc.2023.1154686.
11. Sarfo, F.S.; Ulasavets, U.; Opare-Sem, O.K.; Ovbiagele, B. Tele-Rehabilitation after Stroke: An Updated Systematic Review of the Literature. *J Stroke Cerebrovasc Dis* **2018**, *27*, 2306-2318, doi:10.1016/j.jstrokecerebrovasdis.2018.05.013.
12. Schroder, J.; van Criekinge, T.; Embrechts, E.; Celis, X.; Van Schuppen, J.; Truijen, S.; Saeys, W. Combining the benefits of tele-rehabilitation and virtual reality-based balance training: a systematic review on feasibility and effectiveness. *Disabil Rehabil Assist Technol* **2019**, *14*, 2-11, doi:10.1080/17483107.2018.1503738.
13. Hao, J.; Pu, Y.; Chen, Z.; Siu, K. Effects of virtual reality-based telerehabilitation for stroke patients: A systematic review and meta-analysis of randomized controlled trials. *JOURNAL OF STROKE & CEREBROVASCULAR DISEASES* **2023**, *32*, doi:doi:10.1016/j.jstrokecerebrovasdis.2022.106960.
14. Lazem, H.; Hall, A.; Gomaa, Y.; Mansoubi, M.; Lamb, S.; Dawes, H. The Extent of Evidence Supporting the Effectiveness of Extended Reality Telerehabilitation on Different Qualitative and Quantitative Outcomes in Stroke Survivors: A Systematic Review. *Int J Environ Res Public Health* **2023**, *20*, doi:10.3390/ijerph20176630.

15. Saragih, I.D.; Tarihoran, D.E.T.A.U.; Batubara, S.O.; Tzeng, H.M.; Lin, C.J. Effects of telehealth interventions on performing activities of daily living and maintaining balance in stroke survivors: A systematic review and meta-analysis of randomised controlled studies. *Journal of Clinical Nursing* **2022**, *31*, 2678-2690.
16. Bok, S.; Song, Y.; Lim, A.; Jin, S.; Kim, N.; Ko, G. High-Tech Home-Based Rehabilitation after Stroke: A Systematic Review and Meta-Analysis. *JOURNAL OF CLINICAL MEDICINE* **2023**, *12*, doi:doi:10.3390/jcm12072668.
17. Tarihoran, D.; Daryanti Saragih, I.; Saragih, I.S.; Tzeng, H.M. Effects of videoconferencing intervention on stroke survivors: A systematic review and meta-analysis of randomised controlled studies. *J Clin Nurs* **2023**, *32*, 5938-5947, doi:10.1111/jocn.16716.
18. Rintala, A.; Paivarinne, V.; Hakala, S.; Paltamaa, J.; Heinonen, A.; Karvanen, J.; Sjogren, T. Effectiveness of Technology-Based Distance Physical Rehabilitation Interventions for Improving Physical Functioning in Stroke: A Systematic Review and Meta-analysis of Randomized Controlled Trials. *Arch Phys Med Rehabil* **2019**, *100*, 1339-1358, doi:10.1016/j.apmr.2018.11.007.
19. Zhou, X.; Du, M.; Zhou, L. Use of mobile applications in post-stroke rehabilitation: a systematic review. *Top Stroke Rehabil* **2018**, 1-11, doi:10.1080/10749357.2018.1482446.
20. Rintala, A.; Kossi, O.; Bonnechere, B.; Evers, L.; Printemps, E.; Feys, P. Mobile health applications for improving physical function, physical activity, and quality of life in stroke survivors: a systematic review. *Disabil Rehabil* **2023**, *45*, 4001-4015, doi:10.1080/09638288.2022.2140844.
21. Szeto, S.G.; Wan, H.; Alavinia, M.; Dukelow, S.; MacNeill, H. Effect of mobile application types on stroke rehabilitation: a systematic review. *J Neuroeng Rehabil* **2023**, *20*, 12, doi:10.1186/s12984-023-01124-9.
22. Qin, P.; Cai, C.; Chen, X.; Wei, X. Effect of home-based interventions on basic activities of daily living for patients who had a stroke: a systematic review with meta-analysis. *BMJ open* **2022**, *12*, e056045.
23. Toh, S.F.M.; Chia, P.F.; Fong, K.N.K. Effectiveness of home-based upper limb rehabilitation in stroke survivors: A systematic review and meta-analysis. *Front Neurol* **2022**, *13*, 964196, doi:10.3389/fneur.2022.964196.
24. Nascimento, L.R.; Rocha, R.J.; Boening, A.; Ferreira, G.P.; Perovano, M.C. Home-based exercises are as effective as equivalent doses of centre-based exercises for improving walking speed and balance after stroke: a systematic review. *Journal of physiotherapy* **2022**, *68*, 174-181.
25. Coupar, F.; Pollock, A.; Legg, L.A.; Sackley, C.; Van Vliet, P. Home-based therapy programmes for upper limb functional recovery following stroke. *Cochrane Database of Systematic Reviews* **2012**.
26. Lombardo, C.; Islam, M. Stroke survivors' acceptance and satisfaction of telerehabilitation delivery of physiotherapy services: a systematic review. *PHYSICAL THERAPY REVIEWS* **2023**, doi:doi:10.1080/10833196.2023.2271301.
27. Everard, G.; Luc, A.; Doumas, I.; Ajana, K.; Stoquart, G.; Edwards, M.; Lejeune, T. Self-Rehabilitation for Post-Stroke Motor Function and Activity-A Systematic Review and Meta-Analysis. *NEUROREHABILITATION AND NEURAL REPAIR* **2021**, *35*, 1043-1058, doi:doi:10.1177/15459683211048773.
28. Hwang, N.; Park, J.; Chang, M. Telehealth Interventions to Support Self-Management in Stroke Survivors: A Systematic Review. *HEALTHCARE* **2021**, *9*, doi:doi:10.3390/healthcare9040472.
