# Supplementary material for: Telerehabilitation and Its Impact Following Stroke: An Umbrella Review of Systematic Reviews
Source: J Clin Med. 2024 Dec 26;14(1):50. doi: 10.3390/jcm14010050 (PMC11721391; doi:10.3390/jcm14010050)
Supplement: Supplementary file 1 [file jcm-14-00050-s001.zip › Table S7 findings of the Umbrella review.pdf]

Table S7/ Motor function

Author/ year	Review type	Studies included (N of participants)	Outcome measures	Approach	Comparison (n of studies)	Results	Effect Size (%95 CI)
**Appleby et al. (2019)**[1]	SR	10 (288)	N/R	Videoconferencing. 3D motion equipment and software.	usual care rehabilitation/conventional rehab/ in-person	No significant difference	n/r
**Bok et al. (2023)**[2]	SR/MA	10 (761)	FMA-UE<br>ARAT<br>NHPT<br>BBT	Virtual reality<br>Robot-assisted devices.<br>games	Other rehabilitation technology	Significant	**VR (3 studies):** Hedges`s g, 0.850.(0.314 - 1.385)<br>I^2= 77.5 %
						Significant	**Robort assisted devices (4 studies):**<br>Hedges`s g, 0.129 (0.025 - 0.232)<br>I^2 = 0%
						Insignificant	**Games (3 studies):** Hedges`s g, -0.162 (-0.534 - 0.210)<br>I^2= 77.4 %
**Chen et al. (2015)**[3]	SR/MA	4 (252)	FMA-UE<br>ARAT<br>NHPT	Telephone.<br>Videoconferencing<br>Desktop videophone.<br>Educational videos<br>Digital video disk.<br>Virtual reality systems.	Conventional rehabilitation<br>(n=2)	No significant difference	SMD: 0.05 (-0.09 to 1.09)<br>I^2= 0 %
					In-person rehabilitation (n=2)	No significant difference	n/r

**Coupar et al. (2012)**[4]	SR/MA	2 (46)	FMA- UE<br><br>ABILHAND Scale	Virtual reality<br><br>Video conferencing system	Usual care (n=1)	No significant difference	Following intervention:<br><br>MD: 4.10 (-0.09 to 8.29)<br><br>I^2= n/r
						Significant difference	At follow-up:<br><br>MD: 4.30 (0.19 to 8.41)<br><br>I^2= n/r
					Same therapy in the hospital (n=1)	No significant difference	MD:0.60 (-8.94 to 10.14)<br><br>I^2= n/r
**Deshmukh and Madhavan (2023)**[5]	SR	1 (61)	FMA	Videoconferencing platforms e.g. Skype<br><br>Virtual reality	Rehabilitation guidance via telephone	No significant difference	n/r
**Everard et al. (2021)**[6]	SR/MA	10 (470)	FMA	Technology assisted self-rehabilitation:<br><br>Tablets<br><br>Computers<br><br>Non-immersive VR<br><br>Video instructions.	Conventional rehabilitation (n10)	No significant difference	**UL:** SMD:0.31 (-1.34 to 1.95)<br><br>I^2= 40.44%
						No significant difference	**LL:** SMD: -0.15 (-0.66 to 0.36)<br><br>I^2= 0%

**Hao et al. (2023)**[7]	SR/MA	3 (118)	FMA-UE	Virtual reality	In-person / usual care	Significant effect	SMD:1.05 (0.04 to 2.06)<br>I^2= 84 %
**Johansson and Wild (2011)**[8]	SR	2 (52)	FMA-UE<br>ABILHAND scale.	Telephone.<br>Virtual reality.<br>Video consulting system	usual care rehabilitation	Significant improvements in FMA-UE	n/r
						No significant difference	
**Lazem et al. (2023)**[9]	SR	5 (530)	FMA-UE<br>FMA-LE<br>ARAT<br>NHPT<br>BBT<br>MAL	Extended reality: non-immersive VR; semi-immersive VR and augmented reality (AR).	In clinic rehabilitation (n=3)	Significant improvements in FMA-UE.	n/r
						No significant difference FMA-LE	
					Home rehabilitation<br>(n=2)	Significant improvements	
						No improvement in hand function	
**Laver et al. (2020)**[10]	SR/MA	6 (240)	<u>UL function:</u><br>ARAT<br>NHPT	Telephone.<br>Videoconferencing.<br>Desktop videophone.<br>Combination of telephone calls, an in-home messaging device, and video. recordings.	In-person (n= 3)	No significant difference	MD 1.23(-2.17 to 4.64)<br>I^2= 42.41%
					Usual care (n=3)	No significant difference	SMD: 0.33 (-0.21 to 0.87)<br>I^2= 0 % (2 studies)
						No significant difference	n/r
**Nascimento et al. (2022)**[11]	SR/MA	3 (170)	FMA- UE<br>MAL	Virtual reality	Clinic-based exercise (n=3)	No significant difference	MD:1.29 (-2.37 to 4.96)<br>I^2= 48.27%

**Ostrowaska et al. (2021)**[12]	SR	1 (124)	FMA- UE	Gaming input devices<br>Internet-enabled computer.	In-person rehabilitation	Significant effect	n/r
**Rintala et al. (2019)**[13]	SR/MA	6 (261)	**UE:**<br>FMA<br>WMFT<br>LLFDI<br>**LE:**<br>LLFDI<br>FMA	Telephone calls.<br>Messaging.<br>Exercise Video through electronic tablet.<br>Virtual training programs.	usual care rehabilitation/or the same training in intervention but without technology	No significant difference	n/r
**Rintala et al. (2023)**[14]	SR	3 (111)	**UE motor function:**<br>FMA-UE<br>MMT<br>B-stage for arm and hand<br>MFT<br>PPT<br>**LE motor function:**<br>FMA-LE<br>MI-LE	Smart phone- based mHealth	usual care/ no training	**UE:** Significant improvement<br>**LE**: No significant difference	n/r
**Sarfo et al. (2018)**[15]	SR	7 (356)	ARAT<br>WMFT<br>FMA<br>(SIS-mobility domains)<br>Motor Subscale of Telephone Version of FIM.	Phone-based<br>Computer based<br>Tablet based<br>Video based	usual care rehabilitation (n=4)	No significant difference	n/r
					conventional rehabilitation/ Home Exercise Program (HEP) (n=3)	No significant difference	n/r

**Su et al. (2023)**[16]	MA	3 (143)	FMA	Telephone.<br>Videoconferencing.	Conventional rehabilitation	Significant difference/ effective	**FMA:**<br>MD: 8.12 (6.35 to 9.88)<br>I^2= 0 % (3 studies)
**Szeto et al. (2023)**[17]	SR	4 (158)	WMFT<br>MFAC<br>MFT<br>PPT	Mobile apps	Exercise in a written format (paper-based)/or conventional rehabilitation	Significant improvement	n/r
**Tchero et al. (2018)**[18]	SR/MA	6 (N/R)	FMA-UE<br>ARAT<br>(SIS-mobility subscale)	Telephone calls.<br>Videoconferencing.<br>Educational Videos.<br>Web-based chat.<br>Virtual reality systems.	Usual care (n= 6)	No significant difference<br>No significant difference<br>No significant difference	**FMA-UE**<br>SMD: 0,50 (-0.09 to 1.09)<br>I^2= 0 %<br>**ARAT**<br>SMD: -0,06 (-0.46 to 0.33)<br>I^2= 0 %<br>**(SIS-mobility subscale)**<br>SMD: 0.018 (-0.13 to 0.48)<br>I^2= 0 %
**Toh et al. (2022)**[19]	SR/MA	6 (465)	FMA<br>ARAT<br>WMFT<br>NHPT<br>BBT<br>MAL	Virtual reality	Clinic based therapy (n= 1)<br>No technology intervention (home-based-ex handout or/ home-based task-specific training) (n =4)	No significant difference<br>No significant difference	SMD: 0.04 (-0.32 to 0.39)<br>I^2= 0 %<br>After treatment: SMD: 0.08 (-0.22 to 0.37)<br>I^2= 25 %<br>At follow-up:

					No intervention (n=1)	No significant difference	SMD:-0.10 (-0.36 to 0.15)<br>I^2= 0 %<br>SMD: 0.33 (-0.60 to 1.26)<br>I^2= n/r
**Zhou et al. (2018)**[20]	SR	2 (45)	FMA<br>NHPT<br>BBT	Smart phone-based application<br>Tablet based application	usual care/ no training	Significant effect	n/r

Outcomes measures: Fugl-Meyer Assessment (FMA-UE), (FMA-LE), Action Research Arm Test (ARAT), Nine-Hole Peg Test (NHPT), Box and Block Test (BBT), ABILHAND Scale, Motor Activity Log (MAL), Wolf Motor Function Test (WMFT), Late-Life Function and Disability Instrument (LLFDI), Brunnstrom- stage for arm and hand(B-stage), Manual Function Test (MFT), Prude Pegboard Test (PPT), Manual Muscle testing for the UE, Motricity Index of the Lower Extremity (MI-LE), Stroke Impact Scale-Mobility domain (SIS-Mobility), Motor Subscale of Telephone Version of Functional Independence Measure (FIM) and modified Functional Ambulatory Category (MFAC). The color coding in the table pertains to the following: The green indicates the intervention is effective (beneficial), the orange indicates the intervention has no difference compared to the control group, and the red indicates the intervention has no effect.

Table S7/ balance

Author/ year	Review type	Studies included	Outcome measures	Approach	Comparison (n of studies)	Results	Effect Size(%95 CI)
**Alayat et al. (2022)**[21]	SR/MA	13 (530)	BBS POMA-B BBA Mini-BEST Step Test.	Telephone. Videoconferencing. Platform monitoring. Text messaging.	Conventional rehabilitation(n=13)	Small significant effect	SMD= 0.33 (0.03 to 0.63) I^2= 63%(n=13)
						Significant improvement on BBS	n/r (n=2)
						No significant difference in BBS	n/r (n=11)
						No significant difference in (POMA-B), (BBA),(Mini-BEST), and Step Test.	n/r (n=3)
**Chen et al. (2015)**[3]	SR/MA	2 (53)	BBS	Telephone. VideoconferencingDesktop videophone. Educational videos Digital video disk. Virtual reality systems.	conventional rehabilitation (n = 2)	No effect	SMD: -0.07 (-0.7 to 0.37) I^2= 0 %
**Deshmukh and Madhavan (2023)**[5]	SR	5 (199)	BBS TUG	Videoconferencing platforms e.g. Skype Virtual reality	in-person rehabilitation/ conventional rehabilitation/or rehabilitation guidance via telephone (n=5)	No significant difference	n/r
**Hao et al. (2023)**[7]	SR/MA	5 (132)	BBS TUG	Virtual reality	In-person (n=5)	No significant difference (BBS)	SMD= 0.05 (-0.30 to 0.40) I^2= 0% (n= 5 studies)
						No significant difference (TUG)	n/r (n=2 studies)
**Laver et al. (2020)**[10]	SR/MA	3 (106)	N/A	Telephone. Videoconferencing. Desktop videophone. Combination of telephone calls, an in-home messaging device, and video. recordings.	In-person (n = 3)	No significant difference	MD: 0.48 (-1.36 to 2.32) I^2= 0 %
**Lazem et al. (2023)**[9]	SR	3 (170)	BBS POMA-B TUG	Extended reality: non-immersive VR; semi-immersive VR and augmented reality (AR).	in-clinic rehabilitation (n=2)	No significant difference (n=1)	n/r
						No effect (n=1)	

					Home rehabilitation (n=1)	Significant improvement	n/r
**Ostrowaska et al. (2021)**[12]	SR	1 (6)	BBS	Games installed on smartphones.<br>Inertial motion sensors (IMUs) and cloud databases.	In-person (n = 1)	Significant improvement	n/r
**Rintala et al. (2019)**[13]	SR/MA	4 (198)	BBS	Telephone calls.<br>Messaging.<br>Exercise Video through the electronic tablet.<br>Virtual training programs.	usual care (n=4)	No significant difference	n/r
**Rintala et al. (2023)**[14]	SR	3 (106)	BBS<br>MBT	Smart phone- based mHealth	usual care (n=3)	Significant improvement (n=1)<br>No significant difference (n=2)	n/r
**Saragih et al. (2022)**[22]	SR/MA	3 (90)	BBS	Telephone calls.<br>mHealth apps.<br>Videoconferencing.<br>3D animation Ex's videos.	usual care rehabilitation/ conventional rehabilitation (n=3)	No significant difference	SMD: 0,03 (-0.38 to 0.45)<br>I^2= 0 %
**Schroder et al. (2018)**[23]	SR	4 (106)	BBS.<br>POMA-B.	Virtual reality system<br>Videoconferencing	in-person rehabilitation	No significant difference	n/r
**Su et al. (2023)**[16]	MA	8 (256)	BBS.<br>POMA-B.<br>S-TIS.<br>TUG	Telephone.<br>Videoconferencing.	conventional rehabilitation	Significant improvement<br><br>Significant improvement<br><br>Significant difference (control group better than intervention group)<br><br>No significant difference	**BBS:** MD= 2.80 (0.61 to 4.98)<br>I^2= 51.90% (8 studies)<br><br>**POMA-Balance:** MD= 2.50 (0.39 to 4.61)<br>I^2= n/r (1 study)<br><br>**TUG:**<br>MD: - 4.59 (-5.93 to -3.25)<br>I^2= 0 % (5 studies)<br><br>**(S-TIS):** MD= 1.37 (1.85 to 4.60)<br>I^2= 65.61% (2 study)
**Tarihoran et al. (2023)**[24]	SR/MA	4 (160)	BBS	Videoconferencing.	in-person rehabilitation/usual care/ waiting list control(n=4)	Significant improvement	SMD=1.96 (1.27 to 2.66)<br>I^2= 66.49%
**Tchero et al. (2018)**[18]	SR/MA	4 (N/R)	BBS	Telephone calls.<br>Videoconferencing.<br>Educational Videos.	usual care rehabilitation(n=4)	No significant difference	SMD: -0.04 (-0.34 to 0.26)<br>I^2= 0 %

	Web-based chat.<br>Virtual reality systems.		

Outcomes measures: the Berg Balance Scale (BBS), the Timed Up and Go test (TUG), Tinetti performance-oriented mobility assessment- balance (POMA-B), the Spanish Version of the trunk impairment scale (S-TIS), Brunel Balance Assessment (BBA), Mini Balance Evaluation System Test (Mini-Best) and step test. The color coding in the table pertains to the following: The green indicates the intervention is effective (beneficial), the orange indicates the intervention has no difference compared to the control group, and the red indicates the intervention has no effect.

Table S7/ Gait

Author/ year	Review type	Studies included	Outcome measures	Approach	Comparison (n of studies)	Results	Effect Size (%95 CI)
**Deshmukh and Madhavan (2023)[5]**	SR	3 (107)	10-MWT<br>6-MWT<br>POMA-G.	Videoconferencing platforms e.g. Skype<br>Virtual reality	Rehabilitation guidance via telephone or no control (means both groups received different complexities of the same movement protocol) or in-person rehabilitation	No significant difference, however, both groups showed significant improvement (n=2)<br>No improvements (n=1)	n/r
**Hao et al. (2023)[7]**	SR/MA	3 (78)	2-MWT<br>10-MWT<br>FAC	Virtual reality	usual care (passive control)/ or in-person rehabilitation (active control).	No significant difference (n=3)<br>Significant improvement after 3 months follow-up (n=1)	n/r
**Lazem et al. (2023)[9]**	SR	2 (114)	POMA-G<br>FAC	Extended reality: non- immersive VR; semi-immersive VR and augmented reality (AR).	in-clinic rehabilitation	Significant improvement (n=1)<br>No effect (n=1)	n/r
**Rintala et al. (2019)[13]**	SR/MA	3 (165)	10-MWT	Telephone calls.<br>Messaging.<br>Exercise Videos through electronic tablet.<br>Virtual training programs.	Home-based exercises/ or usual care	Significant improvement	n/r
**Rintala et al. (2023)[14]**	SR	3 (130)	10-MWT<br>6MWT	Smart phone- based mHealth	usual care/ or no rehabilitation	Significant improvement(n=1)<br>No significant difference (n=2)	n/r
**Schroder et al. (2018)[23]**	SR	3 (73)	POMA-G<br>TUG<br>10-MWT<br>SUE<br>SAE	Virtual reality system<br>Videoconferencing	Conventional rehabilitation in the clinic	No significant difference	n/r

**Zhou et al. (2018)**[20]	SR	1 (24)	10-MWT	Smart phone-based application<br><br>Tablet based application	Usual care	Significant improvement	n/r

Outcomes measures: 10 -Meter Walk Test (10-MWT), Six Minute Walk Test (6- MWT), Tinetti Performance Oriented Mobility Assessment- gait subscale (POMA-G), Standing Unaffected Leg (SUE), Standing affected Leg (SAE), Two-Minute Walk Test (2-MWT), Timed Up and Go test (TUG) and Functional Ambulatory Category (FAC). No meta-analyses have evaluated the effectiveness of interventions on walking ability. The color coding in the table pertains to the following: The green indicates the intervention is effective (beneficial), the orange indicates the intervention has no difference compared to the control group, and the red indicates the intervention has no effect.

Table S7/ ADL

Author/ year	Review type	Studies included	Outcome measures	Approach	Comparison (n of studies)	Results	Effect Size(%95 CI)
**Appleby et al. (2019)**[1]	SR	3 (104)	N/A	Videoconferencing. 3D motions equipment's and software.	In-person rehabilitation	No significant difference	n/r
**Bok et al. (2023)**[2]	SR/MA	1 (37)	Nottingham Extended ADL scale	Virtual reality	other rehabilitation technology (n=1)	significant effect	**VR**: Hedges`s g, 0.850. (0.314 - 1.385) I^2= 77.5 %
**Chen et al. 2015**[3]	SR/MA	6 (853)	BI FONEFIM	Telephone. Videoconferencing. Desktop videophone. Educational videos Digital video disk. Virtual reality systems.	conventional rehabilitation(n=5)	No significant difference	SMD: -0.05 (-0.24 to 0.13) I^2= 73 % (4 studies)
						No significant difference	n/r
					In-person (n=1)	No significant difference, although both groups showed significant improvement	n/r
**Deshmukh and Madhavan (2023)**[5]	SR	3 (139)	MBI BI MRS	Videoconferencing platforms e.g. Skype Virtual reality	In-person (n=1)	Significant effect	n/r
					conventional rehabilitation(n=2)	No significant difference, both groups reported significant improvement in the scores.	n/r
**Hao et al. (2023)**[7]	SR/MA	1 (24)	BI	Virtual reality	in-person rehabilitation(n=1)	No significant difference, both groups reported significant improvements.	n/r
**Hwang et al. (2021)**[25]	SR	3 (122)	FONEFIM	Telephone & Messaging (Sync+ Async).	Usual care/ waiting list(n=3)	significant effect	n/r

				Web-based (Video conferencing) (Sync).			
**Laver et Al. (2020)**[10]	SR/MA	7 (840)	Frenchay Activities Index	Telephone. Videoconferencing. Desktop videophone. Combination of telephone calls, an in-home messaging device, and video. recordings.	In-person (n = 3)<br>Usual Care (n=4)	No significant difference<br>No significant difference<br>No significant difference	MD: 0.59 (-5.50 to 6.68) I^2= 0 % (2 studies)<br>SMD: -0.00 (-0.15 to 0.15) I^2 = 0 % (2 studies)<br>n/r
**Nascimento et al. (2022)**[11]	SR/MA	1 (124)	BI	Virtual reality	Clinic-based exercise(n=1)	No significant difference	SMD: -0.12 (-0.47 to 0.22) I^2=N/R for one study.
**Rintala et al. (2019)**[13]	SR/MA	6 (328)	MBI<br>BI<br>MRS<br>FONEFIM	Telephone calls. Messaging. Exercise Video through electronic tablet. Virtual training programs.	usual care rehabilitation/ or same intervention without use technology (paper-based)	No significant difference	SMD: 0.06 (-0.22 to 0.35) I^2= 38%
**Rintala et al. (2023)**[14]	SR	4 (141)	MBI<br>BI<br>(SIS-mobility)	Smart phone-based mHealth	usual care(n=4)	Significant improvement<br>No significant difference	n/r<br>n/r
**Saragih et al. (2022)**[22]	SR/MA	5 (190)	MBI<br>BI	Telephone calls. mHealth apps. Videoconferencing. 3D animation Ex's videos.	usual care rehabilitation/ conventional rehabilitation	Significant difference/ effective	SMD:0.45 (0.12 to 0.78) I^2= 21.51 %
**Sharififar et al. (2023)**[26]	SR/MA	4 (363)	BI	Phone calls. DVD records video conferencing	in-person rehabilitation (n=4)	Significant improvement	MD: 4.18 (1.79 to 6.57) I^2= 16 %
**Su et al. (2023)**[16]	MA	3 (86)	S-FIST<br>TIS	Telephone Videoconferencing	conventional rehabilitation(n-3)	No significant difference	(S-FIST) MD: 2.24 (-0.96 to 5.44) I^2= 0 %

						No significant difference	(TIS) MD: -2.14 (-6.91 to 2.63) I^2= 0 %
**Szeto et al. (2023)**[17]	SR	4 (293)	BI<br>MBI	Mobile apps	conventional rehabilitation/ or Exercise in a written format (paper-based)	Significant effect(n=1)	n/r
						No effect (n=3)	n/r
**Tarihoran et al. (2023)**[24]	SR/MA	4 (318)	MBI	Videoconferencing	in-person rehabilitation/ usual care/ telephone follow-up (n=4)	Significant effect	SMD: 0.57 (0.13 to 1.01) I^2 =71.99%
**Tchero et al. (2018)**[18]	SR/MA	6 (N/R)	BI	Telephone calls.<br>Videoconferencing.<br>Educational Videos.<br>Web-based chat.<br>Virtual reality systems.	usual care rehabilitation(n=6)	No significant difference	SMD: -0.05 (-0.18 to 0.08) I^2 = 0 %
**Qin et al. (2022)**[27]	SR/MA	5 (340)	MBI<br>FONEFIM<br>mRS	Phone calls.<br>video conferencing	Institution based rehabilitation(n=4)	No significant difference	SMD: 0.12 (-0.41 to 0.66) I^2= 0 % (1 study
						No significant difference	n/r
					usual care rehabilitation(n=1)	Significant effect	SMD:0.29 (-0.31 to 0.88) I^2= 3% (1 study)
**Zhou et al. (2018)**[20]	SR	1 (24)	Instrumental Activities of Daily Living Scale.	Smart phone-based application<br>Tablet based application	usual care (n=1)	Significant effect	n/r

Outcomes measures: Barthel Index (BI), modified Barthel Index (mBI), modified Rankin Scale (mRS), Telephone version of Functional Independence Measure (FONEFIM), the Spanish version of the function in sitting test (S-FIST), Trunk Impairment Scale (TIS), Nottingham Extended ADL Scale, Frenchay Activities Index, Stroke Impact Scale- mobility (SIS-mobility) and Instrumental activities of daily living scale. The color coding in the table pertains to the following: The green indicates the intervention is effective (beneficial), the orange indicates the intervention has no difference compared to the control group, and the red indicates the intervention has no effect.

Table S7/ QoL

Author/ year	Review type	Studies included	Outcome measures	Approach	Comparison (n of studies)	Results	Effect Size (%95 CI)
**Appleby et al. (2019)**[1]	SR	3 (122)	SF-12	Videoconferencing. 3D motions equipment's and software.	in-person rehabilitation/ or usual care rehabilitation	No significant difference<br>Significant improvement	n/r
**Chen et al. (2015)**[3]	SR/MA	3 (735)	QoL: Short Form Health Survey	Telephone. Videoconferencing. Desktop videophone. Educational videos Digital video disk. Virtual reality systems.	Conventional rehabilitation	No significant difference	n/r
**Deshmukh and Madhavan (2023)**[5]	SR	1 (61)	Stroke-Specific Quality of Life Questionnaire (SS-QOL)	Videoconferencing platforms e.g. Skype Virtual reality	Rehabilitation guidance via telephone	Significant improvement	n/r
**Hao et al. (2023)**[7]	SR/MA	1 (58)	European Quality of Life-5 Dimension-5 Level (EQ-5D)	Virtual reality	usual care	No significant difference	n/r
**Laver et al. (2020)**[10]	SR/MA	5 (1116)	SF-36	Telephone. Videoconferencing. Desktop videophone. Combination of telephone calls, an in-home messaging device, and video. recordings.	usual care rehabilitation (n=4)<br><br>In-person (n=1)	No significant difference<br>Significant improvement<br>No significant difference	SMD: 0.03 (-0.14 to 0.20) I^2= 5. 42 % (3 studies<br>n/r<br>n/r
**Rintala et al. (2023)**[14]	SR	4 (154)	EQ-5D SS-QOL	Smart phone-based mHealth	usual care/ or No rehabilitation (n=4)	Significant improvement<br>No significant difference	n/r
**Szeto et al. (2023)**[17]	SR	2 (66)	N/R	Mobile apps (therapy apps)	conventional rehabilitation	Significant improvement<br>No effect	n/r
**Tchero et al. (2018)**[18]	SR/MA	5 (N/R)	SF-12, SF-36 Or/ (2 different versions of the	Telephone calls. Videoconferencing. Educational Videos.	usual care rehabilitation (n=5)	Significant improvement	MD: 7.9 (0.1 to 15.7) I^2= n/r (1 study)

	Functional Independence Measure: self-administrated and telephone versions)	Web-based chat. Virtual reality systems.	No significant difference	n/r

Outcomes measures: Short Form Health Survey, Stroke-Specific Quality of Life Questionnaire (SS-QoL), European Quality of Life-5 Dimension-5 Level (EQ-5D), 36-Item Short Form Survey (SF-36), 12-Item Short Form Survey (SF-12), and 2 different versions of the Functional Independence Measure: self-administrated and telephone versions). The color coding in the table pertains to the following: The green indicates the intervention is effective (beneficial), the orange indicates the intervention has no difference compared to the control group, and the red indicates the intervention has no effect.

Table S7/ adherence to treatment

Author/ year	Review type	Studies included	Outcome measures	Approach	Comparison (n of studies)	Results	Effect Size (%95 CI)
**Deshmukh and Madhavan (2023)**[5]	SR	8 (248)	Rate of participant dropout	Videoconferencing platforms e.g. Skype Virtual reality	in-person rehabilitation/ conventional rehab/ rehabilitation guidance via telephone	a good adherence to telerehabilitation.	n/r
**Rintala et al. (2023)**[14]	SR	2 (71)	N/R	Smart phone-based mHealth	usual care	Adherence of 50% of daily walking and 14% including core-stability exercises.	n/r
**Schroder et al. (2018)**[23]	SR	1 (30)	N/R	Virtual reality system	in-person rehabilitation	Excellent adherence to home-based therapy	n/r
**Szeto et al. (2023)**[17]	SR	3 (137)	N/R	Mobile apps (rehab videos/ therapy apps/ reminder)	conventional rehabilitation/ Exercise in a written format	increase in exercise adherence.	n/r

Table S7/ Participants satisfaction with Intervention

Author/ year	Review type	Studies included	Outcome measures	Approach	Comparison (n of studies)	Results	Effect Size (%95 CI)
**Appleby et al. (2019)**[1]	SR	5 (171)	N/R	Videoconferencing 3D motions equipment and software.	Usual care/ in-person rehabilitation	Patients were generally satisfied with telerehabilitation	n/r
**Chen et al. (2015)**[3]	SR/MA	4 (585)	SASC.<br>12-item Satisfaction Questionnaire.<br>Satisfaction Questionnaire.	Telephone.<br>Videoconferencing<br>Desktop videophone.<br>Educational videos<br>Digital video disk.<br>Virtual reality systems.	Conventional rehabilitation	No significant difference	n/r
**Hao et al. (2023)**[7]	SR/MA	2 (34)	12-item Satisfaction Questionnaire.<br>Satisfaction Questionnaire.	Virtual reality	Conventional rehabilitation	No significant difference	n/r
					in-person rehabilitation	Tele VR showed equal or higher scores	
**Johansson and Wild (2011)**[8]	SR	2 (85)	SASC.<br>Satisfaction Questionnaire.	Telephone.<br>Video consulting system	Usual care rehabilitation (n=2)	Both participants and medical professionals expressed satisfaction about the usage of telerehabilitation services.	n/r
**Laver et al. (2020)**[10]	SR/MA	5 (662)	N/R	Telephone.<br>Videoconferencing<br>Desktop videophone.<br>Combination of telephone calls, an in-home messaging device, and video recordings.	In-person rehabilitation (n=2)	No significant difference	n/r
					Usual care rehabilitation (n=3)	No significant difference	
**Lombardo & Islam (2023)**[28]	SR	1 (52)	(SSPSC) scale	Voice calls and In-Home Messaging Device (IHMD).	Usual care rehabilitation (n=1)	No significant difference	n/r

**Sharififar et al. (2023)**[26]	SR/MA	1 (124)	Satisfaction survey scores	Videoconferencing game-based therapy	In-person rehabilitation (n=1)	Patients have high rate of satisfaction (78%-90%)	n/r
**Tchero et al. (2018)**[18]	SR/MA	3 (N/R)	Modified satisfaction questionnaire	Telephone calls. Videoconferencing Educational videos. Web-based chat. Virtual reality systems.	Usual care rehabilitation (n=2)<br>In-person rehabilitation (n=1)	No significant difference<br>Tele VR showed equal or higher scores	n/r

Outcomes measures: satisfaction with stroke care questionnaire (SASC), 12-item satisfaction questionnaire, satisfaction questionnaire, Strok-Specific Patient Satisfaction with Care (SSPSC) scale, Satisfaction survey scores, and Modified satisfaction questionnaire.

Table S7/ cost.

Author/ year	Review type	Studies included	Outcome measures	Approach	Comparison (n of studies)	Results	Effect Size (%95 CI)
**Chen et al. (2015)**[3]	SR/MA	1 (30)	cost	Virtual reality	In-person rehabilitation (n=1)	The expenses for 1 participant in the telerehabilitation group were lower than those in conventional rehabilitation in the clinic group by approximately US $ 654.72	n/r
**Deshmukh and Madhavan (2023)**[5]	SR	1 (30)	cost	Virtual reality	In-person rehabilitation (n=1)	Telerehabilitation is less expensive than in-person therapy.	n/r
**Hao et al. (2023)**[7]	SR/MA	1 (30)	Cost	Virtual reality	Virtual reality hospital-based (n=1)	The expenses of VR in the clinic (average per person $ 654.72) are higher than VR-based telerehabilitation	n/r
**Sarfo et al. (2018)**[15]	SR	1 (30)	Cost	Virtual reality	in-person rehabilitation (n=1)	In-clinic intervention is more expensive than a Telerehabilitation program.	n/r
**Schroder et al. (2018)**[23]	SR	2 (47)	cost	Virtual reality	in-person rehabilitation (n=2)	The cost of telerehabilitation was lower than those in clinic rehabilitation.<br>Telerehabilitation requires specific equipment which can be cost-demanding.	n/r
**Tchero et al. (2018)**[18]	SR/MA	1 (N/R)	Cost	Virtual reality	Usual care (n=1)		n/r

			The cost of telerehabilitation was lower than usual care by approximately US $ 654.	

1. Appleby, E.; Gill, S.; Hayes, L.; Walker, T.; Walsh, M.; Kumar, S. Effectiveness of telerehabilitation in the management of adults with stroke: A systematic review. *PLOS ONE* **2019**, *14*, doi:doi:10.1371/journal.pone.0225150.
2. Bok, S.; Song, Y.; Lim, A.; Jin, S.; Kim, N.; Ko, G. High-Tech Home-Based Rehabilitation after Stroke: A Systematic Review and Meta-Analysis. *JOURNAL OF CLINICAL MEDICINE* **2023**, *12*, doi:doi:10.3390/jcm12072668.
3. Chen, J.; Jin, W.; Zhang, X.X.; Xu, W.; Liu, X.N.; Ren, C.C. Telerehabilitation Approaches for Stroke Patients: Systematic Review and Meta-analysis of Randomized Controlled Trials. *J Stroke Cerebrovasc Dis* **2015**, *24*, 2660-2668, doi:10.1016/j.jstrokecerebrovasdis.2015.09.014.
4. Coupar, F.; Pollock, A.; Legg, L.A.; Sackley, C.; Van Vliet, P. Home-based therapy programmes for upper limb functional recovery following stroke. *Cochrane Database of Systematic Reviews* **2012**.
5. Deshmukh, S.; Madhavan, S. Can post stroke walking improve via telerehabilitation? A systematic review in adults with stroke. *Frontiers in rehabilitation sciences* **2023**, *4*, 1154686, doi:doi:https://dx.doi.org/10.3389/fresc.2023.1154686.
6. Everard, G.; Luc, A.; Doumas, I.; Ajana, K.; Stoquart, G.; Edwards, M.; Lejeune, T. Self-Rehabilitation for Post-Stroke Motor Function and Activity-A Systematic Review and Meta-Analysis. *NEUROREHABILITATION AND NEURAL REPAIR* **2021**, *35*, 1043-1058, doi:doi:10.1177/15459683211048773.
7. Hao, J.; Pu, Y.; Chen, Z.; Siu, K.-C. Effects of virtual reality-based telerehabilitation for stroke patients: A systematic review and meta-analysis of randomized controlled trials. *Journal of Stroke and Cerebrovascular Diseases* **2023**, *32*, 106960.
8. Johansson, T.; Wild, C. Telerehabilitation in stroke care--a systematic review. *Journal of Telemedicine & Telecare* **2011**, *17*, 1-6, doi:doi:10.1258/jtt.2010.100105.
9. Lazem, H.; Hall, A.; Gomaa, Y.; Mansoubi, M.; Lamb, S.; Dawes, H. The Extent of Evidence Supporting the Effectiveness of Extended Reality Telerehabilitation on Different Qualitative and Quantitative Outcomes in Stroke Survivors: A Systematic Review. *Int J Environ Res Public Health* **2023**, *20*, doi:10.3390/ijerph20176630.
10. Laver, K.E.; Adey-Wakeling, Z.; Crotty, M.; Lannin, N.A.; George, S.; Sherrington, C. Telerehabilitation services for stroke. *Cochrane Database of Systematic Reviews* **2020**.
11. Nascimento, L.R.; Rocha, R.J.; Boening, A.; Ferreira, G.P.; Perovano, M.C. Home-based exercises are as effective as equivalent doses of centre-based exercises for improving walking speed and balance after stroke: a systematic review. *Journal of physiotherapy* **2022**, *68*, 174-181.
12. Ostrowska, P.M.; Sliwinski, M.; Studnicki, R.; Hansdorfer-Korzon, R. Telerehabilitation of Post-Stroke Patients as a Therapeutic Solution in the Era of the Covid-19 Pandemic. *Healthcare (Basel, Switzerland)* **2021**, *9*, doi:doi:https://dx.doi.org/10.3390/healthcare9060654.
13. Rintala, A.; Paivarinne, V.; Hakala, S.; Paltamaa, J.; Heinonen, A.; Karvanen, J.; Sjogren, T. Effectiveness of Technology-Based Distance Physical Rehabilitation Interventions for Improving Physical Functioning in Stroke: A Systematic Review and Meta-analysis of Randomized Controlled Trials. *Arch Phys Med Rehabil* **2019**, *100*, 1339-1358, doi:10.1016/j.apmr.2018.11.007.
14. Rintala, A.; Kossi, O.; Bonnechere, B.; Evers, L.; Printemps, E.; Feys, P. Mobile health applications for improving physical function, physical activity, and quality of life in stroke survivors: a systematic review. *Disabil Rehabil* **2023**, *45*, 4001-4015, doi:10.1080/09638288.2022.2140844.
15. Sarfo, F.S.; Ulasavets, U.; Opare-Sem, O.K.; Ovbiagele, B. Tele-Rehabilitation after Stroke: An Updated Systematic Review of the Literature. *J Stroke Cerebrovasc Dis* **2018**, *27*, 2306-2318, doi:10.1016/j.jstrokecerebrovasdis.2018.05.013.
16. Su, Z.; Guo, Z.; Wang, W.; Liu, Y.; Liu, Y.; Chen, W.; Zheng, M.; Michael, N.; Lu, S.; Wang, W.; et al. The effect of telerehabilitation on balance in stroke patients: is it more effective than the traditional rehabilitation model? A meta-analysis of randomized controlled trials published during the COVID-19 pandemic. *Frontiers in neurology* **2023**, *14*, 1156473, doi:doi:https://dx.doi.org/10.3389/fneur.2023.1156473.
17. Szeto, S.G.; Wan, H.; Alavinia, M.; Dukelow, S.; MacNeill, H. Effect of mobile application types on stroke rehabilitation: a systematic review. *J Neuroeng Rehabil* **2023**, *20*, 12, doi:10.1186/s12984-023-01124-9.
18. Tchero, H.; Teguo, M.T.; Lannuzel, A.; Rusch, E.; Tabue Teguo, M. Telerehabilitation for Stroke Survivors: Systematic Review and Meta-Analysis. *Journal of Medical Internet Research* **2018**, *20*, 80-80, doi:doi:10.2196/10867.
19. Toh, S.F.M.; Chia, P.F.; Fong, K.N.K. Effectiveness of home-based upper limb rehabilitation in stroke survivors: A systematic review and meta-analysis. *Front Neurol* **2022**, *13*, 964196, doi:10.3389/fneur.2022.964196.
20. Zhou, X.; Du, M.; Zhou, L. Use of mobile applications in post-stroke rehabilitation: a systematic review. *Top Stroke Rehabil* **2018**, 1-11, doi:10.1080/10749357.2018.1482446.
21. Alayat, M.; Almatrafi, N.; Almutairi, A.; El Fiky, A.; Elsodany, A. The Effectiveness of Telerehabilitation on Balance and Functional Mobility in Patients with Stroke: A Systematic Review and Meta-Analysis. *INTERNATIONAL JOURNAL OF TELEREHABILITATION* **2022**, *14*, doi:doi:10.5195/ijt.2022.6532.
22. Saragih, I.D.; Tarihoran, D.E.T.A.U.; Batubara, S.O.; Tzeng, H.M.; Lin, C.J. Effects of telehealth interventions on performing activities of daily living and maintaining balance in stroke survivors: A systematic review and meta-analysis of randomised controlled studies. *Journal of Clinical Nursing* **2022**, *31*, 2678-2690.
23. Schroder, J.; van Criekinge, T.; Embrechts, E.; Celis, X.; Van Schuppen, J.; Truijen, S.; Saeys, W. Combining the benefits of tele-rehabilitation and virtual reality-based balance training: a systematic review on feasibility and effectiveness. *Disabil Rehabil Assist Technol* **2019**, *14*, 2-11, doi:10.1080/17483107.2018.1503738.
24. Tarihoran, D.; Daryanti Saragih, I.; Saragih, I.S.; Tzeng, H.M. Effects of videoconferencing intervention on stroke survivors: A systematic review and meta-analysis of randomised controlled studies. *J Clin Nurs* **2023**, *32*, 5938-5947, doi:10.1111/jocn.16716.
25. Hwang, N.; Park, J.; Chang, M. Telehealth Interventions to Support Self-Management in Stroke Survivors: A Systematic Review. *HEALTHCARE* **2021**, *9*, doi:doi:10.3390/healthcare9040472.
26. Sharififar, S.; Ghasemi, H.; Geis, C.; Azari, H.; Adkins, L.; Speight, B.; Vincent, H.K. Telerehabilitation service impact on physical function and adherence compared to face-to-face rehabilitation in patients with stroke: A systematic review and meta-analysis. *PM & R : the journal of injury, function, and rehabilitation* **2023**, doi:doi:https://dx.doi.org/10.1002/pmrj.12988.
27. Qin, P.; Cai, C.; Chen, X.; Wei, X. Effect of home-based interventions on basic activities of daily living for patients who had a stroke: a systematic review with meta-analysis. *BMJ open* **2022**, *12*, e056045.
28. Lombardo, C.; Islam, M. Stroke survivors' acceptance and satisfaction of telerehabilitation delivery of physiotherapy services: a systematic review. *PHYSICAL THERAPY REVIEWS* **2023**, doi:doi:10.1080/10833196.2023.2271301.
