# Supplementary figures and images for: Telerehabilitation and Its Impact Following Stroke: An Umbrella Review of Systematic Reviews
Source: J Clin Med. 2024 Dec 26;14(1):50. doi: 10.3390/jcm14010050 (PMC11721391; doi:10.3390/jcm14010050)

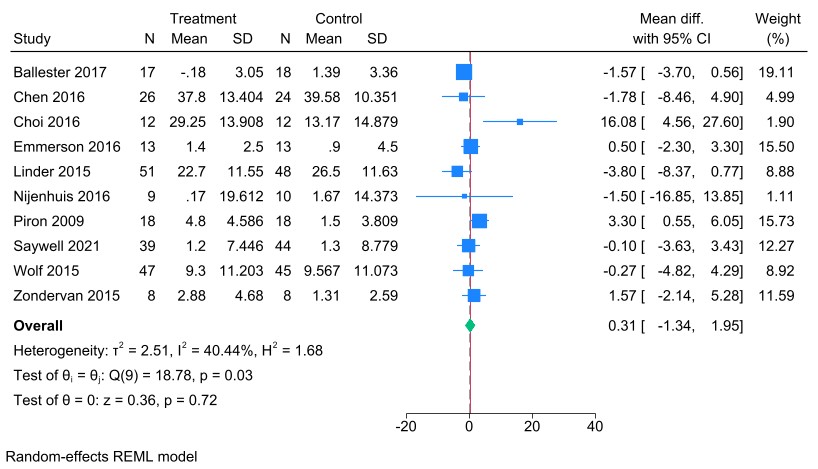

Supplement: Supplementary file 1 [file jcm-14-00050-s001.zip › Graph S1.jpg]

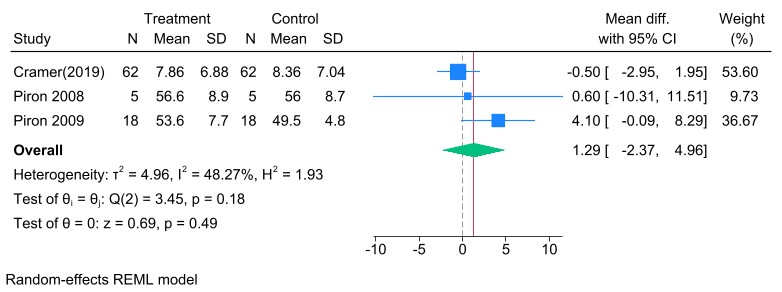

Supplement: Supplementary file 1 [file jcm-14-00050-s001.zip › Graph S2.jpg]
